# Early classification of spatio-temporal events using partial information

**DOI:** 10.1371/journal.pone.0236331

**Published:** 2020-08-05

**Authors:** Sevvandi Kandanaarachchi, Rob J. Hyndman, Kate Smith-Miles

**Affiliations:** 1 School of Science, Mathematical Sciences, RMIT University, Melbourne, Australia; 2 Department of Econometrics and Business Statistics, Monash University, Clayton, Australia; 3 School of Mathematics and Statistics, The University of Melbourne, Parkville, Australia; South China University of Technology, CHINA

## Abstract

This paper investigates event extraction and early event classification in contiguous spatio-temporal data streams, where events need to be classified using partial information, i.e. while the event is ongoing. The framework incorporates an event extraction algorithm and an early event classification algorithm. We apply this framework to synthetic and real problems and demonstrate its reliability and broad applicability. The algorithms and data are available in the R package *eventstream*, and other code in the supplementary material.

## 1 Introduction

Early detection and classification of emerging events in data streams is an important challenge in our data-rich world. Data streams may arise from many different applications including social media, Internet of Things, video surveillance, epidemiology and wireless sensors, to name a few. In each of these diverse applications, there are typically events that occur and are of interest because of their disruptive behaviour to the system.

In particular, we are interested in events that start, develop for some time, and stop at a certain time. Such events can be characterised by measurable properties or features, including the “age” of the event. It is a challenge to classify these events while they are still developing because only partial information is available at this stage. Once the events have stopped developing—when the events are finished—it is easier to classify them as the complete event features are now available. For example, it is easier to differentiate a daffodil from a tulip when both are in full bloom, but more difficult to differentiate a daffodil bud from a tulip bud without resorting to other information such as characteristics of leaves. Another example is identifying a network intrusion attack in its early stages. While it may be easier to identify a breach after it has happened, it is more difficult to identify which bits of network traffic is causing the breach while it is happening [1].

**Fig 1:**
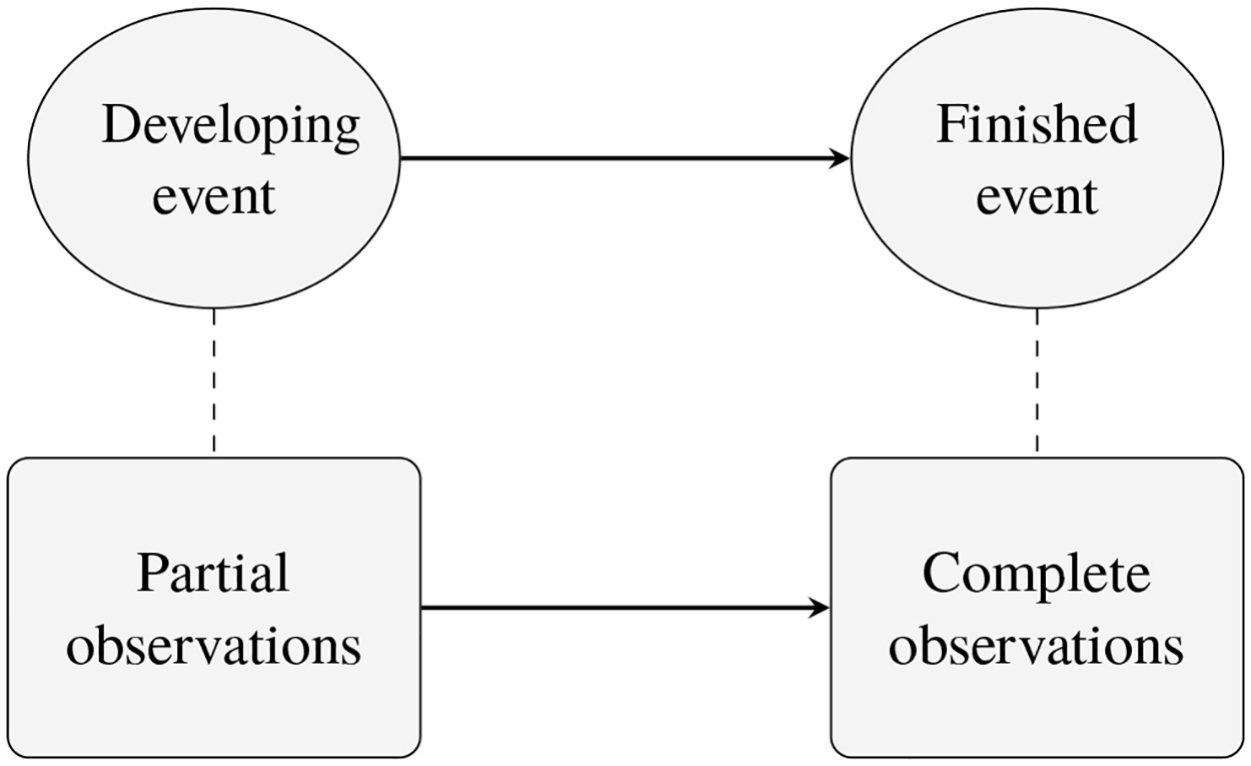
Event states and partial observations.

**Fig 2:**
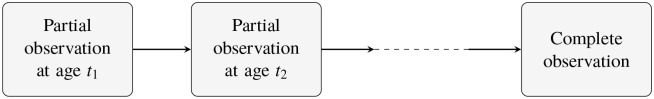
Partial observations growing with event-age.

In this regard, we can think of these events as having two states: developing and finished (Fig 1). The partial information contained in the developing events give rise to partial or premature observations, while the finished events give rise to complete observations. As the event develops, it gives rise to a series of partial observations—each partial observation encapsulating more information than its predecessor—culminating with the complete observation (Fig 2). Thus partial observations vary with the age of the event, the difference between the current time and the start time of the event. If early classification is important, one needs to take partial observations into account in the classification process. While event detection in data streams has received much attention from different disciplines ranging from video surveillance to social media [2, 3], there has been little exploration on developing/premature event classification to the best of our knowledge.

**Fig 3:**
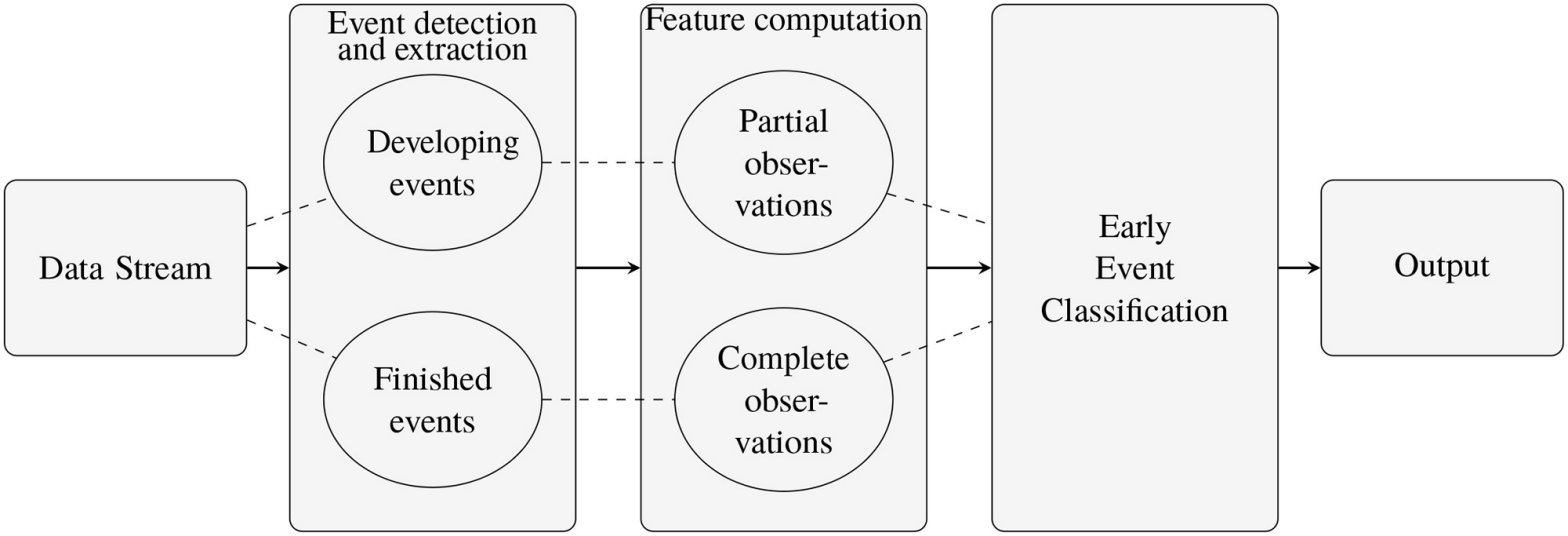
Framework for event extraction and classification for spatio-temporal data.

A general framework for event classification in data streams comprises different stages: 1. data pre-processing; 2. event detection and extraction; 3. feature computation; and 4. event classification. This framework, augmented with partial observations, gives the additional functionality of early event classification as depicted in Fig 3. In our framework we do not explicitly consider data pre-processing as a separate stage as this is highly dependent on the application.

Fundamentally, early event classification can be tackled by embedding age-varying coefficients in a learned model [4]. A linear model with age-varying coefficients is given by

$$\begin{array}{c} y_{t}=a_{0}\left(t\right)+a_{1}\left(t\right)x_{1}\left(t\right)+\cdots +a_{b}\left(t\right)x_{b}\left(t\right)+\varepsilon_{t}, \end{array}$$ (1)

where *y*_*t*_ is the output at age *t*, *a*_*i*_(*t*) are the age-varying coefficients, and *x*_*i*_(*t*) are the attributes of the event at age *t*; i.e. the partial/premature observation. A logistic model with age-varying coefficients is given by Eqs (1) and (2):

$$\begin{array}{c} z_{t}=e^{y_{t}}/\left(1+e^{y_{t}}\right), \end{array}$$ (2)

where *z*_*t*_ is the probability of the event being of a given class. As an event develops, the features *x*_*i*_(*t*) change with the age of the event, while keeping the class label constant. Thus, it is clear that the coefficients *a*_*i*_(*t*) need to change with the age of the event.

Concept drift [5] or non-stationarity of data streams [6] is different from age-varying events. For non-stationary data streams the distribution of data changes with time. For example consider a fixed part of a river, which is monitored for fluctuations in water volume and for animals. In months of heavy rains, the water volume increases changing the distribution compared to previous months. This is an example of non-stationarity. In contrast, age-varying events are about the extracted events and not the data-stream. To continue with the same example, consider a log appearing on this portion of the river. When the log comes closer and the image becomes clearer, suppose it becomes apparent that it is not a log, but a crocodile. This is an example of an age-varying event. Clearly, from the time when the log appeared to the time when it was detected that it was a crocodile, no significant changes in water volume or the animal distributions took place. The volume of water and the average number of crocodiles in the river does not need to change when the perception of the log changed to that of a crocodile as a result of more information. Thus age-varying events comprise change within the event as a result of maturing partial observations, while non-stationarity concerns change within the data stream.

### 1.1 Fibre optic cable example

**Fig 4:**
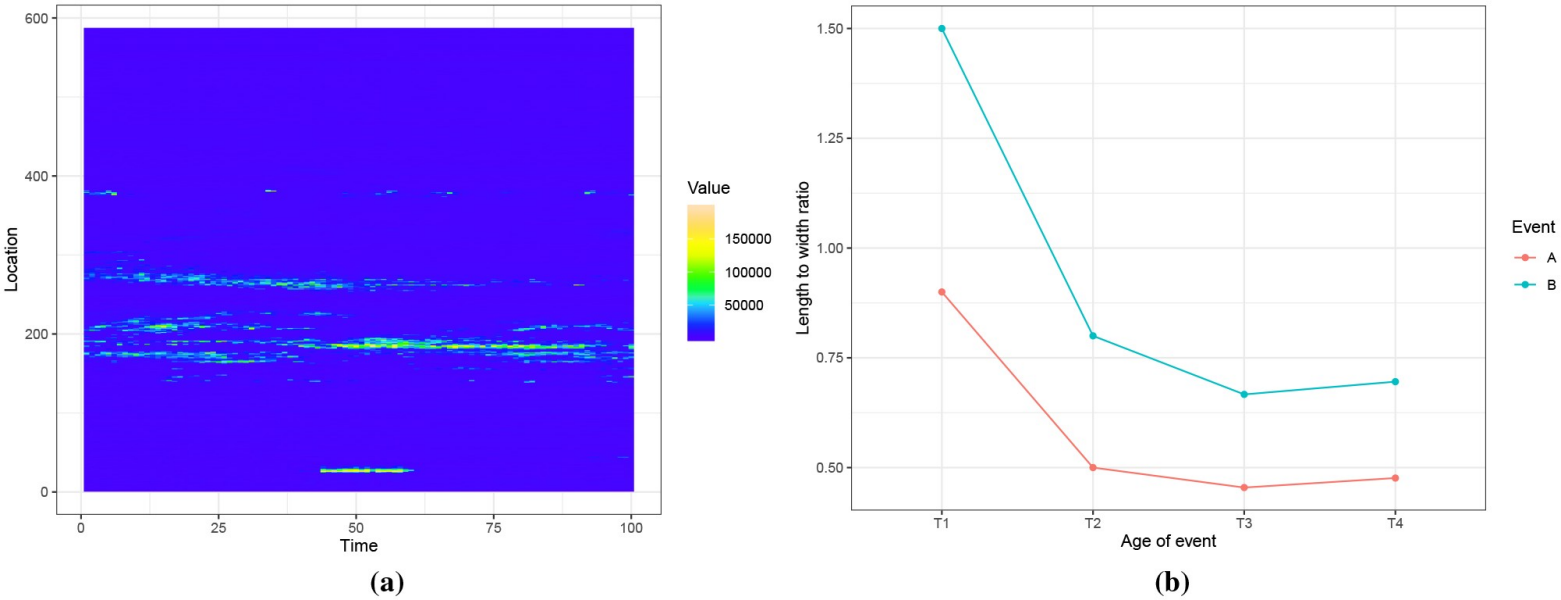
Fig 4a shows data from a fibre optic cable. We extract events from this dataset and compute event features. We consider two events belonging to two different classes and an event feature that changes with event-age. Fig 4b shows this event feature, which is the length to width ratio of the event, and how it changes with event-age.

Fig 4a shows the heatmap of a dataset produced from a fibre optic cable, illustrating age-varying events. A pulse is periodically sent through the cable and this results in a data matrix where each horizontal row gives the strength of the signal at a fixed location *x*_0_, and each vertical column gives the strength of the signal along the cable at a fixed time *t*_0_. In this dataset the yellow parts represent high intensity values and the blue parts represent low intensity values.

Fibre optic sensor cables are used in many applications including optical communications, detecting undersea cable faults [7], detecting oil leakages [8], detecting intruders on secured premises [9], and monitoring health of infra-structure such as bridges and pipe-lines [10]. Events in these applications can often be grouped into two classes. For example, a cable lying on the sea bed can produce spatio-temporal events that are either cable faults (A), or non-fault events due to the activity in the ocean (B). Due to its sensitivity, fibre optic cables are also prone to noise. In a setting where early classification is important, we need to classify these events quickly, preferably while they are still ongoing.

In the dataset in Fig 4a, events are seen in the lighter-coloured parts. The event at approximately location 30 between the time interval 45 to 60 is of class A while other events that appear between locations 150 and 400 are of class B. Fig 4b shows an event feature, which is the length to width ratio of the event, computed on two events belonging to each class. As the event matures, we see this feature change with event-age and also that no single threshold can differentiate between the two events for all event ages. Due to the commercially sensitive nature of the dataset, we refrain from giving details about the actual application.

Even for applications which can represent a dataset as an image, event classification is different from image classification. One difference is that an event is generally defined relative to the background signal, whereas in image classification it is generally not relative to the background. For example a flower is a flower regardless of the background in which it is taken. But a signal that is considered an event in a quiet background may not be considered an event in a noisy background. Another reason is that there is a natural grouping of objects in image classification. For example it is natural for an image with flowers to have leaves and stems that are associated in a certain way. It is unusual for parts of the stem to be disjoint and appear scattered over the image. However, in event detection and classification, there is no natural grouping of events, non-events and background noise. As a result, event extraction and classification are two separate stages of the general framework as shown in Fig 3.

Furthermore, we have not used deep learning methods such as LSTM for the following reasons. 1. For the fibre-optic application, the whole dataset, shown in Fig 7, is too small to train and test a deep neural net. 2 Even if the amount of data is not a limitation, the hyperparameter tuning of a deep neural net is an additional challenge that needs to be addressed from application to application. With our proposed model we do not have this problem. 3. Deep neural nets have serious limitations such as being vulnerable to adversarial samples [11], learning spurious features that do not align with human perception [12] and performing poorly on out-of-distribution samples [13]. This makes their use problematic in applications such as intrusion detection.

### 1.2 Contributions

We propose the framework depicted in Fig 3, which is summarized in Algorithm 1, for early event detection, extraction and classification in contiguous spatio-temporal data streams using the partial observation structure.

Specifically, our contributions in this paper are:

1. We introduce an algorithm for event detection and extraction from contiguous spatio-temporal data. We use change point analysis and density based clustering to detect events. We call this algorithm Change-Point Density-Based Event Extraction (CPDBEE).
2. We introduce a partial observations classifier suitable for early event classification. This classifier comprises multiple base classifiers, which are connected together using *L*_2_ penalty terms. We refer to this Connected Classifier as CC.
3. We demonstrate the validity of these algorithms on synthetic and real data and make the algorithms and data available in the R package *eventstream* [14]

**Algorithm 1**: *Early event extraction and classification framework*.

**input**: a 2 or 3-dimensional array denoting contiguous spatio-temporal data.

**output**: events, event features and early classification results

1 Detect and extract developing and complete events from the data stream using CPDBEE (Sections 4 and 5).

2 Compute event features. These are either partial or complete features computed from the extracted events (Section 6.1).

3 Use the Connected Classifier CC for early classification of events (Sections 6.3–7).

The remainder of the paper is organised as follows. Section 2 discusses related work in event detection, extraction and classification. In Section 3 we introduce the datasets: synthetic data, fibre optic cable data, of which a portion is shown in Fig 4, and NO_2_ data from NASA’s NEO (*NASA Earth Observations*, 2004) website. We use all these datasets to demonstrate the effectiveness of the proposed event extraction and classification algorithms in subsequent Sections. We introduce our event detection and extraction algorithm CPDBEE in Section 4 and discuss event extraction results in Section 5. Section 6 presents the early classification framework by starting with event features in Section 6.1, followed by an explanation of partial observations in Section 6.2, and culminating with the connected classifier CC in Section 6.3. We discuss the early classification results in Section 7 and present our conclusions and discuss future work in Section 8. Section 9 gives details on S1 File, which can be used to reproduce the results and S1 Appendix gives additional graphs of CPDBEE results.

## 2 Related work and their applicability

Spatio-temporal event detection is studied in many application related research areas such as epidemiology [15], deforestation [16], video streaming [17], and social media research [18]. In these applications the focus is on detecting “events of interest”. For some applications events of interest are rare events, while for others they are specific events, which match certain criteria [17]. Typically these events form a subgroup of data rather than a single data-point and their early detection has a strong societal impact [19].

### 2.1 Change-point detection

Univariate event detection has much overlap with change-point detection methods in time series [20]. Killick et al. [21] introduces change-point analysis as “the identification of points within a dataset where statistical properties change”. They formally consider a time series *y*_1:*n*_ = (*y*_1_, …, *y*_*n*_) with *m* change-points *τ*_1:*m*_ = (*τ*_1_, …, *τ*_*m*_), with *τ*_*i*_ < *τ*_*j*_ for *i* < *j*, resulting in *m* + 1 segments of the time series with the *i*^th^ segment containing $y_{\left(\tau_{i-1}+1\right):\tau_{i}}$. They identify change-points by minimizing

$$\begin{array}{c} \sum_{i=1}^{m+1}C(y_{\left(\tau_{i-1}+1\right):\tau_{i}})+\beta m, \end{array}$$

where C is the cost function for a segment and *βm* is the penalty term for having *m* segments. An example cost function is the negative log-likelihood. Their method PELT identifies change-points with linear computational time.

Multivariate change-point detection extends this framework to multiple time series measuring different quantities. Bardwell et al. [22] consider multivariate change-point detection in a panel data setting. They define $G_{i}\left(r\right)$ as the cost of segmenting time series *i* with the most recent change point *r* and minimize a penalized version of

$$\begin{array}{c} C_{K}=\underset{I_{1},\ldots ,I_{K}\;r_{1},\ldots ,r_{K}}{\text{min}}\sum_{k=1}^{K}\sum_{i\in I_{k}}G_{i}\left(r_{k}\right), \end{array}$$

where *K* denotes the number of change-points, *I*_*k*_ ⊂ {1, 2, …, *N*} and *N* the number of time series, such that for all time series *i* ∈ *I*_*k*_ the most recent change-point is located at *r*_*k*_.

Even though change-point detection methods detect changes, they do not generally identify a subset of changed observations, i.e. they do not perform event extraction. For our applications we need event detection as well as event extraction.

### 2.2 Scan statistics

In epidemiology, the scan statistic introduced by Kulldorff [15] and its later versions [23, 24] have gained much popularity. Using patient counts for each zip-code or similar region, the scan statistics approach detects events or clusters of interest, which may correspond to regions affected by a disease outbreak. The underlying assumption is that a true event will significantly increase patient counts, which is not accounted for by seasonality effects or random noise. Thus, events detected by the scan statistic approach are candidate regions for disease outbreaks.

The spatial scan statistic model [15] considers the null hypothesis *H*_0_ representing no events and alternative hypotheses *H*_1_(*S*) representing an event in a region *S* for some *S*. They compute the score function

$$\begin{array}{c} F\left(S\right)=\frac{\text{Pr}[\text{Data}|H_{1}\left(S\right)]}{\text{Pr}[\text{Data}|H_{0}\left(S\right)]} \end{array}$$

using Bernoulli and Poisson models, for different regions *S*, with circular scanning windows of varying radii centred at each spatial location. To account for multiple hypotheses testing, they conduct Monte Carlo simulations. They perform 9999 replications of the dataset under the null hypothesis and compute the test statistic for each replicated dataset and region *S*. Then they rank the actual test statistic for region *S* with the replicated test statistics and consider the actual to be significant if it is within the top 5% of replicated test statistic values. This is a time intensive algorithm.

The focus here is mainly on event detection and extraction and not on event classification. For example every event detected may not correspond to a disease outbreak. There may be some other explanation for an event.

### 2.3 Deforestation studies

A popular use of Landsat images is the study of deforestation and changes in land cover [25]. Verbesselt et al. [26] discusses changes in land cover caused by 1. seasonal effects driven by annual temperature and rainfall patterns, 2. gradual changes such as forest regrowth after fire, or 3. abrupt changes caused by deforestation, bushfires or urbanisation. Detecting abrupt changes, while accounting for seasonal variations is an important research problem in this domain.

However, all detected changes may not be due to deforestation. In order to detect only deforestation, Hamunyela et al. [27] calibrate their change detection algorithm using training data. In their paper, they tune the detection algorithm to capture certain activities of interest, i.e. detection and classification are performed as a single task.

The study conducted by Zhu and Woodcock [28] considers detection and classification as two separate tasks. After detecting changes, they classify the land cover (not the event) using a Random Forest classifier on the time series model coefficients. Furthermore, these studies do not consider event extraction; they treat each pixel separately and report results at a pixel level.

Another related research area is traffic incident studies, which are used to enhance transportation safety and security in a variety of ways including the identification of accident hotspots and factors contributing to vehicle crashes [e.g., [29]. Additionally, social media research [30] also investigates event detection. However, their focus is on text analysis and related techniques, which is quite different from ours.

### 2.4 Detection, extraction and classification

In our framework, event detection and extraction are different tasks from event classification. Events of interest—class A events in Section 1.1—may not necessarily have higher signal values compared to class B events as in disease outbreak scenarios. Furthermore, it is not desirable to improve the accuracy of the event extraction algorithm at the expense of missing class A events. Missing a class A event has a much higher cost than detecting a non-event for applications such as intrusion detection. Moreover, some applications require faster response times than is feasible by scan statistics methods.

Unlike in deforestation studies, analysis at a pixel level is not beneficial for the fibre optic application discussed in Section 1.1. A contiguous block of space-time pixels comprising an event needs to be considered for effective classification. Furthermore, even applications that consider event classification as well as extraction do not modify the original classifier to suit partial observations. This may be partly because they do not classify the event while it is taking place.

## 3 Applications and datasets used

We use three sets of datasets to evaluate the event extraction and classification algorithms: synthetic data, fibre optic cable data and NO_2_ data.

### 3.1 Synthetic data

The synthetic data was motivated from the fibre optic application and can be generated using the R package *eventstream*. The synthetic data contains events of two classes: A and B. All events belonging to class A look similar, that is they have one single non-standard shape or visual pattern. In contrast, events belonging to class B can have one of three different non-standard shapes, including the shape of events of class A. This is a characteristic of the fibre-optic application data, which prevents effective early classification of events based on shape alone.

**Fig 5:**
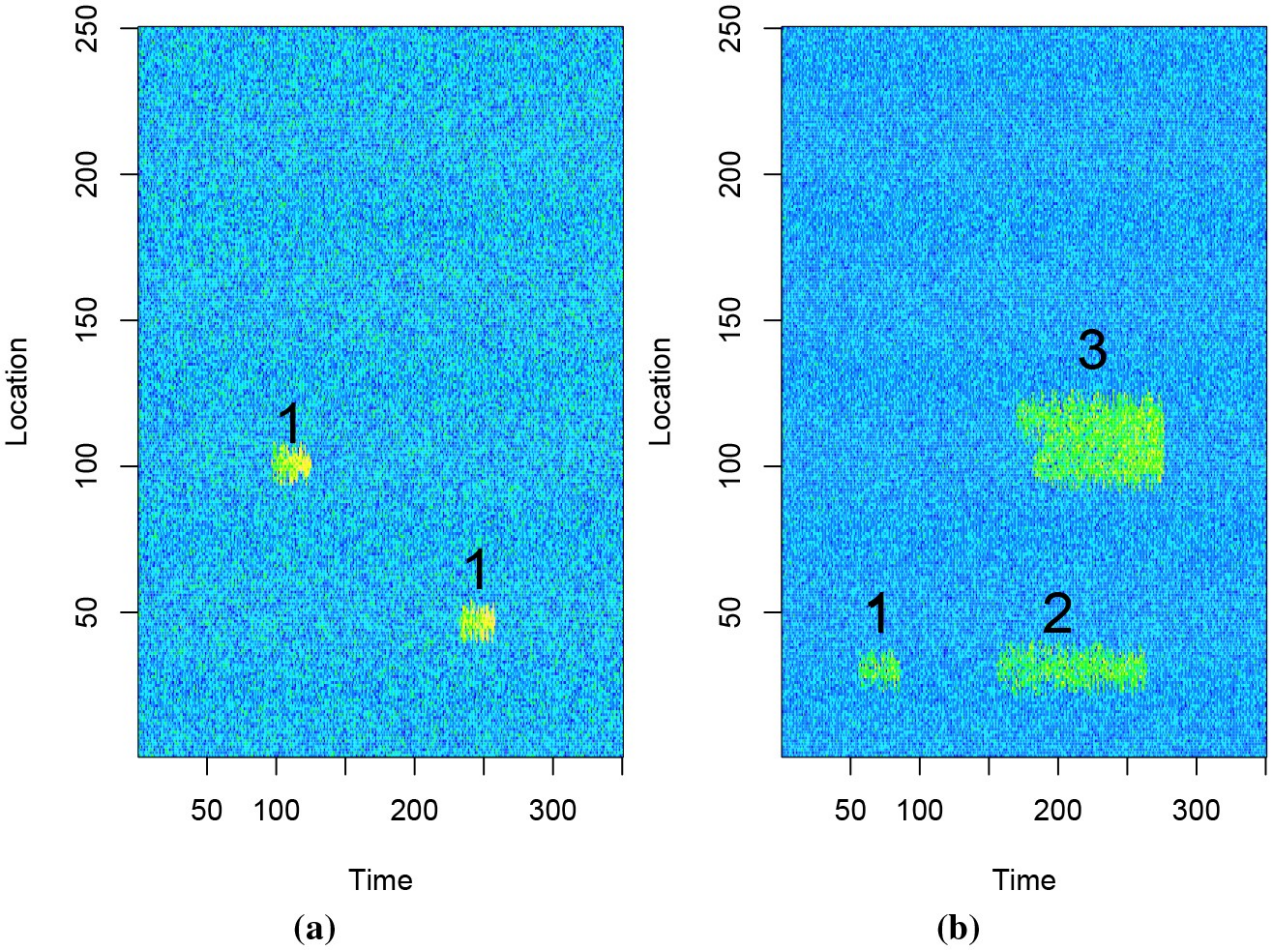
Class A events in Fig 5a and Class B events in Fig 5b.

Fig 5a contains two events of class A, and Fig 5b contains 3 events of class B. The shapes are labelled as 1, 2 or 3 in both Fig 5a and 5b, with shape 1 being the common shape.

The number of events of class A and B, and their positions, are randomly generated. The other difference between the events of class A and B, apart from the shape, is that values of the pixels belonging to events of class A and B come from different probability distributions. For both classes the intensity of pixel values increase linearly with the age of the event. We list the differences between class A and B events in Table 1.

**Table 1:** Differences in class A and class B events.

Feature	Class A value distribution	Class B value distribution
Starting cell/pixel values	N(4,2)	N(3,3)
Ending cell/pixel values	N(8,2)	N(5,3)
Maximum age of event: shape 1	U(20,30)	U(20,30)
Maximum age of event: shape 2	–	U(100,150)
Maximum age of event: shape 3	–	U(100,150)
Maximum location width of event: shape 1	U(20,26)	U(20,26)
Maximum location width of event: shape 2	–	U(30,38)
Maximum location width of event: shape 3	–	U(50,58)

**Fig 6:**
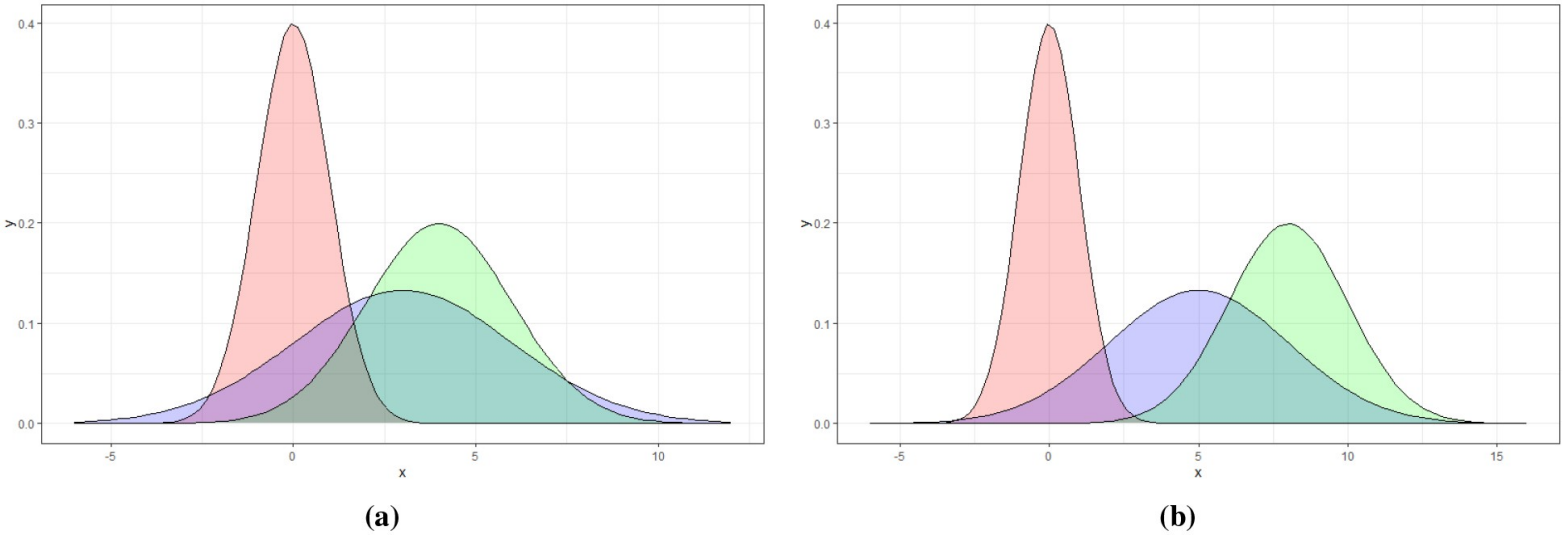
The probability distributions of the background and event A and B pixels. Fig 6a shows the starting distribution of event B pixels distributed as N(3,3) in blue and event A pixels N(4,2) in green. Fig 6b shows the ending distribution of event B pixels N(5,3) in blue and event A pixels N(8,2) in green. In both figures the background pixel distribution N(0,1) is shown in red.

These events are buried in a background of white noise, i.e. pixels having a probability distribution N(0,1). Fig 6 shows the starting and ending distributions for event A and B pixels along with the background pixel distribution.

### 3.2 Fibre optic cable data

**Fig 7:**
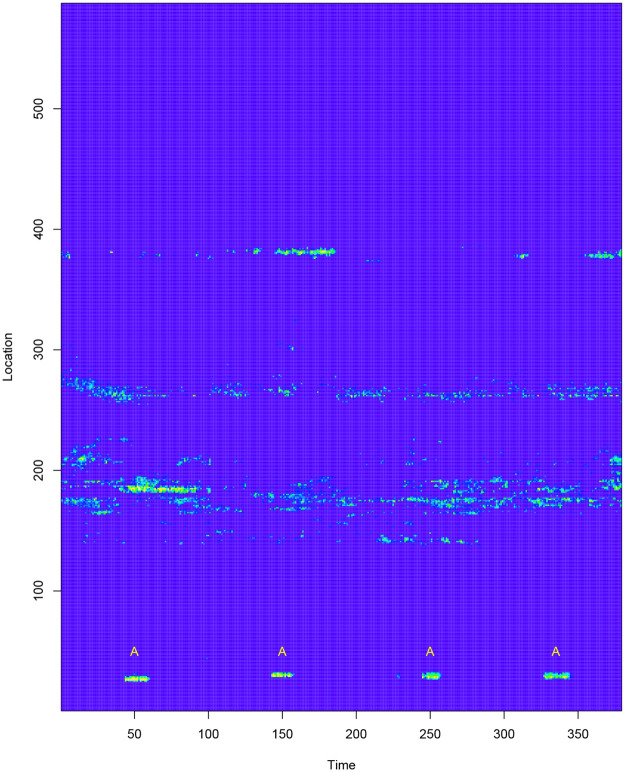
Data stream from a fibre optic cable.

The data for the first real application is from a fibre optic cable, and is shown in Fig 7. The data set is available in the R package *eventstream*. Again, for commercially sensitive reasons, we cannot provide more information about the application. The data set has dimensions 379 × 587, with class A events labeled with letter **A**. All other events belong to class B. These events are buried in noise. All blue colored pixels in Fig 7 have values lower than the yellow colored pixels and have random fluctuations of unknown distribution.

### 3.3 Nitrogen dioxide monitoring

The second real world application uses Nitrogen Dioxide (NO_2_) data obtained from NASA’s NEO website [31]. Nitrogen Dioxide is a major factor of air pollution [32], which causes approximately 7 million deaths per year according to the World Health Organisation [33].

The Ozone Monitoring Instrument (OMI) [34] aboard the Aura satellite records a variety of air quality measures including NO_2_ concentrations around the world. This is a 3-dimensional data stream with two spatial and one time dimension.

**Fig 8:**
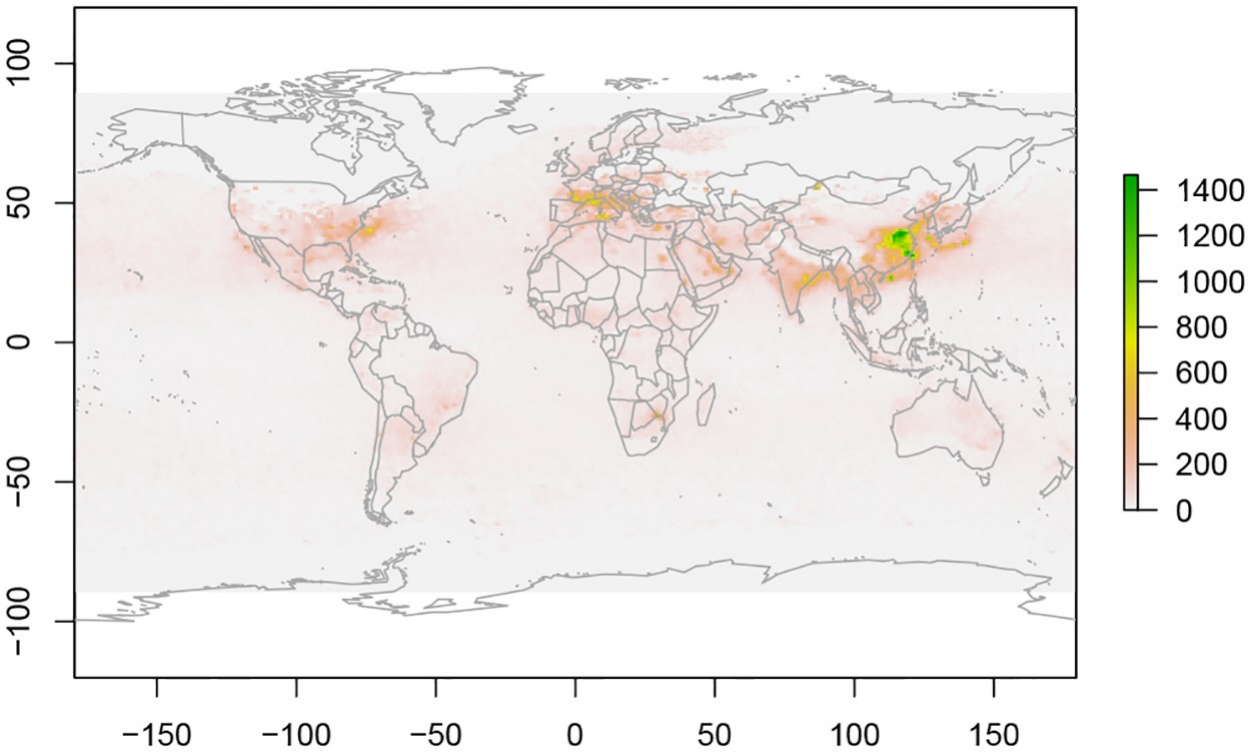
NO_2_ data from March 2018.

We use OMI NO_2_ monthly data from March to June for 10 years from 2010 to 2019 to detect and classify NO_2_ clusters. For each month the data comes in a matrix of 180 × 360 dimensions. The OMI NO_2_ data for March 2018 is shown in Fig 8.

## 4 Event detection and extraction

We extract events from data streams of two or three dimensions having one time dimension and one or two spatial dimensions. We employ a method for event extraction using change point detection [35] and DBSCAN [36], which is a density based clustering algorithm. Change point detection is used for event detection purposes and clustering for event extraction purposes.

### 4.1 Event detection

Change point detection in time series is a well studied topic as seen from the survey by Aminikhanghahi and Cook [37]. Killick and Eckley [35] discuss the R package *changepoint*, which includes three change point detection algorithms: a binary segmentation algorithm [38, 39], a segment neighborhood algorithm [40, 41] and PELT [21]. These algorithms are capable of detecting structural changes in time series based on mean and/or variance.

As we work with two or three-dimensional data streams we transform the data to suit univariate change point detection methods described in the R package *changepoint*. For a two-dimensional dataset we perform Principal Component Analysis (PCA) twice on the data similar to Sadia et al. [42]. Consider, a dataset *X*_*n*×*t*_ having *n* contiguous spatial points and *t* equi-distant time points. First we consider each location as an observation and perform PCA on *X*_*n*×*t*_. We are interested in the first set of PC scores of this analysis. Second, we consider each time point as an observation and perform PCA on *X*^*T*^. For each analysis, we consider the first set of PC scores and find change points using PELT as it is faster.

For a three-dimensional dataset *X*_*n*×*m*×*l*_ we compute averages ${\tilde{X}}_{n\times m}$ and ${\tilde{X}}_{m\times l}$ and perform PCA twice on each of these averaged matrices as in the two-dimensional case.

**Fig 9:**
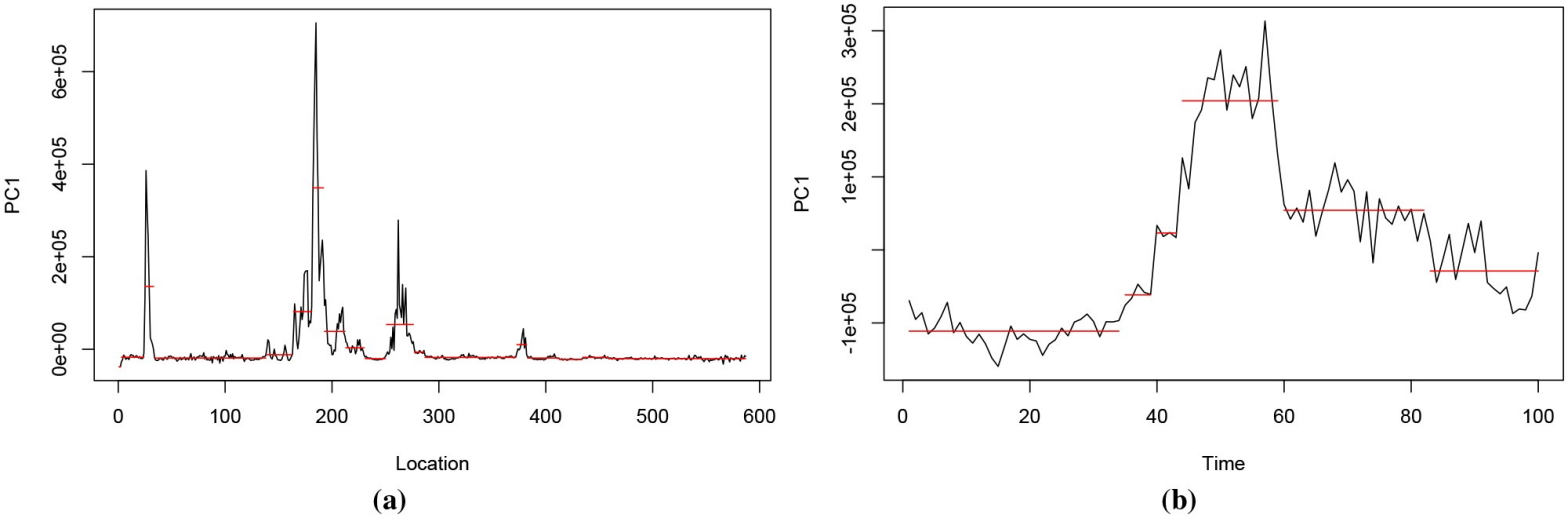
Change points of the first PC scores of the dataset in Fig 4. Fig 9a shows the first PC scores when taking each location as an observation. The horizontal red lines denote the levels and the change points correspond to the breaks or discontinuities of levels. Fig 9b shows the first PC scores when taking each time point as an observation and the associated change points.

**Fig 10:**
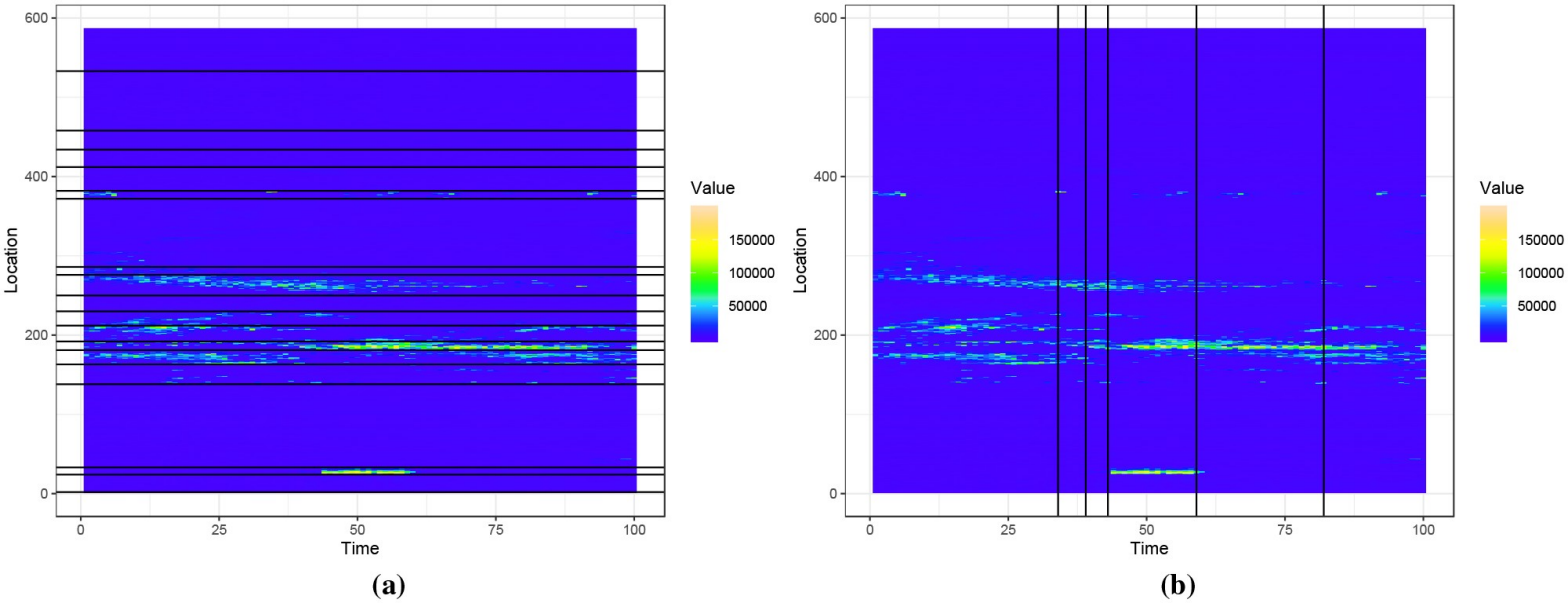
Location change points in Fig 10a and time change points in Fig 10b.

Fig 9 shows the coordinates of the first PC vector of the dataset illustrated in Fig 4. This dataset has 587 contiguous location points and 100 time points, and can be denoted as *X*_587×100_. By performing PCA on *X*, we obtain 100 PC vectors for 587 observations, where each observation denotes a location. We consider the coordinates of these observations in the direction of first PC vector, i.e. the first set of PC scores, and find their change points. PELT detects the following location change points: 2, 24, 33, 138, 163, 181, 192, 212, 230, 250, 276, 286, 372, 382, 412, 434, 458 and 533. Fig 9a illustrates the first set of PC scores and the location change points. Similarly, performing PCA on *X*^*T*^ considers each time point as an observation. PELT detects time change points at 34, 39, 43, 59 and 82 using the first set of PC scores of *X*^*T*^. Fig 9b illustrates the first set of PC scores and the associated time change points. Fig 10 shows the time and location change points as vertical and horizontal lines drawn on the heatmap of this dataset.

We see that the class A event at location 30 between time intervals 45 and 60 is detected by PELT in time and location using the first set of PC scores. In addition, the events denoted by lighter-coloured parts between locations 150 and 300 are also detected by location change points. However, the location change points that are greater than 400 do not correspond to any lighter-coloured parts in Fig 10a. In our framework summarized in Algorithm 1, event extraction precedes event classification. Thus, it is preferred to detect and extract candidate events which may not correspond to real events, rather than employ stringent event extraction methods and miss real events, i.e. type 1 errors are preferred at the event extraction stage.

### 4.2 Event extraction

Once the events are detected the next task is to extract them. For the dataset illustrated in Fig 4, the true events are light-coloured contiguous parts, which have higher signal values than the background. The change points computed in Section 4.1 alone are not sufficient to extract these events as seen in Fig 10. Clustering is a tool that is often used in event extraction [43]. We use DBSCAN clustering in our event extraction process.

To extract events we consider pixels which have high signal values, defined by a percentile *α*. That is, if *x*_*ij*_ is the signal value at (*i*, *j*) position of *X*, then we denote by *q* a signal value corresponding to the percentile *α*. The default value of *α* is 95%. We consider pixels *x*_*ij*_ greater than *q* and cluster these in time and location using DBSCAN. DBSCAN allocates pixels that are close to each other to the same cluster. These clusters are our candidate events. However, some candidate events may not have contributed to the change points discussed in Section 4.1. We are interested in candidate events that are detected by change points. Thus, if a time or location change point is detected within a candidate event or at the boundary of a candidate event, we consider that candidate event as a legitimate event. We discard candidate events which do not meet this criterion.

We summarize the event detection and extraction algorithm CPDBEE for two dimensional datasets in Algorithm 2.

**Algorithm 2**: *Algorithm CPDBEE for 2D datasets*.

**input**: a 2 dimensional matrix *X*_*n*×*m*_, and parameters *α*, *ϵ* and *minPts*.

**output**: events and event ids

1 Compute PCA on *X*_*n*×*m*_.

2 Let *z*_1_ denote the first set of PC scores of *X*.

3 Let *C*_1_ be the set of change points of *z*_1_ using PELT.

4 Compute PCA on *X*^*T*^.

5 Let *z*_2_ denote the first set of PC scores of *X*^*T*^.

6 Let *C*_2_ be the set of change points of *z*_2_ using PELT.

7 Let *q* denote the *α*-percentile of the signal values of *X*.

8 *S* = {(*i*, *j*) ∣ *x*_*ij*_ > *q*}. *S* is a matrix of 2 columns, which gives locations of *X*, which have signal values greater than the *α*^th^ percentile.

9 Let *X*(*S*) be signal values of *X* in *S* locations.

10 Using DBSCAN cluster *S* using *ϵ* and *minPts*.

11 This clustering gives each (*i*, *j*) ∈ *S* a cluster id. Noise points are given cluster id 0.

12 Let *T* be the vector of cluster ids for each (*i*, *j*) pair in *S*, i.e. the *k*^th^ row of *S* denotes a pixel location in cluster *T*(*k*).

13 Consider each cluster as a candidate event and the cluster id as the candidate event id.

14 Let *S*_1_ be the first column of *S*, i.e. *S*_1_ has the first coordinate of each pair (*i*, *j*) in *S*. Similarly let *S*_2_ denote the second column of *S*.

15 Let *I*_1_ = *S*_1_ ∩ *C*_1_. These are *x*_1_ positions of candidate events that are change points.

16 Let *I*_2_ = *S*_2_ ∩ *C*_2_. These are *x*_2_ positions of candidate events that are change points.

17 Let *E* = {(*i*, *j*) ∈ *S* ∣ *i* ∈ *I*_1_ or *j* ∈ *I*_2_}. These are *x*_1_ or *x*_2_ positions of candidate events that are also change points.

18 Find candidate event ids *G* which do not have any change points in *E*.

19 /* For example the *k*^th^ candidate event may not have any pixels that are change points.     */

20 Remove these candidate events from *T* and *S*. The remaining clusters (*S*, *X*(*S*)) are considered events.

For a three dimensional dataset *X*_*n*×*m*×*l*_ with two spatial and one time dimension, DBSCAN clustering is performed on the three dimensional matrix to find the candidate events, and PCA is performed on two dimensional averaged matrices ${\bar{X}}_{n\times m}$ and ${\bar{X}}_{m\times l}$ to find the change points in each dimension. Candidate events which contribute to change points are considered events of the three dimensional dataset.

CPDBEE considers pixels which have high signal values for event extraction. For a different application such as deforestation, true event pixels may have lower values compared to the rest. For such applications, CPDBEE can be adapted to consider pixels that have signal values less than the percentile *α*, or alternatively, used in its current form by multiplying the dataset by −1.

For CPDBEE an event is a contiguous block of pixels in space and time. As such, for a certain application if two events overlap in space and time they will be considered as a single event. We test CPDBEE on balanced and imbalanced datasets with rare classes.

### 4.3 Algorithm complexity of CPDBEE

For an input data matrix *X*_*n*×*m*_, CPDBEE involves the following four main steps:

1. PCA involves $O(\text{min}(m^{3},n^{3}))$ operations [44].
2. PELT has a linear computational cost, i.e. O(n)+O(m) for finding changepoints of *z*_1_ and *z*_2_ in Algorithm 2.
3. Computing the *α*-percentile. Using quicksort yields an average performance of O(nm) as there are *nm* entries in *X*.
4. DBSCAN clustering has O((1-α)nmlog((1-α)nm)) performance.

This yields an overall complexity of $O(\text{max}(\text{min}\left(m^{3},n^{3}\right),\;\left(1-\alpha \right)nm\;\text{log}\left(\left(1-\alpha \right)nm\right)))$ for 2D datasets.

For a 3D dataset *X*_*n*×*m*×*l*_, CPDBEE includes the following steps:

1. Computing averaged matrices ${\tilde{X}}_{n\times m}$ and ${\tilde{X}}_{m\times l}$. This involves O(nml) simple operations.
2. Computing PCA on both ${\tilde{X}}_{n\times m}$ and ${\tilde{X}}_{m\times l}$ involves $O(\text{max}(\text{min}(m^{3},n^{3}),\;\text{min}(m^{3},l^{3})))$ operations.
3. Computing the *α*-percentile involves O(nml) as there are *nml* entries in *X*.
4. DBSCAN clustering involves O((1-α)nmllog((1-α)nml)).

This gives an overall complexity of $O(\text{max}(\text{min}(m^{3},n^{3}),\;\text{min}(m^{3},l^{3}),\;\left(1-\alpha \right)nml\;\text{log}\left(\left(1-\alpha \right)nml\right)))$ for 3D datasets.

In order to detect and extract partial events, we have implemented CPDBEE in a framework incorporating a moving window. This is analogous to loading data chunks into memory instead of the entire dataset. For large datasets the values *m* and *n* can be used to define the window size instead of the entire dataset.

#### 4.3.1 The parameters of CPDBEE

The algorithm CPDBEE has three parameters *α*, *ϵ* and *minPts* with the following defaults:

$$\begin{array}{ccc} \alpha =0.95, & \epsilon =5, & \text{and}\;minPts=10. \end{array}$$ (3)

The parameter *α* depends on the application. It can be roughly described as the proportion of data contributing to events. In our fibre optic example, events are rare and correspond to high signal values in the data matrix. As such we set *α* to a high percentile.

The parameters *ϵ* and *minPts* are DBSCAN parameters. The parameter *ϵ* describes the size of the *ϵ*-neighbourhood and *minPts* denotes the the minimum number of points in the *ϵ*-neighbourhood that are needed to make a cluster. DBSCAN has a default value of 5 for *minPts*, which we have increased to 10 as we are not interested in very small events. The value of *ϵ* is set to 5 because we would like to consider two high signal valued pixels that are 5 pixels apart as belonging to the same event.

To aid with parameter selection we provide tuning functionality to CPDBEE.

#### 4.3.2 The parameter selection for CPDBEE

**Fig 11:**
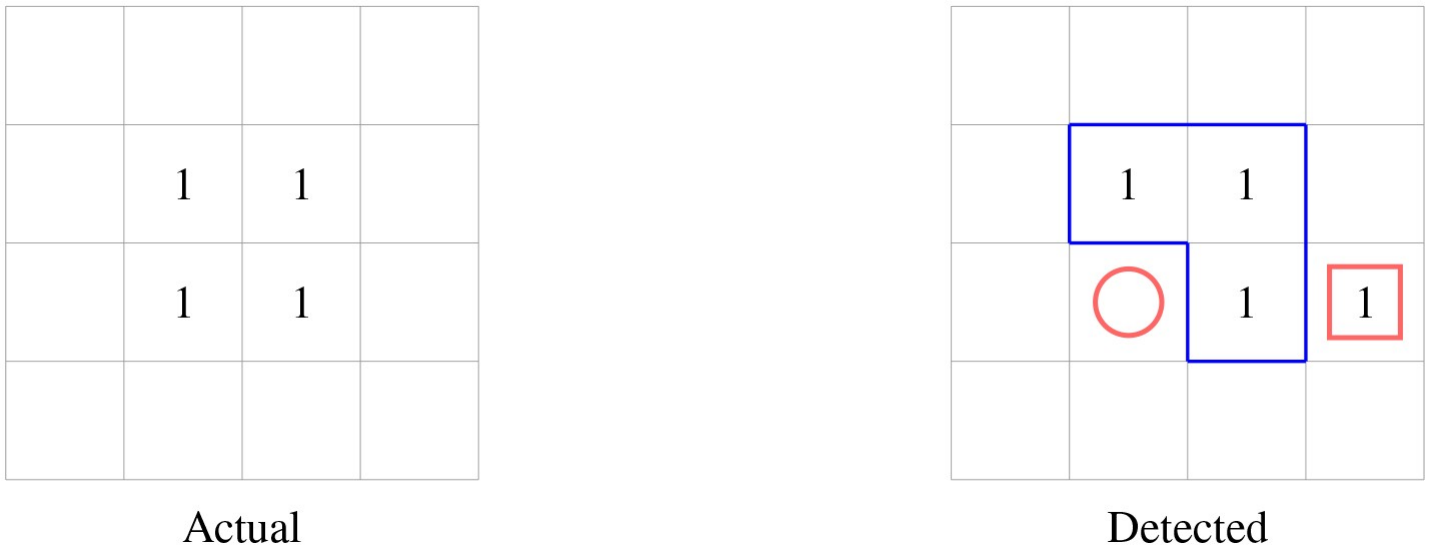
A toy example depicting an actual and detected event. The actual and detected event pixels are labeled as 1 with the intersection or true positives marked with a blue outline. The false positive and false negative pixels are marked with a red square and circle respectively. The Jaccard index for the detected event is 0.6.

As events are application specific, one set of parameters does not suit all applications. As such, using a small amount of labeled data we find the set of parameters that produce the best event detection outcome by computing the Jaccard Index [45] for a range of parameter values. The Jaccard index can be used to compare the similarity between two events and is defined as

$$\begin{array}{c} J\left(A,B\right)=\frac{\left|A\cap B\right|}{\left|A\cup B\right|}=\frac{\text{TP}}{\text{TP}+\text{FN}+\text{FP}}\;, \end{array}$$ (4)

where *A* and *B* denote the actual and the detected events and TP, FN and FP denote the number of true positives, false negatives and false positives. We illustrate this using a toy example in Fig 11. In this example we see that the number of true positives, false negatives and false positives are 3, 1 and 1 respectively, giving a Jaccard index of 0.6.

Using labeled data we compute the Jaccard index for a range of *α*, *ϵ* and minPts values and choose the combination of parameters that maximize the Jaccard index.

## 5 Event extraction results

### 5.1 Analysis of event extraction using synthetic data

In this Section we use synthetic data to analyse the event extraction algorithm. The reason for using synthetic data is because we know the true event locations. For fibre-optic and NO_2_ data, we have rough approximations of event locations, but not the exact locations.

#### 5.1.1 The effect of parameters on extracted events

**Fig 12:**
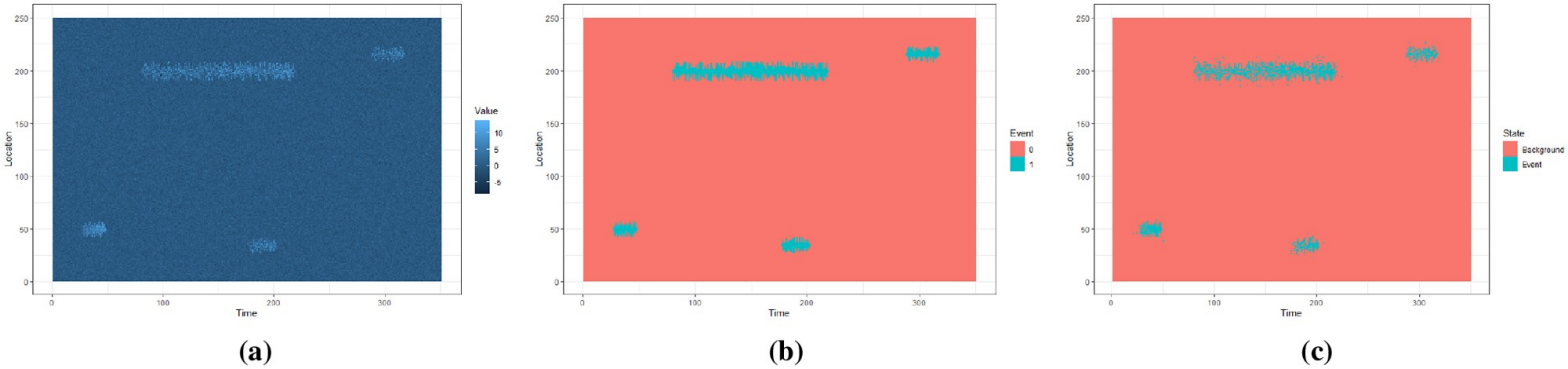
The synthetic data with buried events in Fig 12a. The true events in Fig 12b and extracted events for *α* = 0.95, *ϵ* = 5 and minPts = 10 in Fig 12c.

Using synthetic data, we explore the effect of parameters *α*, *ϵ* and MinPts on extracted events. We generate synthetic data shown in Fig 12 and extract events for a range of parameter values with *α* ∈ {0.90, 0.91, 0.92, 0.93, 0.94, 0.95, 0.96}, *ϵ* ∈ {4, 5, 6, 7, 8, 9} and minPts ∈ {4, 6, 8, 10, 12}. For each combination of *α*, *ϵ* and minPts we extract events from the dataset. As this is synthetic data we know the true event locations. Using this information we compute the Jaccard index for each set of extracted events with different parameters.

Fig 12a shows the raw data with events in light coloured parts. Fig 12b and 12c shows the actual events and the extracted events for *α* = 0.95, *ϵ* = 5 and minPts = 10.

**Fig 13:**
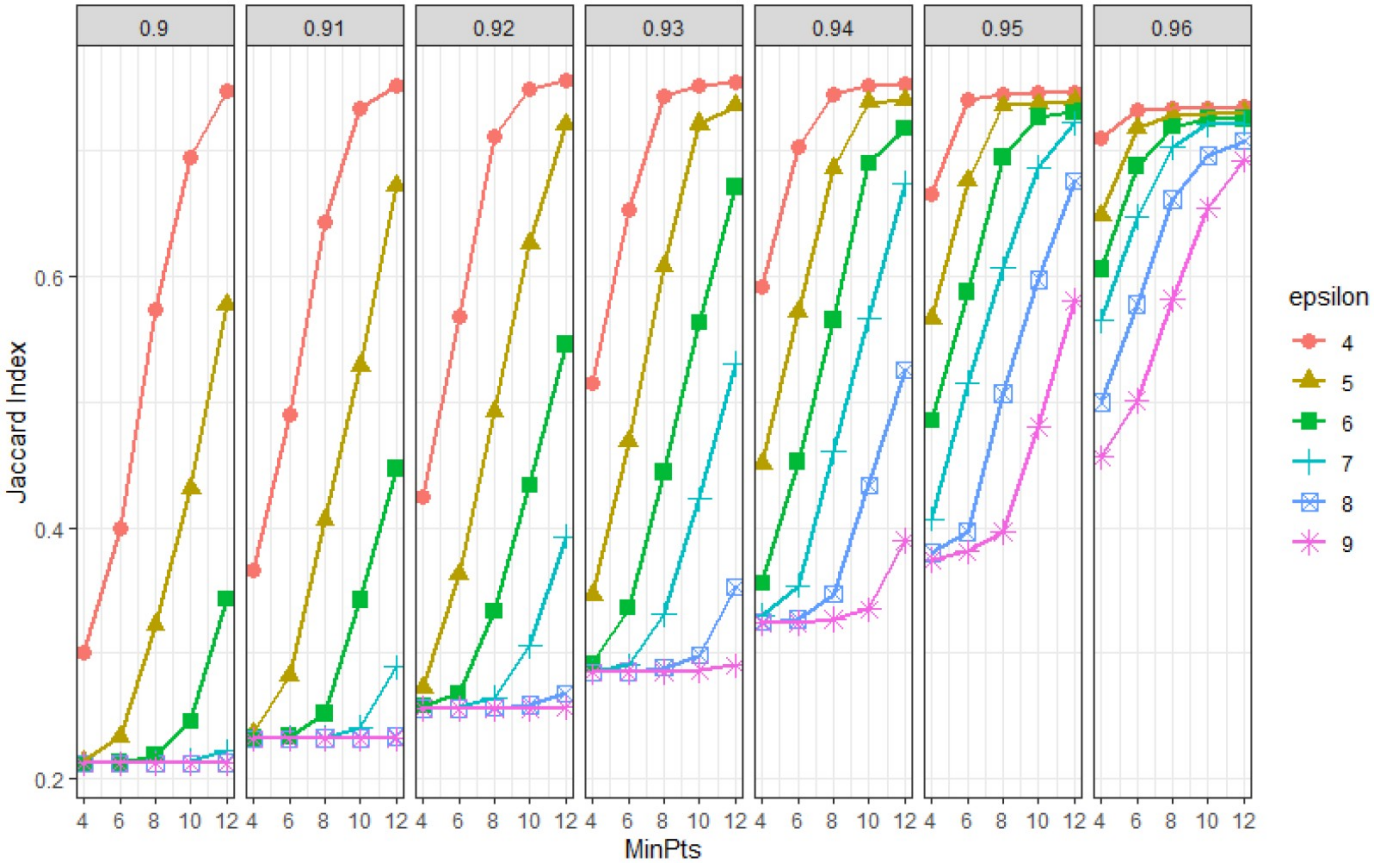
Comparison of CPDBEE parameters on synthetic data. The Jaccard index for different values of *α*, *ϵ*, and MinPts are plotted. Each pane has constant *α* ranging from 0.90 to 0.96. Each plotted curve has a fixed *ϵ* value. The *x* axis denotes MinPts and the *y* axis the Jaccard Index. A higher Jaccard index denotes better event output.

Fig 13 shows the comparison of Jaccard index for different values of *α*, *ϵ* and minPts. We see that the overall spread of the curves in terms of Jaccard index decrease as *α* increases. For fixed values of *α* and *ϵ* the Jaccard index increases with increasing minPts. In addition, for fixed *α* smaller *ϵ* gives better performances than bigger *ϵ* within the tested range. For the above analysis, Jaccard index reaches a maximum for *α* = 0.92, *ϵ* = 4 and minPts = 12.

#### 5.1.2 Early detection

**Fig 14:**
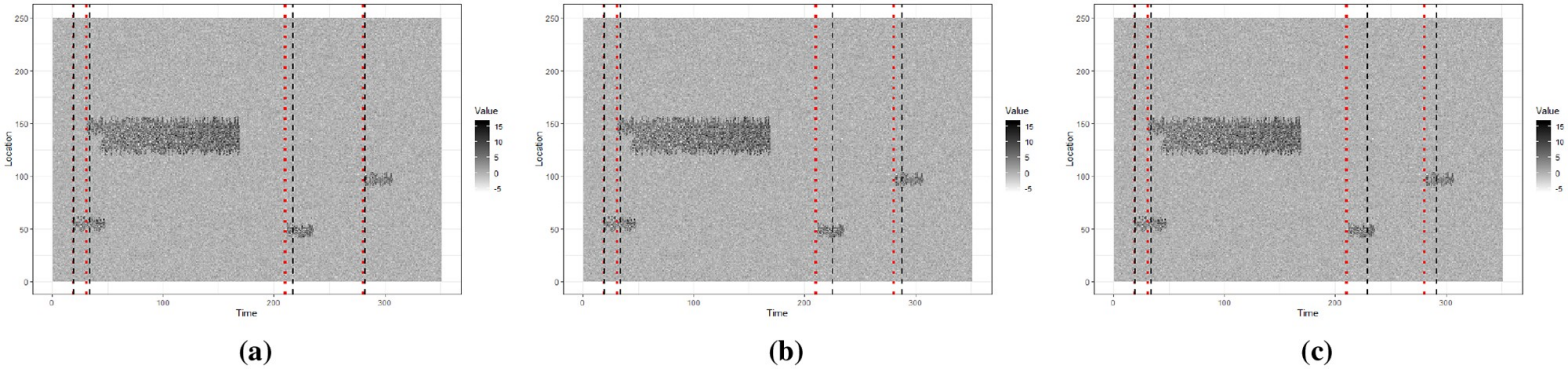
Events are extracted from synthetic data using a moving window model of width 50, 75 and 100 in Fig 14a, 14b and 14c respectively. The first time each event is detected is marked using a black dashed line. The actual start time of each event is marked using a red dotted line. For some events the time of detection and the actual start time is very close to each other, making it difficult to distinguish the dashed line and the dotted line.

In this Section we investigate how quickly an event can be detected using synthetic data. We use the synthetic data shown in Fig 14 and use a moving window model. As the first event starts at *t* = 19, we start with a window of width 15, i.e. *t* ∈ [1, 15] and increase the width by 1 until we reach a width of 50 i.e. *t* ∈ [1, 50]. Then we move the window by a single step in time, so that the next window contains *t* ∈ [2, 51]. We start with a window width of 15 so that we can check if we detect the event at *t* = 19 as soon as it develops. We detect and extract events in each window and compare the first time each event is detected with the actual time it starts. Fig 14a shows the actual time each event starts in red dotted lines and the time it is detected in black dashed lines. We repeat this process for window widths 75 and 100 and the results are illustrated in Fig 14b and 14c.

**Fig 15:**
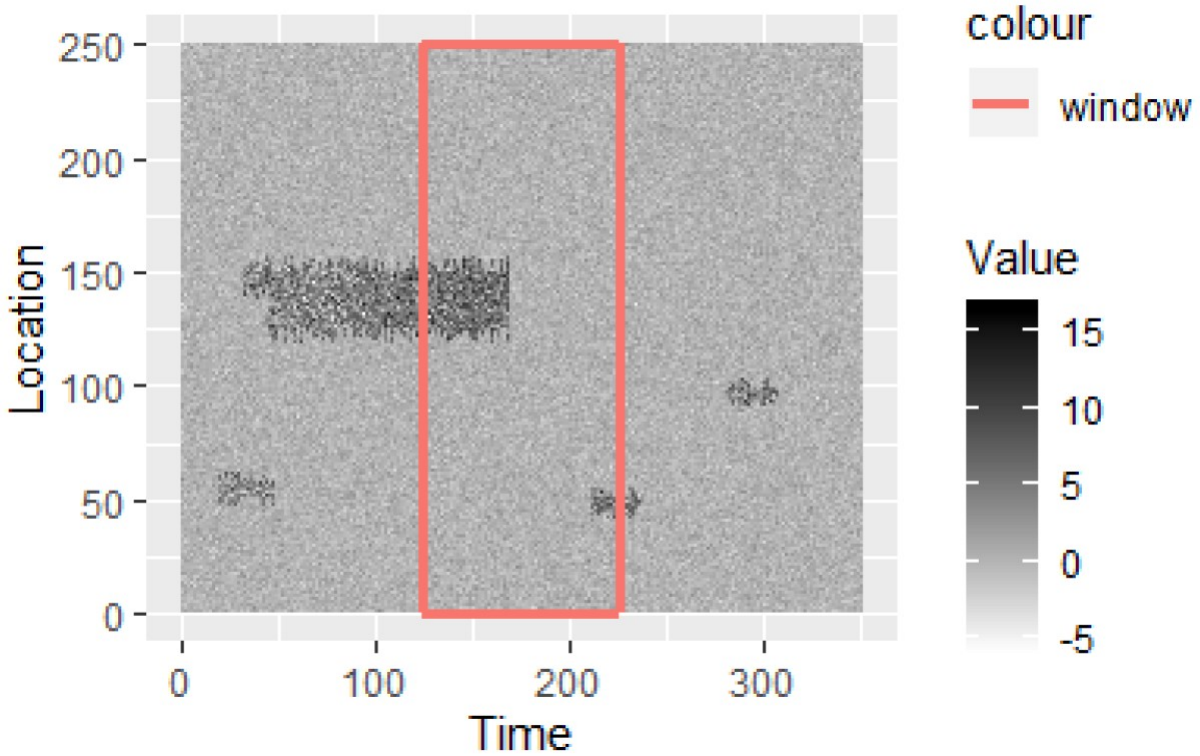
A window of width 100 including event 3 partially. This window includes the previous event which is large and more noisy compared to event 3.

Table 2 shows the actual event start time and the first detected time for different events and window width values. In particular, we see that event 3, which occurs at *t* = 210 gets detected quite late as the window size increases. This is because a bigger window includes the previous event, which is comparatively large and noisy, resulting in detecting event 3 later than other events (Fig 15). Indeed, a bigger window width can result in events getting detected later, compared to a smaller window width. However, a smaller window width can result in more false positives as well. As we tackle event classification separately, we do not concern ourselves about the false positives at this stage.

**Table 2:** Event start time and first detected time for different window sizes.

Event Id	Actual Start Time	First Detection Time for *w* = 50	First Detection Time for *w* = 75	First Detection Time for *w* = 100
1	19	20	20	20
2	31	34	34	34
3	210	217	225	229
4	280	282	288	291

Therefore, we see that in addition to CPDBEE parameters *α*, *ϵ* and minPts the width of the window and in particular the noisiness and the intensity of data in the window affects early detection of events.

**Fig 16:**
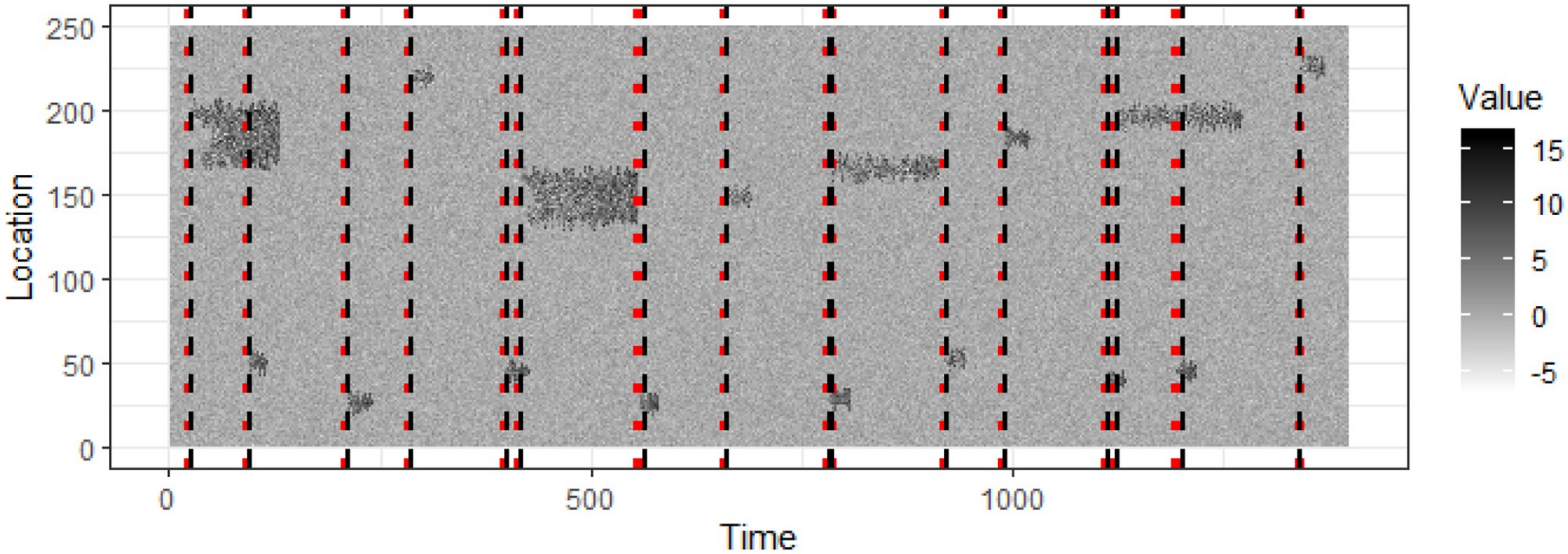
A longer synthetic data stream with 16 events. The actual start time of events is shown in red doted lines and the detected start time of events is shown in black dashed lines.

**Fig 17:**
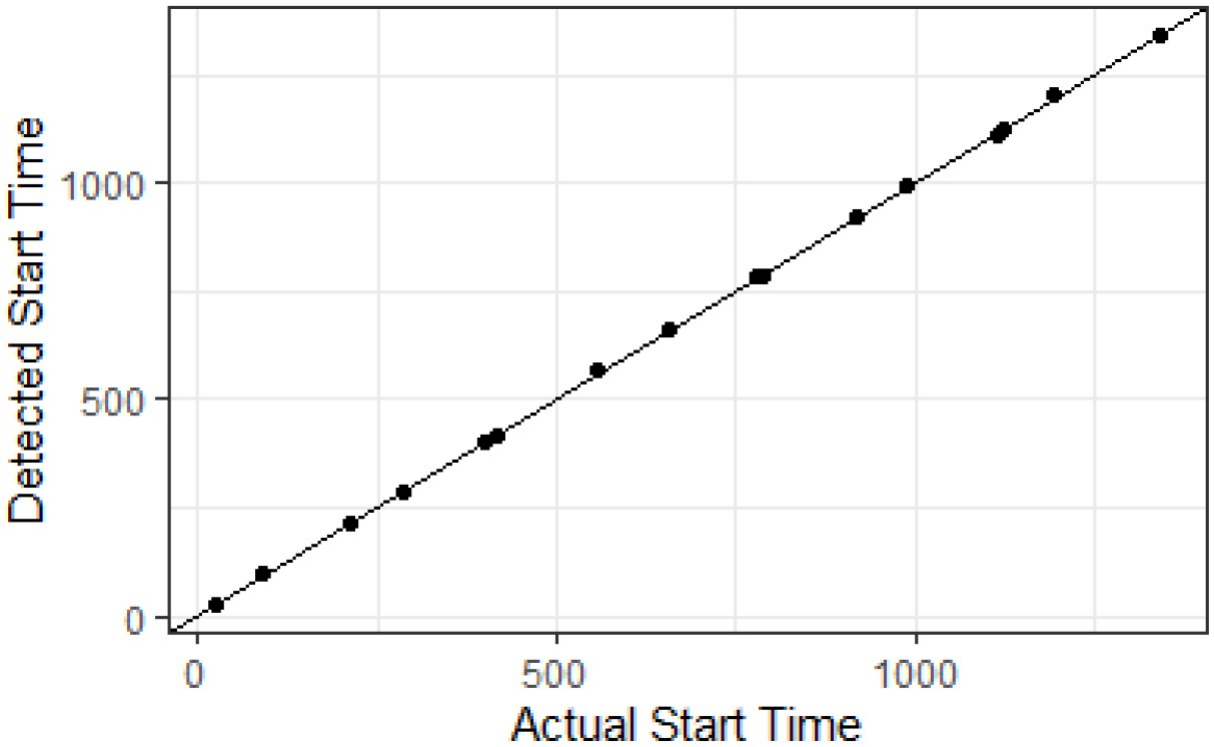
The actual and detected start time of events in Fig 16 with the line Actual = Detected.

To see how the event detection algorithm performs on fast-evolving events we generate a longer synthetic data stream shown in Fig 16, which includes 16 events. We detect events using a moving window of width 50 and mark the actual start of the events with red dotted lines and detected start of the events with black dashed lines. Fig 17 shows the actual and detected event start times with the line Actual = Detected. From Figs 16 and 17 we see that events are detected with a small time lag.

### 5.2 Sensitivity of CPDBEE

As illustrated in Fig 6, the synthetic data has different probability distributions for background, class A and B events. Class A event pixels have a starting distribution of N(4,2) while class B pixels start at N(3,3). The background pixels are distributed as N(0,1).

**Fig 18:**
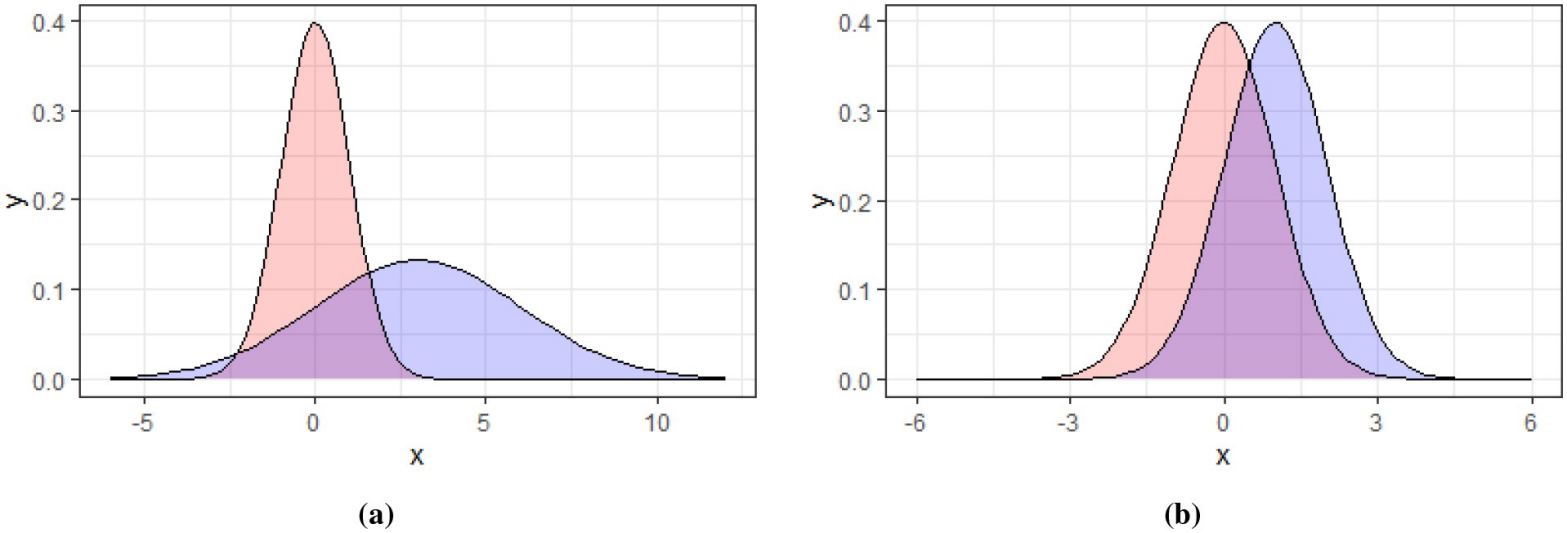
The probability distributions of the background and event pixels at start for two combinations of *μ* and *σ*. Fig 18a shows the starting distribution of event pixels distributed as N(3,3) in blue. Fig 6b shows another starting distribution of event pixels, N(1,1) in blue. In both figures the background pixel distribution, N(0,1) is shown in red.

**Fig 19:**
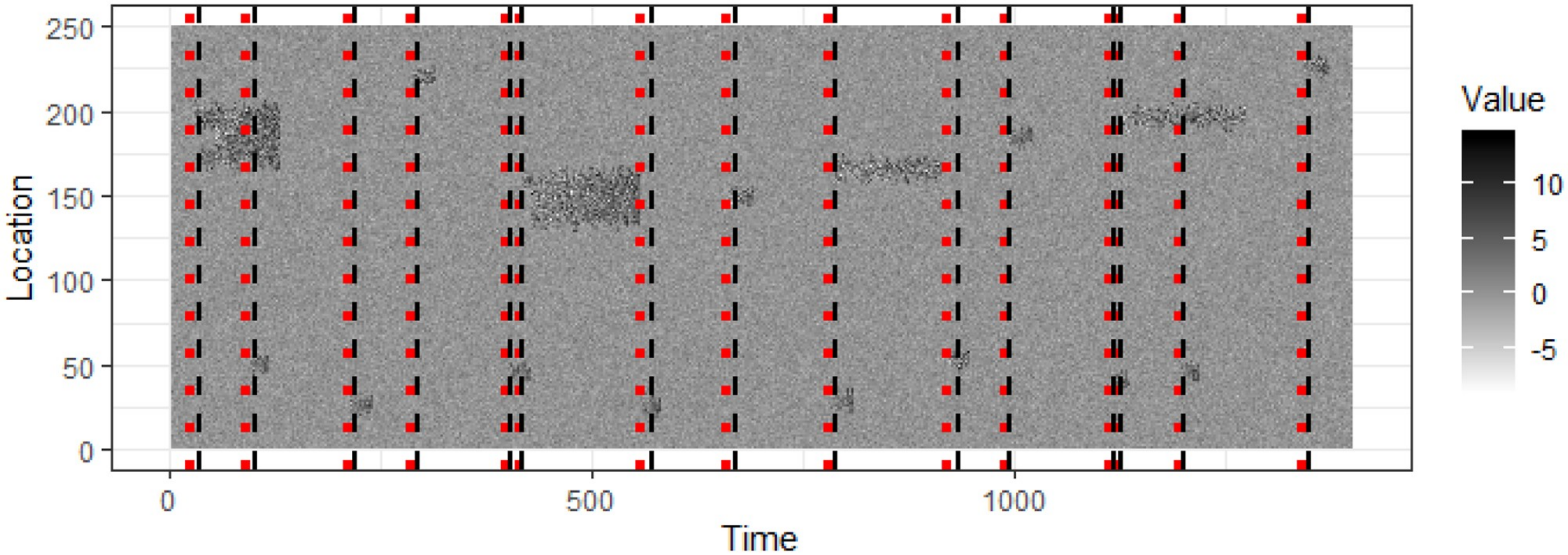
A longer synthetic data stream with 16 events with lower contrast between the events and the background. The actual start time of events is shown in red doted lines and the detected start time of events is shown in black dashed lines.

To test the sensitivity of the event detection algorithm we change the starting distribution of events A and B to be N(μ,σ) with *μ* ∈ {3, 2, 1} and *σ* ∈ {3, 2, 1}. The event pixel distribution N(3,3) and N(1,1) along with the background pixel distribution is shown in Fig 18. For each combination of *μ* and *σ* we generate a data stream similar to that in Fig 16 and record the detected start time using CPDBEE. Fig 19 shows the detected start times and actual start times for the weakest events when *μ* = *σ* = 1. The contrast between the events and the background is lower in Fig 19 compared to Fig 16 as the starting event distribution for both class A and B events is N(1,1). The actual start times are denoted by red dotted lines while the detected start times are denoted by black dashed lines. By close inspection we see that Fig 16 has shorter gaps between the actual and detected compared to Fig 19.

**Fig 20:**
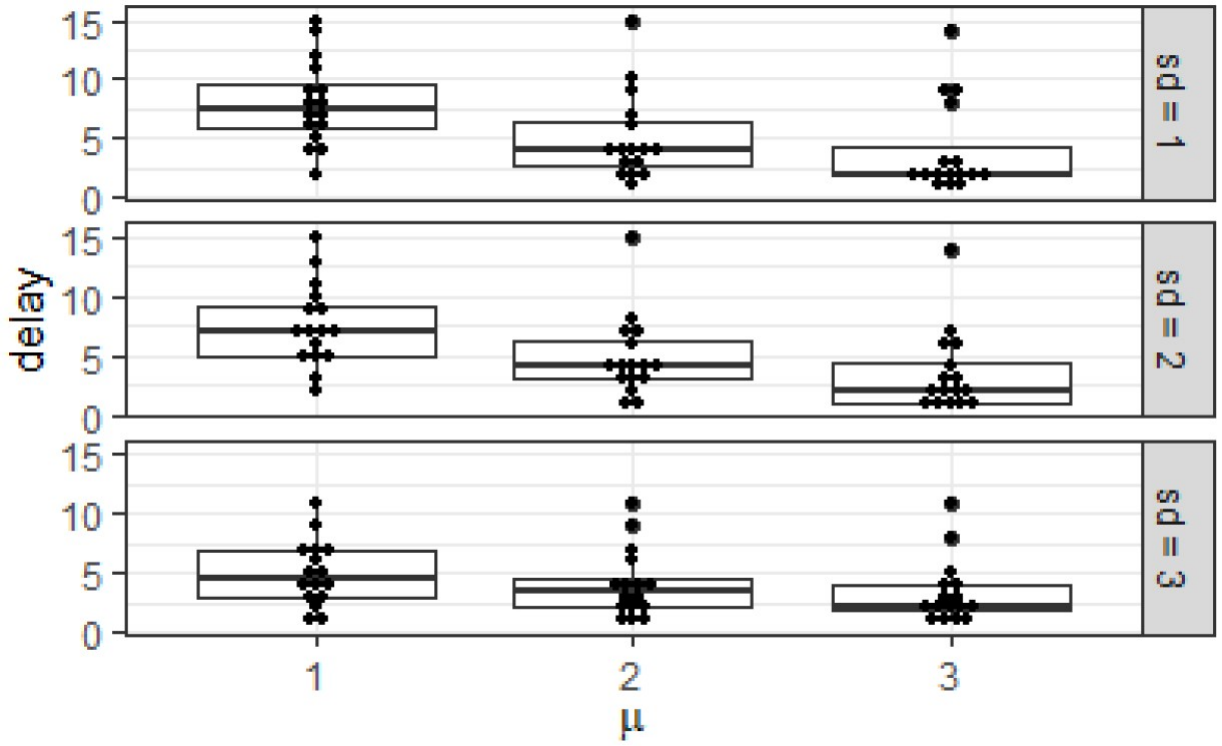
The delay defined as the time difference between the detected start time and actual start time of events for different values of *μ* and *σ* of event starting distribution. The events similar to Fig 19 are generated with different values of *μ* and *σ* and the delay plotted for each combination. We see the median delay increasing with decreasing *μ* and *σ*.

To see the effect of *μ* and *σ* on detection time, we define the delay as the time difference between the detected start time and the actual start time. Fig 20 shows the delays of the 16 events for each combination of *μ* and *σ*. We see that the starting event distribution of N(1,1) has longer delays, which is evidenced by the higher median and range compared to N(3,3). We also see that the delays increase when *μ* and *σ* decrease, i.e. when the events get more feeble.

### 5.3 Comparison of event extraction results

We extract events using CPDBEE and compare with events extracted using Kulldorff’s Scan Statistic. We use the R implementation by Kim and Wakefield [46] to extract events using the Scan Statistic. The scan statistic was originally computed using population counts and the number of patient visits of each geo-spatial region. For our data we analogize the signal value in each cell to the number of patient visits in an epidemiology context. In addition, the formulation needs the population of each cell to compute significant clusters. As we do not have an underlying population for the fibre optic cable, each cell is equally likely to belong to a significant cluster. Therefore we assign the same population value to all cells in our data. We take the maximum signal value of the window multiplied by 20 as the population value of every cell. Thus each cell has a maximum of 5% of population “sick” at a given time. We use a significance level of 5% in our experiments.

Figs 37, 38, 39 and 40 in S1 Appendix contains the complete comparison results for fibre optic, synthetic and NO_2_ data. This section contains only two figures for each dataset due to space constraints.

#### 5.3.1 Events extracted from fibre optic data

**Fig 21:**
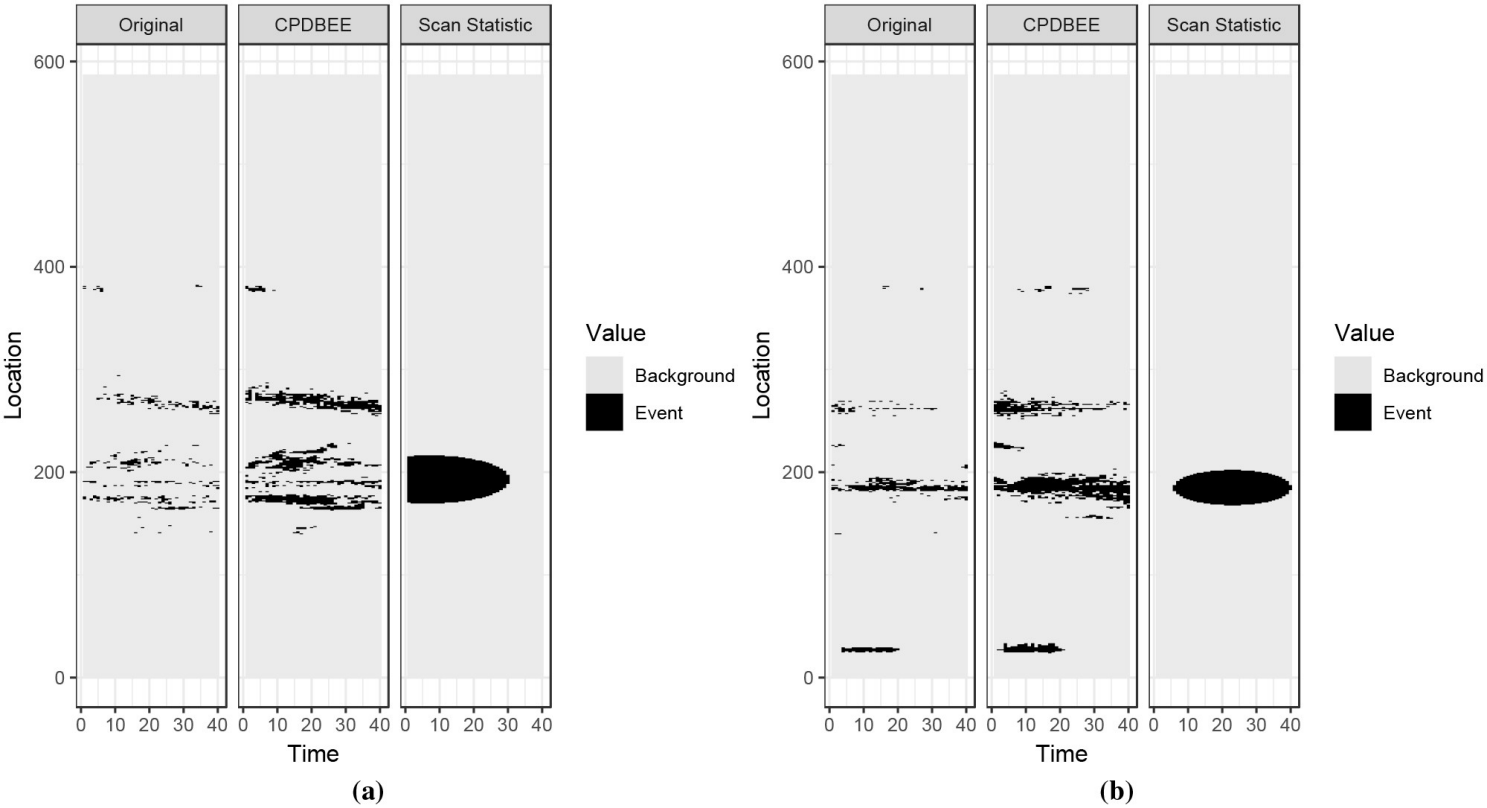
Event extraction comparison for fibre optic data.

Fig 21 shows the fibre optic data and the extracted events using CPDBEE and Kulldorff’s Scan Statistic.

We used a window model and chose a window size of 40 as the Scan Statistic implementation could not handle a larger window size. Even with a window size of 40, Scan Statistic computation took much longer than CPDBEE.

The first tile of each graph shows a simplified version of the original data, i.e. pixels having signal values greater than 40, 000 are depicted in black while other pixels are depicted in grey. Even though the cut-off value of 40, 000 is completely arbitrary, it is purely used for visualisation purposes and is not an input parameter for the event extraction algorithms. The second tile shows the events extracted using CPDBEE and the third tile shows the events extracted using the Scan Statistic algorithm. Pixels belonging to extracted events are depicted in black, while other pixels are depicted in grey.

Class A events are present in the original data in Fig 21b and Figs 37b, 37d, 38c and 38e in S1 Appendix. As discussed previously we do not want to miss Class A events for this particular application. We see that CPDBEE extracts all four class A events, while the Scan Statistic algorithm only extracts the class A event in Fig 37d in S1 Appendix. Furthermore, CPDBEE extracts events in their original shape, while the Scan Statistic algorithm extracts events more in the shape of an ellipse in these examples. As such, CPDBEE is more efficient and effective that the Scan Statistic for this application.

#### 5.3.2 Events extracted from synthetic data

**Fig 22:**
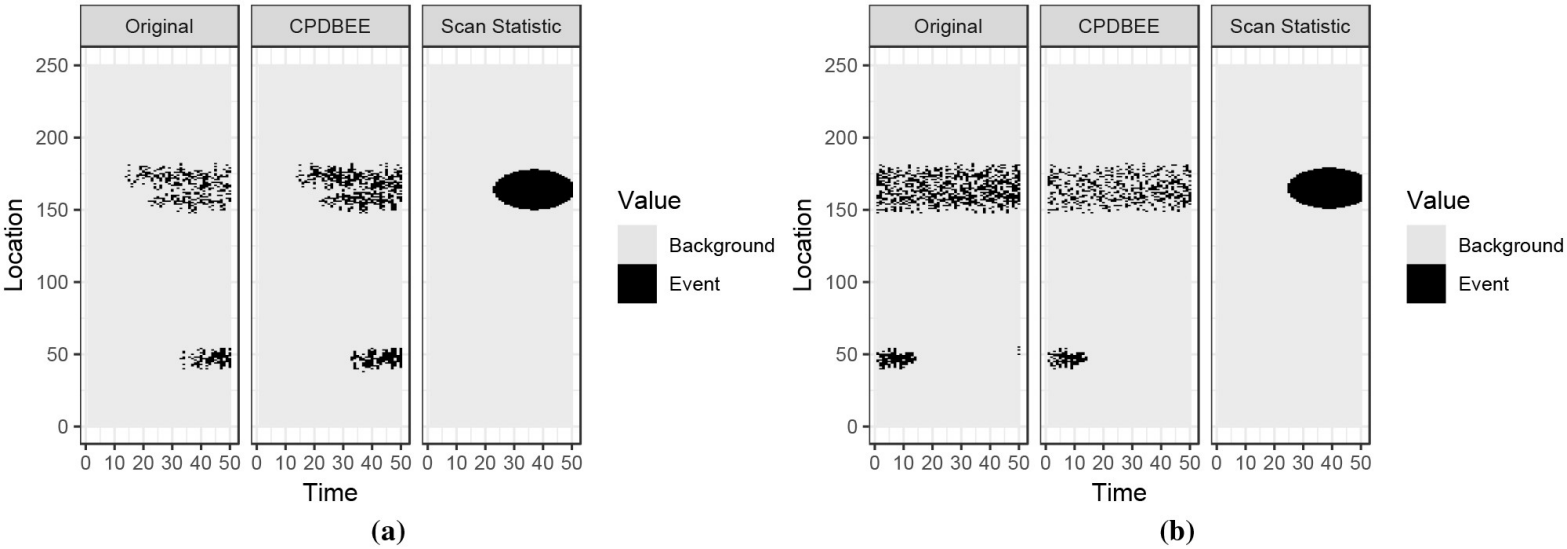
Event extraction comparison for synthetic data.

Using the R package *eventstream*, we generate a 350 × 250 matrix of synthetic data, where 350 denotes the time units and 250 denotes location units. Fig 22 shows two 50 × 250 windows of synthetic data and the events extracted using CPDBEE and Scan Statistic algorithms. The full comparison is illustrated in Fig 39 in S1 Appendix. The choice of the window size is because the Scan Statistic algorithm could not work with bigger window sizes.

The first tile of each graph shows a simplified version of the original window, with pixel values greater than 10 coloured in black and the rest in grey. The second and the third tiles show the events extracted using CPDBEE and Scan Statistic algorithms. Again we see that the events extracted by CPDBEE are more accurate than those extracted using the Scan Statistic algorithm. In addition, the Scan Statistic algorithm misses events in Fig 22a and 22b, Fig 39b, 39c and 39c in S1 Appendix which is detrimental to certain applications.

#### 5.3.3 Events extracted from NO_2_ data

**Fig 23:**
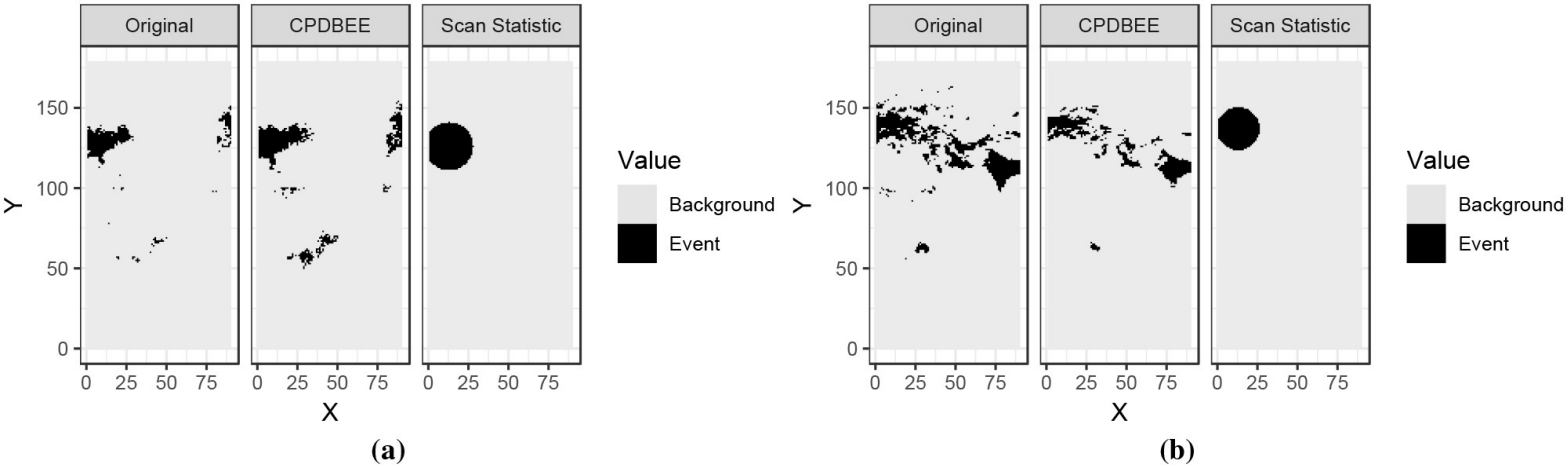
Event extraction comparison for NO_2_ data.

We chose NO_2_ data for March 2018 to evaluate the event extraction algorithms CPDBEE an Scan Statistic. Fig 23 shows the original data and the events extracted by each algorithm for two spatial windows. The first panel shows a simplified version of the original data with NO_2_ values greater than 100 depicted in black and the rest in grey. The second and the third panels show the events extracted using CPDBEE and the Scan Statistic algorithm.

**Fig 24:**
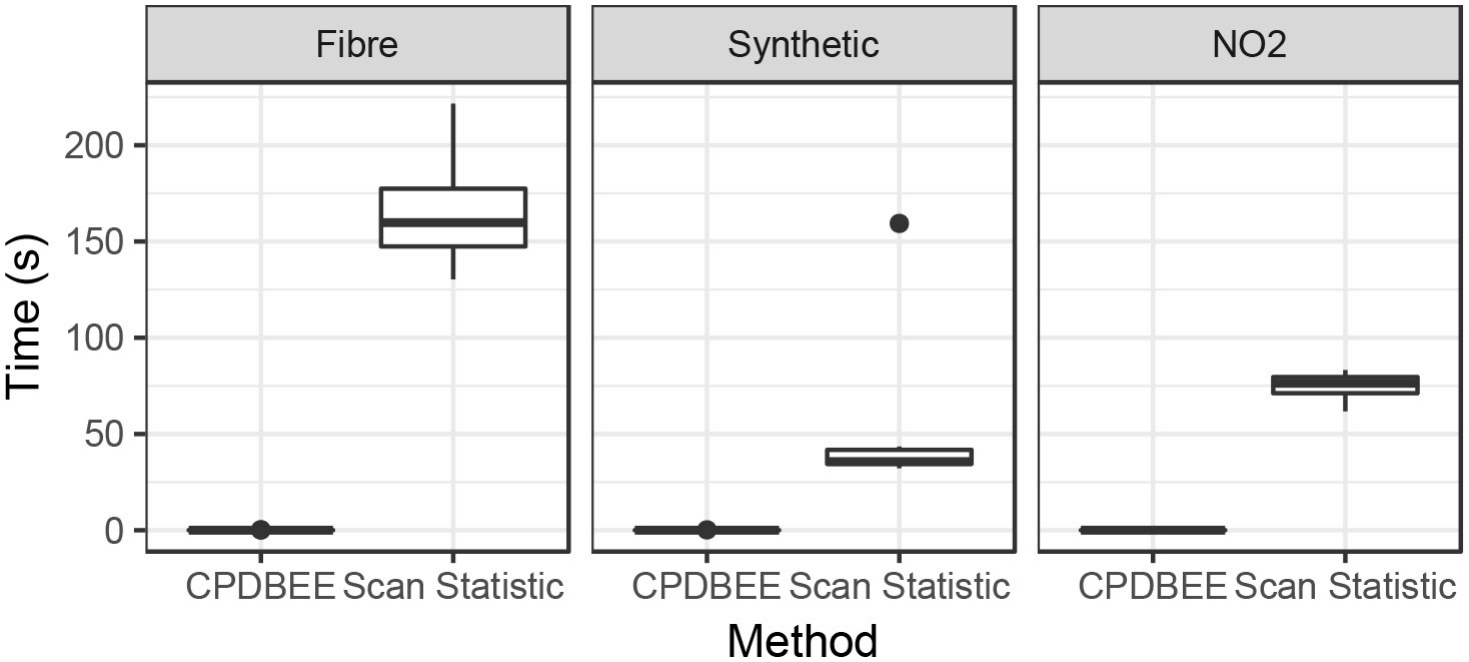
Time comparison of CPDBEE with Scan Statistic for fibre optic data, synthetic data and NO_2_ data.

Fig 24 shows the time taken by CPDBEE and the Scan Statistic algorithm for these three applications using an Intel^®^Core^™^i7-6700, 3.4 GHz processor with 16 GB RAM. We see that CPDBEE extracts events much faster than the Scan Statistic algorithm for all three applications. The Monte Carlo simulations, which is an integral part of the Scan Statistic algorithm contributes to its time intensiveness. Furthermore, CPDBEE does not miss any important events and extracts better shaped events compared to the Scan Statistic algorithm.

## 6 Early event classification framework

### 6.1 Event features

As we work with a data stream, we use a moving window model in our experiments. We extract events from data in the current window and compute features for these events. The feature set comprises some basic features such as length and width of each event, and some other features that compute the intensity of each event relative to the background. The “relative to the background” features are equivalent to a family of signal to noise ratio (SNR) features and are motivated from the fibre optic application (see Fig 4a).

**Fig 25:**
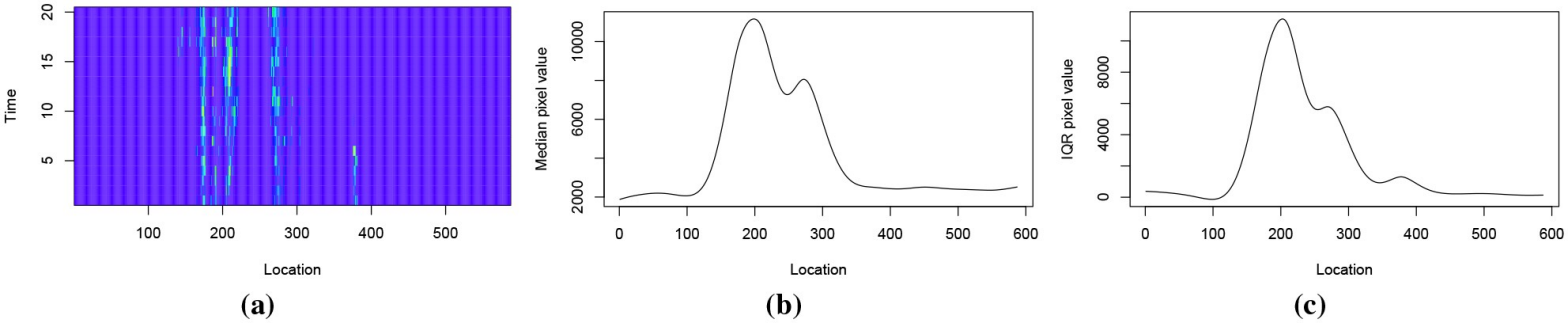
The initial portion of a window and the resulting median and IQR splines.

To compute the SNR family of features we use smoothing splines and thus they are only computed for two-dimensional data streams due to ease of computation. Using a small portion from the beginning of each window, which correspond to the recent past, we compute the mean, median, interquartile range (IQR) and standard deviation for each location. Using these values at each location, we compute four smoothing splines. The objective is to have the background mean, median, IQR and standard deviation pixel value for each location. The median and IQR splines from a small window in Fig 25a are shown in Fig 25b and 25c.

For two-dimensional events we compute the following features:

1. Number of cells/pixels in event
2. Length of event
3. Width of event
4. Length to width ratio of event
5. Centroid
  The centroid is used to compute other features which are relative to the event. It is not used in event classification.
6. Sum of signal-values of cells in event
7. Mean signal-value of event
8. Standard deviation of signal-values of event
9. Slope of the fitted line *ζ*
  The average signal value at each time of the event is computed and a line *ζ* is fitted to the average values. The slope of the fitted line *ζ* is a feature of interest.
10. Linear and quadratic coefficients of a fitted parabola *p*
  The average signal value at each time of the event is computed and a parabola *p* is fitted to the average values. The linear and quadratic coefficients of the fitted parabola *p* are features of interest.
11. *n* standard deviations from the mean
  The proportion of event cells/pixels that has signal-values greater than *n* global standard deviations from the global mean for *n* ∈ {2, 3, 4}.
12. *n* local IQR from local median
  The value of the median smoothing spline at each event centroid is used as the local median for that event. Similarly, the value of the IQR smoothing spline at each event centroid is used as the local IQR for that event. This feature gives the proportion of event pixels/cells that has signal-values greater than *n* local IQRs from the local median for *n* ∈ {5, …, 8}
13. Local IQRs from local median
  Let us denote the 75th percentile of the event signal value by *x*. This feature gives the number of local IQRs for which *x* is greater than the local median. Both local IQR and local median are computed using splines described above.
14. Local standard deviation from local mean
  Similar to the previous feature, our *x* is the 80th percentile of the event signal value. Here we compute the number of local standard deviations for which *x* is greater than the local mean.

For three-dimensional data streams we compute a subset of the above features. In particular, we compute features 1–10 from the above list and an equivalent of feature 14 using the global standard deviation and the global mean. In addition, we use the squared value of these features in applications with enough data, i.e. the synthetic and NO_2_ datasets. These features now provide a compact way to represent a data stream and the embedded events, summarising salient properties of the time window in terms of event signal strength and shape. This summary becomes input to a classifier to identify types of events.

### 6.2 Partial/incomplete observations

In the classical setting, a classification problem comprises observations (*x*_*i*_, *y*_*i*_) for *i* ∈ 1, …, *N* where $x_{i}\in R^{b}$ is the attribute vector of the *i*th observation and *y*_*i*_ is its class label. The task of the classifier is to learn the class boundary by using the given set of observations. Then for any new observation *x*_*j*_, the classifier can predict its class label using the learned class boundaries. Let us call this a **standard classifier**.

Standard classifiers have been widely popular in diverse fields of study and practice. However, they are not without limitations. One of the limitations is that once a classifier is trained, it has fixed class boundaries. If the new data is different from the data learned by the classifier, the output of the classifier is of little use. This is particularly the case in data-streaming scenarios, where data distributions are non-stationary (sometimes also referred to as concept drift). It is necessary for a classifier to re-adjust its class boundaries when faced with non-stationarity. The literature on adapting or evolving classifiers is significant [47]. Let us call these classifiers **evolving classifiers**.

Now, consider the case when a new observation is not made available at once but gradually, where we get partial information about the new observation and the amount of partial information increases with time. This is the case for events described in Section 1.1. Let *x*_*j*_ be a new observation which becomes available partially via the following finite sequence of partial observations $\left\{p_{t_{1}}^{j},p_{t_{2}}^{j},p_{t_{3}}^{j},\ldots ,p_{t_{n}}^{j}\right\}$. Here the partial observation of *x*_*j*_ at age *t*_*k*_ is denoted by $p_{t_{k}}^{j}$ and $p_{t_{n}}^{j}=x_{j}$ with *t*_1_ < *t*_2_ < ⋯ < *t*_*n*_. We differentiate between the time and the age of a partial observation. A partial observation that begins at time *t* = *t*_1_ has age 0 at time *t*_1_, and at time *t* = *t*_2_ it has age *t*_2_ − *t*_1_.

We consider the question “how can we classify partial observations?” If one trains a single standard classifier on all partial observations, it may be optimal for a certain set of partial observations $p_{t_{k}}$ at a given age *t*_*k*_, but not all partial observations, because partial observations change with time. If one waits until the partial observation has formed into a full observation *x*_*j*_, then a standard classifier can be used. However, for some applications such as intrusion detection it might be too late to wait until the full observation has formed. One option is to have a series of standard classifiers ${\left\{C_{t_{i}}\right\}}_{i=1}^{n}$ each trained on partial observations $p_{t_{i}}$. When a new observation gradually arrives in the form of a sequence of partial observations $\left\{p_{t_{1}}^{k},p_{t_{2}}^{k},p_{t_{3}}^{k},\ldots ,p_{t_{n}}^{k}\right\}$, the classifier $C_{t_{i}}$ can be used on $p_{t_{i}}^{k}$. Thus, as the partial observation grows, we have a growing prediction $\left\{y_{t_{1}},y_{t_{2}},\ldots ,y_{t_{n}}\right\}$ of the class label. More importantly, we do not need to wait until the partial observation matures to a full observation before making a prediction.

However, having a series of classifiers independent of each other is sub-optimal because each classifier is only trained on a portion of the data, i.e. it is trained on individual snapshots of events at different ages. By linking event snapshots of different event-ages in an appropriate way, better predictions can be achieved.

In addition, event extraction algorithms may miss events of interest when the events are very young. As such there may be more events extracted at age *t*_2_ compared to age *t*_1_. Similarly, all events may not continue until age *t*_*n*_. Consequently there may be more events at age *t*_*n*−1_ compared to *t*_*n*_. For example consider 20 events which are extracted at ages {*t*_1_, *t*_2_, *t*_3_, *t*_4_, *t*_5_} such that only 5 are extracted at age *t*_1_, 15 at *t*_2_, 20 at *t*_3_, 15 at *t*_4_ and 5 at *t*_5_. In such a scenario, if we have a set of 5 independent classifiers $\left\{C_{t_{1}},C_{t_{2}},C_{t_{3}},C_{t_{4}},C_{t_{5}}\right\}$, $C_{t_{1}}$ and $C_{t_{5}}$ have only a quarter of the observations for training. In contrast, a classifier that links all partial event observations has access to a bigger pool of training data.

Furthermore, classifying young events is generally harder than classifying matured events because often there is no clear separability of classes when events are young. If we continue with the previous example, obtaining the correct class boundary at *t*_1_ is harder than at *t*_2_, making it difficult for $C_{t_{1}}$ to independently ascertain the class boundary. However, a linked classifier which sees all partial event observations can come up with a realistic class boundary for age *t*_1_ because it sees the partial observations at ages *t*_2_, *t*_3_ and *t*_4_, which helps it to form the class boundary at *t*_1_. Thus, we expect linking partial event observations at different ages to aid early classification.

The Connected Classifier CC described in the next section links partial event observations of all ages to give a growing prediction.

### 6.3 CC: Connected classifier

**Fig 26:**
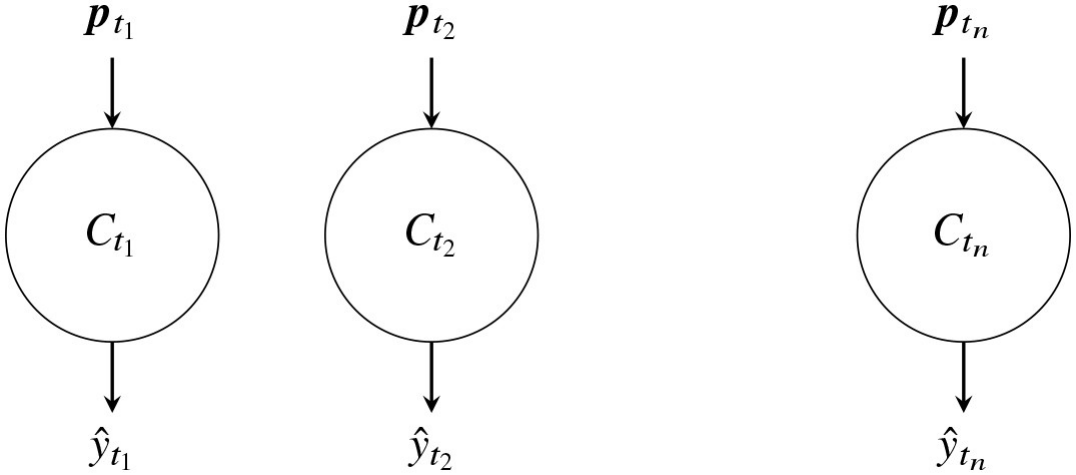
n independent classifiers.

Let the standard classifier minimize the loss function given by L, i.e.

$$\begin{array}{c} \text{arg}\;\underset{\beta }{\text{min}}\frac{1}{N}\sum_{i=1}^{N}L\left(x_{i},y_{i};\beta \right), \end{array}$$

where *β* = (*β*_0_, *β*_1_, …, *β*_*l*_) and (*x*_*i*_, *y*_*i*_) are observations for *i* ∈ {1, …, *N*}. Now consider a set of independent classifiers ${\left\{C_{t_{j}}\right\}}_{j=1}^{n}$ each trained and tested on partial observations of age *t*_*j*_ as denoted in Fig 26.

Each $C_{t_{j}}$ minimizes the loss function

$$\begin{array}{c} \frac{1}{N}\sum_{i=1}^{N}L\left(p_{t_{j}}^{i},y_{i};{\tilde{\beta }}_{j}\right), \end{array}$$

with ${\tilde{\beta }}_{j}={\left({\tilde{\beta }}_{j0},{\tilde{\beta }}_{j1},{\tilde{\beta }}_{j2},\ldots ,{\tilde{\beta }}_{jl}\right)}^{T}$. If we stack ${\tilde{\beta }}_{j}$ in rows we get a resulting matrix $\tilde{\beta }$ such that

$$\begin{array}{c} \tilde{\beta }=\left\{{\tilde{\beta }}_{jk}\right\}=[{\tilde{\beta }}_{1},{\tilde{\beta }}_{2},\ldots ,{\tilde{\beta }}_{n}{]}^{T}. \end{array}$$

The matrix $\tilde{\beta }$ can also be obtained by minimizing the loss function

$$\begin{array}{c} \frac{1}{nN}\sum_{j=1}^{n}\sum_{i=1}^{N}L\left(p_{t_{j}}^{i},y_{i};\tilde{\beta }\right), \end{array}$$ (5)

as ${\left\{p_{t_{j}}^{i}\right\}}_{i=1}^{N}$ for a fixed age *t*_*j*_ only affects ${\tilde{\beta }}_{j}$. Therefore the matrix $\tilde{\beta }$ can be computed row by row by minimizing $\sum_{i=1}^{N}L(p_{t_{j}}^{i},y_{i};{\tilde{\beta }}_{j})$ for each *j*. Thus, we can write

$$\begin{array}{c} \underset{\tilde{\beta }}{\text{arg}\;\text{min}}\sum_{j=1}^{n}\sum_{i=1}^{N}L\left(p_{t_{j}}^{i},y_{i};\tilde{\beta }\right)=[\underset{{\tilde{\beta }}_{1}}{\text{arg}\;\text{min}}\sum_{i=1}^{N}L\left(p_{t_{1}}^{i},y_{i};{\tilde{\beta }}_{1}\right),\ldots ,\underset{{\tilde{\beta }}_{n}}{\text{arg}\;\text{min}}\sum_{i=1}^{N}L\left(p_{t_{n}}^{i},y_{i};{\tilde{\beta }}_{n}\right){]}^{T}\;\;. \end{array}$$ (6)

Thus, *n* independent classifiers ${\left\{C_{t_{j}}\right\}}_{j=1}^{n}$ minimize the loss function given in Eq (5).

Having *n* independent classifiers ${\left\{C_{t_{j}}\right\}}_{j=1}^{n}$ is sub-optimal because the partial observations at ages *t*_*j*_ are not independent from those aged *t*_*j*−1_ and *t*_*j*+1_. Thus the classifier $C_{t_{j}}$ can benefit from the knowledge of $C_{t_{j+1}}$ and vice-versa. Furthermore, the partial observations of an event change little from *t*_*j*_ to *t*_*j*+1_. Taking these into account, we modify the original loss function given in Eq (5) by including an *L*_2_ penalty term as follows:

$$\begin{array}{c} \varphi \left(\tilde{\beta },\lambda \right)=\frac{1}{nN}\sum_{j=1}^{n}\sum_{i=1}^{N}L\left(p_{t_{j}}^{i},y_{i};\tilde{\beta }\right)+\lambda \sum_{j=1}^{n-1}\|{\tilde{\beta }}_{j+1}-{\tilde{\beta }}_{j}{\|}^{2} \end{array}$$ (7)

for some λ > 0, where ‖⋅‖ denotes the *L*_2_ norm. The constant λ is a parameter that can be specified. Recall that ${\tilde{\beta }}_{j}=({\tilde{\beta }}_{j0},{\tilde{\beta }}_{j1},{\tilde{\beta }}_{j2},\ldots ,{\tilde{\beta }}_{jl})$ relates to partial observations ${\left\{p_{t_{j}}^{i}\right\}}_{i=1}^{N}$ for a fixed *t*_*j*_, i.e. ${\tilde{\beta }}_{j0}$ is the coefficient of the intercept at age *t*_*j*_ and ${\tilde{\beta }}_{j1}$ is the coefficient of the first covariate at age *t*_*j*_. Thus the penalty term

$$\begin{array}{c} \|{\tilde{\beta }}_{j+1}-{\tilde{\beta }}_{j}{\|}^{2}=\sum_{k=0}^{l}{\left({\tilde{\beta }}_{j+1,k}-{\tilde{\beta }}_{j,k}\right)}^{2}, \end{array}$$

and each term ${\left({\tilde{\beta }}_{j+1,k}-{\tilde{\beta }}_{j,k}\right)}^{2}$ takes coefficients for the *k*th covariate at ages *t*_*j*_ and *t*_*j*+1_ and penalizes the difference, enforcing a certain smoothness in event-age. The connected classifier CC minimizes this loss function. As a result of the *L*_2_ penalty term, the individual classifiers are connected to form a single classifier. Thus CC finds ${\tilde{\beta }}_{*}$ such that,

$$\begin{array}{c} {\tilde{\beta }}_{*}=\underset{\tilde{\beta }}{\text{arg}\;\text{min}}(\frac{1}{nN}\sum_{i=1}^{N}\sum_{j=1}^{n}L\left(p_{t_{j}}^{i},y_{i};\tilde{\beta }\right)+\lambda \sum_{j=1}^{n-1}\|{\tilde{\beta }}_{j+1}-{\tilde{\beta }}_{j}{\|}^{2}). \end{array}$$ (8)

**Fig 27:**
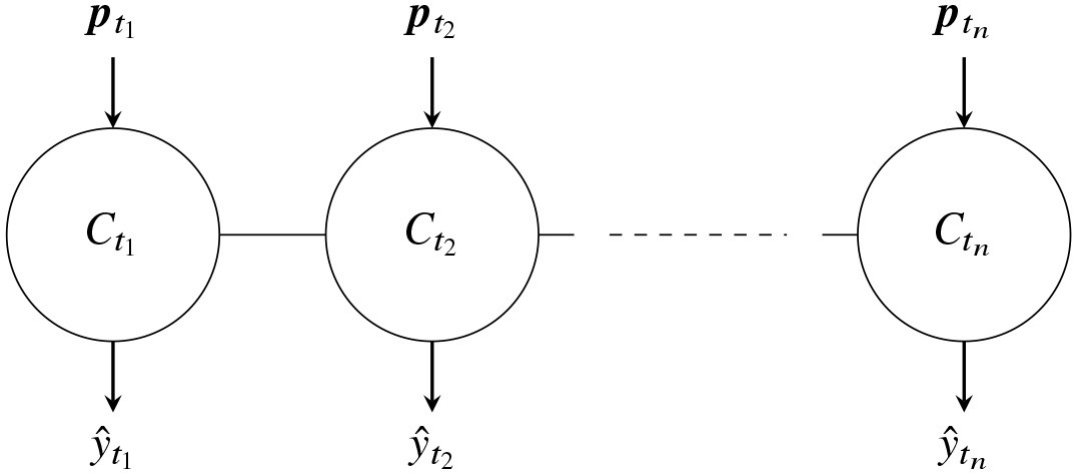
A connected classifier.

When λ = 0, CC is equivalent to *n* independent classifiers as the cost function is reduced to that of Eq (5). When λ → ∞ the coefficients ${\tilde{\beta }}_{j+1}\to {\tilde{\beta }}_{j}$ to minimize the cost function. We recall that ${\tilde{\beta }}_{j}$ is the vector of coefficients of $C_{t_{j}}$. This gives rise to the same classifier for all event ages. Thus, CC is a connected classifier that is in between a single classifier and *n* independent classifiers. The parameter λ controls how close or far away CC is from *n* independent classifiers or a single classifier. Values of λ close to zero makes the connected classifier closer to *n* independent classifiers. Large values of λ makes CC closer to a single classifier, with little difference in the weights ${\tilde{\beta }}_{j}$ for different *j*. A schematic diagram of CC is shown in Fig 27. We can think of λ as controlling the strength of the connections between classifiers $C_{t_{j}}$ and $C_{t_{j+1}}$. Large values of λ results in stronger connections between $C_{t_{j}}$ and $C_{t_{j+1}}$ making them more alike. Small values of λ makes weaker connections between $C_{t_{j}}$ and $C_{t_{j+1}}$ giving them more freedom to choose their own weights. No single value of λ is suitable for all problems. The optimal value of λ depends on how fast the partial observations $p_{t_{j}}^{i}$ change with age *t*_*j*_. Thus, it depends on the ages *t*_*j*_. If the gap between the ages *t*_*j*_, and *t*_*j*+1_ is small then $C_{t_{j}}$ is more likely to be similar to $C_{t_{j+1}}$. This can be achieved by a larger λ. If the gap between successive ages is bigger, then $C_{t_{j}}$ may be quite different from $C_{t_{j+1}}$, which can be achieved by a smaller λ.

As any loss function L can be used in Eq (8), CC is a general formulation. Our implementation of CC in *eventstream* [14] uses logistic regression as the base classifier and the associated loss function. However, CC can be implemented with black other loss functions such as those employed in decision trees, neural nets, or SVMs. The connected classifier can be thought of as a blue print or framework for classifying developing events while they are still premature, without locking in a base classifier.

For logistic regression [48] the loss function L is given by

$$\begin{array}{c} L\left(p_{t_{j}}^{i},y_{i};\tilde{\beta }\right)=-y_{i}(\left[1\;\;{\left(p_{t_{j}}^{i}\right)}^{T}\right]{\tilde{\beta }}_{j})+\text{log}(1+\text{exp}\{\left[1\;\;{\left(p_{t_{j}}^{i}\right)}^{T}\right]{\tilde{\beta }}_{j}\}). \end{array}$$ (9)

Here the vector $\left[1\;\;{\left(p_{t_{j}}^{i}\right)}^{T}\right]$ denotes the concatenation of the vector $p_{t_{j}}^{i}$ with the constant 1 to account for the intercept.

We use gradient based optimization procedures to minimize the loss function in Eq (7). As such, the training complexity of the logistic connected classifier depends on the following: 1. the number of features or attributes *l*, 2. the number of distinct ages, i.e. for *t*_*i*_ with *i* ∈ {1, …, *n*} we have *n* ages, 3. the number of training observations *N* and 4. the number of iterations or epochs *e* in the optimization process. Therefore, the training complexity of the logistic connected classifier is O((l+1)nNe). Here we have (*l* + 1) to account for the intercept. Once the connected classifier is trained the testing complexity of $p_{t_{j}}^{i}$ for a fixed *i* and *j* is simply O(l+1) as it is a multiplication of the weights.

### 6.4 An example

**Fig 28:**
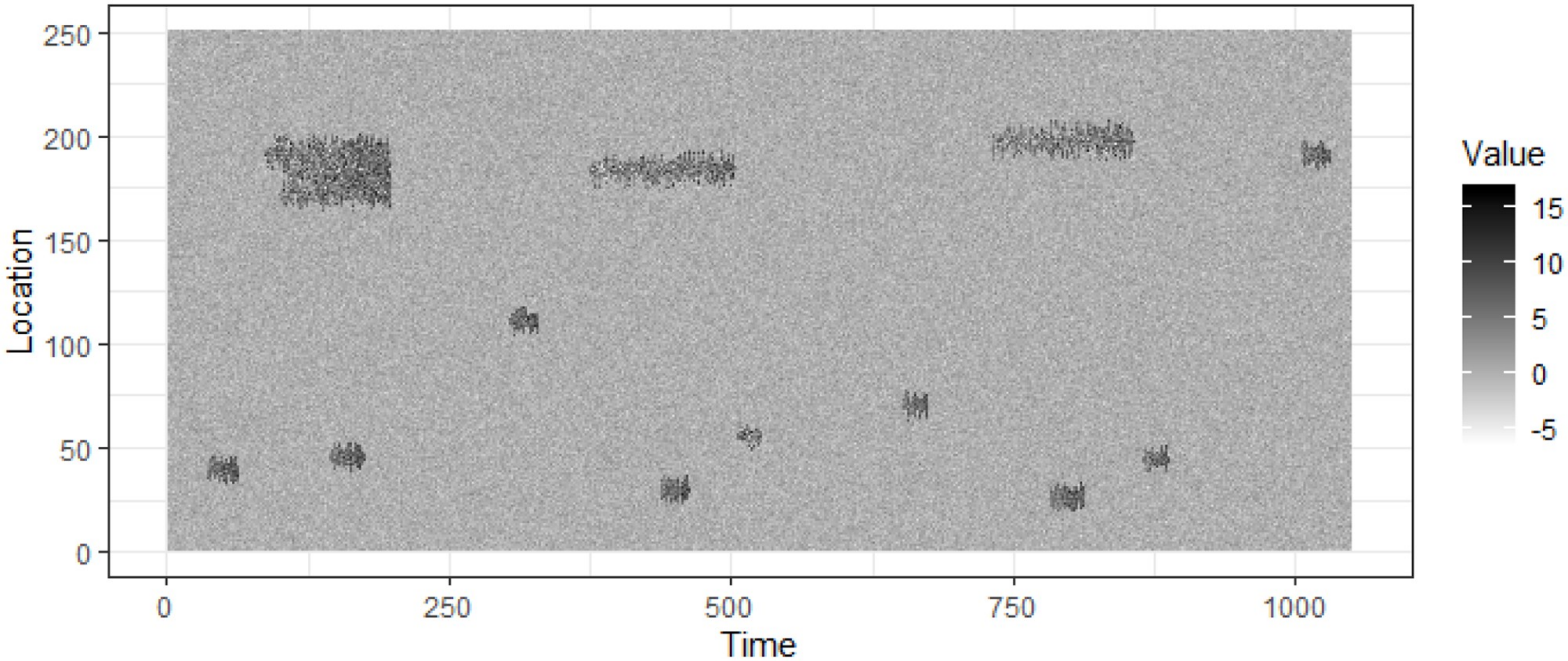
A synthetic data stream with 12 events.

In this section we explore the effect of the parameter λ on the cost function $\varphi (\tilde{\beta },\lambda )$ of Eq (7). We consider the synthetic data shown in Fig 28 and extract events using a moving window model with a window size 200 and step size 8.

Using a test set we compute the cost function for different values of λ ranging from 1 to 40. We record the cost due to the *n* independent classifiers and the penalty term separately as follows:

$$\begin{array}{c} C_{1}=\frac{1}{nN}\sum_{i=1}^{N}\sum_{j=1}^{n}L\left(p_{t_{j}}^{i},y_{i};\tilde{\beta }\right)\;,\;C_{2}=\lambda \sum_{j=1}^{n-1}\|{\tilde{\beta }}_{j+1}-{\tilde{\beta }}_{j}{\|}^{2}\;. \end{array}$$

**Fig 29:**
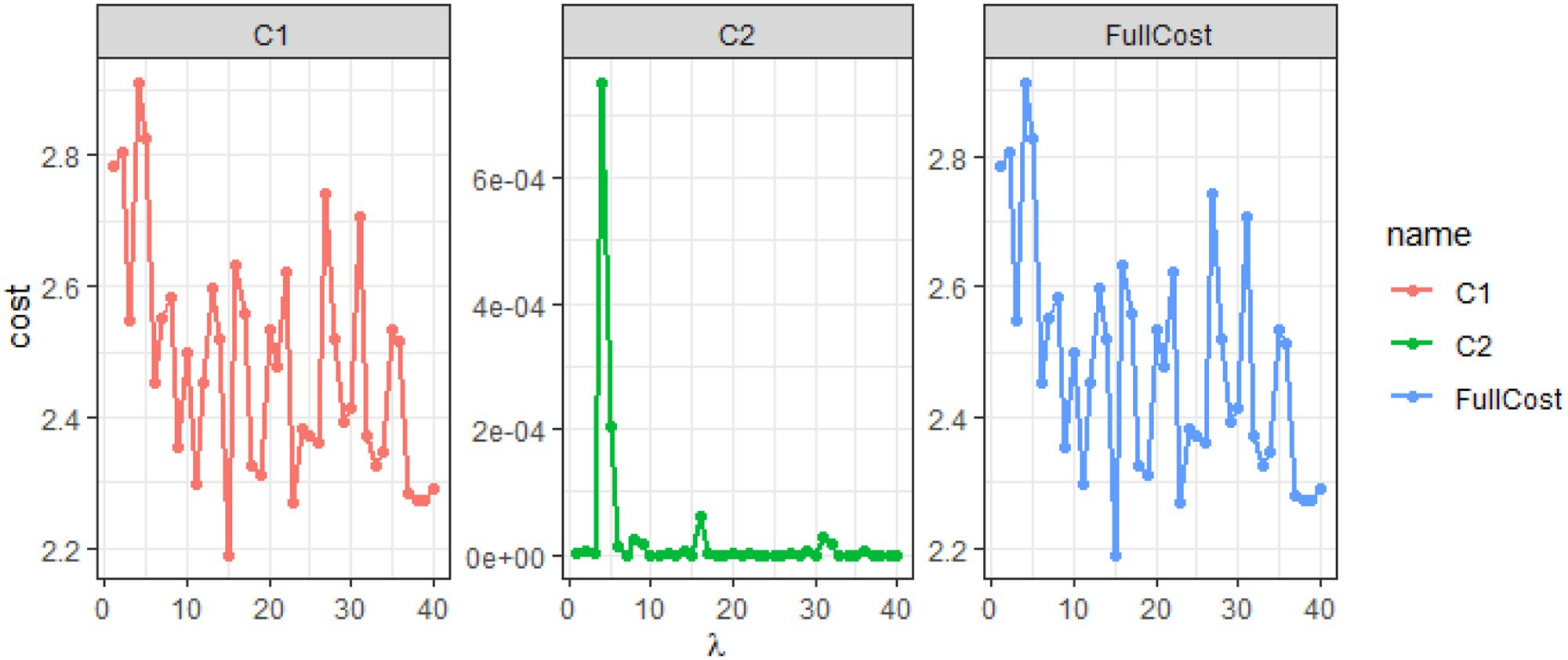
The costs *C*_1_, *C*_2_ and the total cost with λ varying from 1 to 40.

Fig 29 shows the costs *C*_1_, *C*_2_ and the total cost for this exercise. The cost *C*_2_ is quite low compared to *C*_1_ for this example. We see that the total cost has a negative trend initially, i.e. it decreases with increasing λ. Even though *C*_2_ is much smaller compared to *C*_1_, having the penalty term gives rise to better $\tilde{\beta }$ that reduces the total cost. A test or validation set needs to be considered in choosing λ to avoid over-fitting.

### 6.5 Comparison with unlinked classifiers

**Fig 30:**
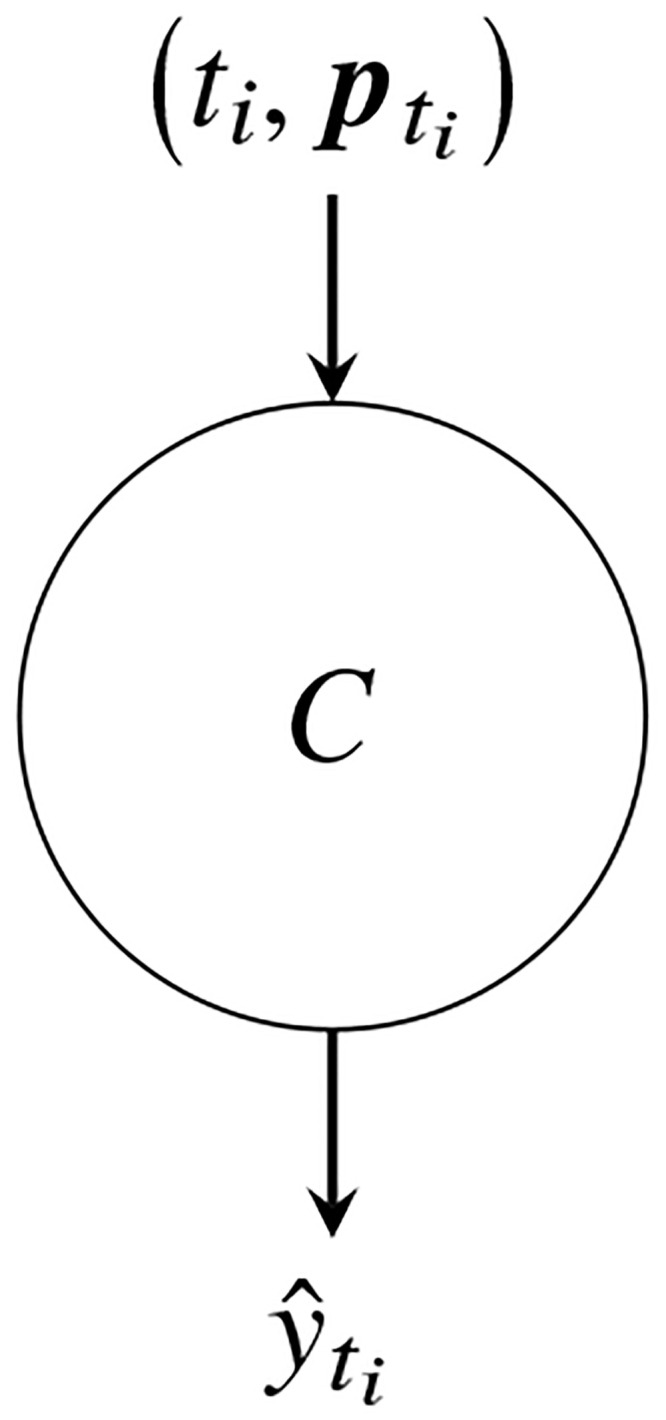
A single classifier.

We refer to CC with logistic regression as CC-Log in the following sections and compare its performance with two configurations of logistic regression classifiers. The first configuration comprises a single classifier, which is trained on all partial observations and their ages $\left(t_{i},{\text{p}}_{t_{i}}\right)$ as shown in Fig 30. We refer to this configuration as 1-Log in the following sections. The second configuration comprises *n* independent classifiers as shown in Fig 26. We refer to this configuration of *n* independent classifiers as *n*-Log. The *n*-Log classifier comprises of ${\left\{C_{t_{j}}\right\}}_{j=1}^{n}$, where each $C_{t_{j}}$ is trained on partial observations of age *t*_*j*_, i.e. ${\left\{p_{t_{j}}^{i}\right\}}_{i=1}^{N}$.

The difference between 1-Log and *n*-Log is the number of independent classifiers. The only difference between *n*-Log and CC-Log is the connections between the independent classifiers, brought about by the *L*_2_ penalty term $\sum_{j=1}^{n-1}\|{\tilde{\beta }}_{j+1}-{\tilde{\beta }}_{j}{\|}^{2}$. We compare CC-Log with 1-Log and *n*-Log because we want to understand whether linking independent classifiers benefits early classification.

## 7 Event classification results

We explore three groups of datasets in this Section: synthetic, fibre optic and NO_2_ data. For each application, we use CC-Log, 1-Log and *n*-Log to classify events extracted by CPDBEE algorithm. For all three applications we use λ = 0.05 for consistency. The R code applicable to this section is available in the S1 File. The data is either included in the R package *eventstream* or can be generated using its functionality.

### 7.1 Synthetic data

**Fig 31:**
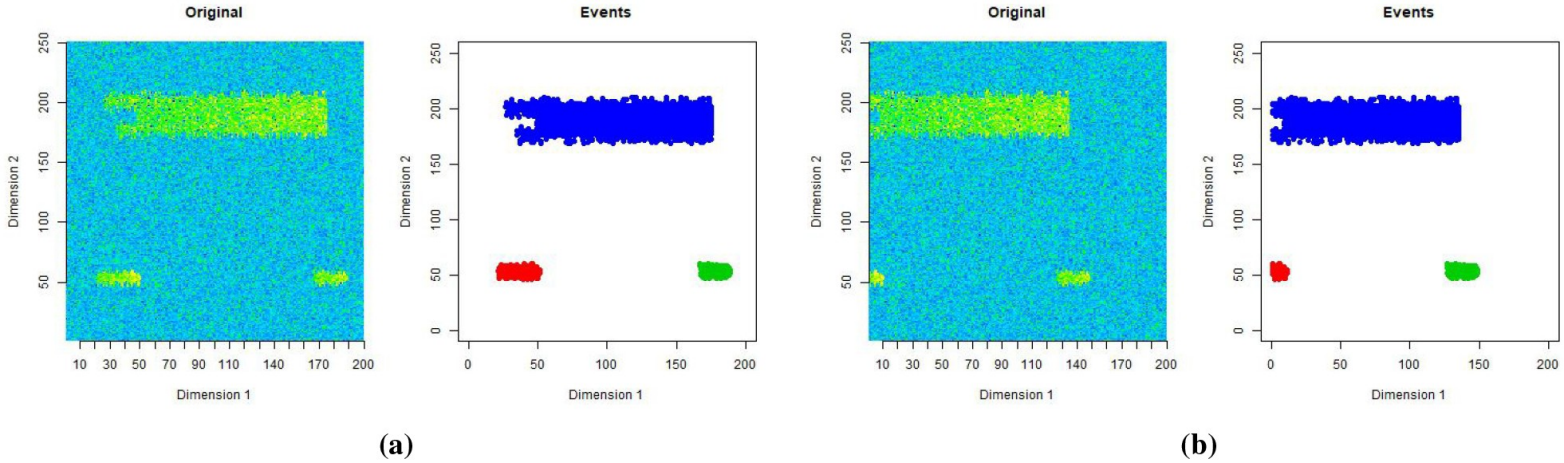
Two windows of data and extracted events.

We generate a data stream of dimension 3500 × 250, of which 80% (2800 × 250) is used for training and the remaining 20% for testing. We use a moving window of dimension 200 × 250 which moves by a step of 8 × 250. For each window we extract events using CPDBEE algorithm as shown in Fig 31. As class A events can have a maximum age of 30, we use 4 event ages for the classification tasks at *t* = 8, 16, 24 and 32 time units, i.e. event features are calculated at these ages. For synthetic data classification, we do not use features which were motivated from the fibre optic example, i.e. we do not use features 11 and 12 from the list in Section 6.1, for computational efficiency. Furthermore, the centroid is not used in any classification task.

To obtain unbiased estimates, we repeat this experiment 5 times using different seeds for data generation. We measure the classification accuracy, which is defined as 1− misclassification error. Table 3 gives the mean and standard deviation of test set classification accuracy for the 3 classifiers, which shows that CC-Log surpasses 1-Log and *n*-Log classifiers. Also we see that all 3 classifiers improve their average accuracy levels with the age of the events with the exception of *n*-Log at *t*_4_.

**Table 3:** Mean and standard deviation of classification accuracy over 5 repetitions.

Accuracy Measure	Classifier	Accuracy				Standard deviation			
		*t*_1_	*t*_2_	*t*_3_	*t*_4_	*t*_1_	*t*_2_	*t*_3_	*t*_4_
Classification Accuracy	CC-Log	0.79	0.88	0.91	0.91	0.13	0.11	0.10	0.08
	1-Log	0.71	0.85	0.88	0.87	0.20	0.13	0.13	0.11
	*n*-Log	0.76	0.84	0.89	0.51	0.12	0.11	0.07	0.28

#### 7.1.1 Significance results

To determine if there is a significant difference between the three classifiers, we conduct a Friedman test on classification accuracy results. The Friedman test on classification accuracy results gave a *p*-value of 0.0156, showing that the classifiers are different at 5% level of significance. To ascertain which methods perform better we conduct a Nemenyi test on classification accuracy results.

**Fig 32:**
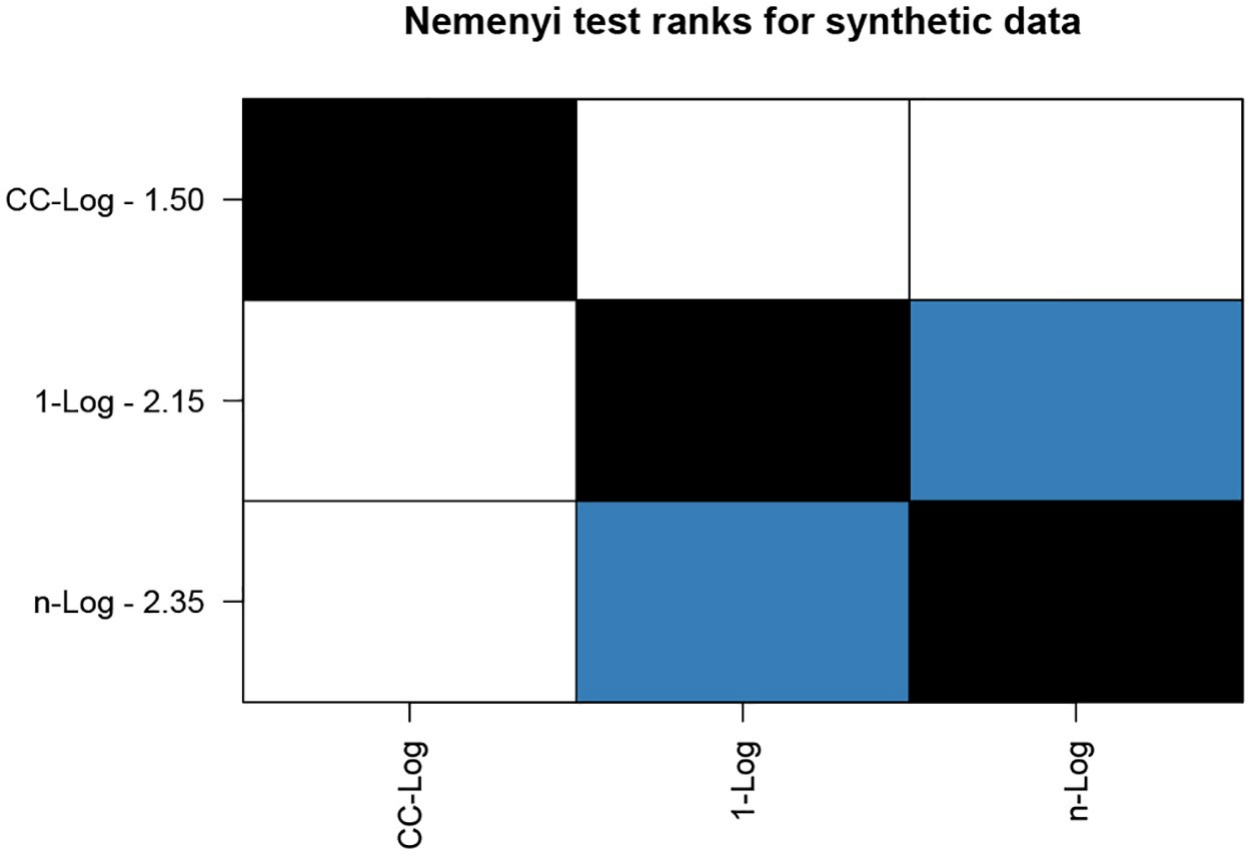
Nemenyi plot for synthetic data using classification accuracy results.

Fig 32 shows the resulting Nemenyi plot of ranks with a 90% level of confidence. Lower rank values indicate better performing methods. Blue coloured boxes indicate methods which do not differ significantly from each other. From Fig 32 we see that CC-Log is best suited for this data followed by 1-Log and *n*-Log, with no significant difference between the two latter methods.

### 7.2 Fibre optic cable data results

The fibre optic dataset is a 379 × 587 matrix as shown in Fig 7. We use a moving window model with a window size 40 × 587 and a step size 10 × 587 to extract events and compute features. For each window we extract events using CPDBEE and use CC-Log, 1-Log and *n*-Log to classify them. We use event ages *t* = 10, 20, 30 and 40, because the maximum event-age is 40 time units.

**Fig 33:**
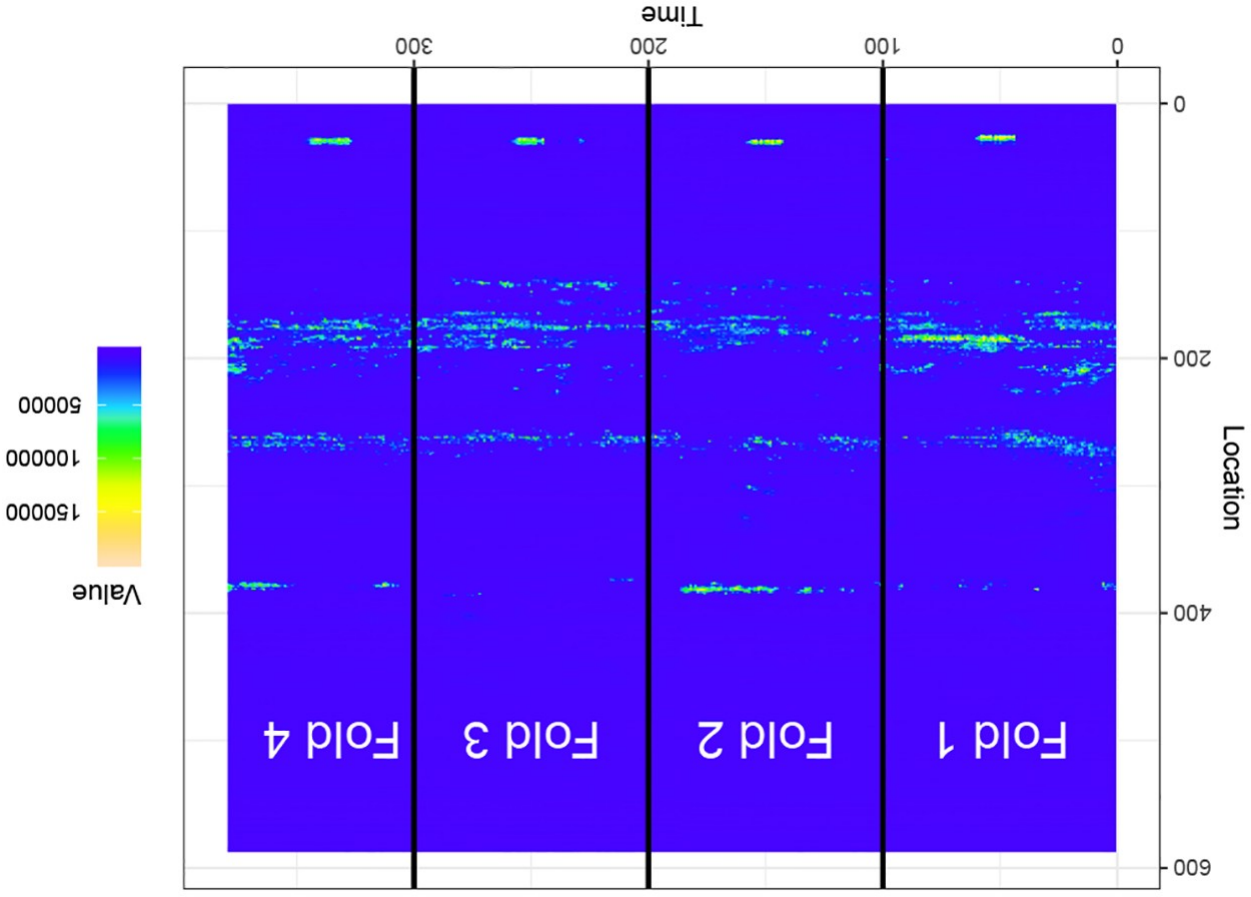
Data chunks for 4-fold cross validation.

As the fibre optic dataset has 4 class A events, we use 4-fold cross validation. The events extracted from the data stream is divided into four folds with each fold containing one class A event resembling Fig 33.

This modified form of cross validation is commonly used for time dependent observations [49] as in a streaming data scenario. The classifiers CC-Log, 1-Log and *n*-Log are trained on events comprising of 3 training folds, and tested on the remaining fold.

As this dataset has a smaller number of class A events compared to class B events, we report additional accuracy measures that are designed for imbalanced datasets. We compute the positive predictive value (PPV) and the negative predictive value (NPV) and area under the receiver operator characteristic curve (AUC). We give the definitions of these metrics below:

$$\begin{array}{c} \text{Positive}\;\text{predictive}\;\text{value}\;\text{(PPV)}=\frac{\text{Number}\;\text{of}\;\text{true}\;\text{positives}}{\text{Number}\;\text{of}\;\text{predicted}\;\text{positives}}, \end{array}$$

$$\begin{array}{c} \text{Negative}\;\text{predictive}\;\text{value}\;\text{(NPV)}=\frac{\text{Number}\;\text{of}\;\text{true}\;\text{negatives}}{\text{Number}\;\text{of}\;\text{predicted}\;\text{negatives}}. \end{array}$$

The number of predicted positives in PPV is the sum of true positives and false positives, and the number of predicted negatives in NPV is the sum of true negatives and the false negatives. Considering PPV and NPV together gives a two-sided accuracy measure. For example, a classifier that predicts all observations as negative except for one correct positive observation achieves a PPV of 100% but a small NPV. The combination of PPV and NPV gives the overall accuracy of the model.

In contrast, AUC is a single measure that captures the effectiveness of a classifier. The receiver operator characteristic (ROC) curve is a plot of the true positive rate against the false positive rate for different classification thresholds. The area under the curve (AUC) provides a measure of discrimination between positive and negative classes. The AUC does not depend on the classification threshold as it is an aggregate measure. An AUC closer to 1 is reflective of a good model, while a random predictor will give an AUC closer to 0.5. The AUC can be interpreted as the probability that a positive observation is ranked higher than a negative observation.

Table 4 gives the average PPV, NPV and AUC values with their standard deviations over the 4-folds for the fibre-optic data stream. For PPV and NPV, we use a probability threshold of 0.5; i.e. if the output probability is greater than 0.5, it is deemed class A, and class B otherwise.

**Table 4:** Mean and standard deviation of PPV, NPV and AUC (%) over 4 folds.

Accuracy Measure	Classifier	Mean				Standard deviation			
		*t*_1_	*t*_2_	*t*_3_	*t*_4_	*t*_1_	*t*_2_	*t*_3_	*t*_4_
PPV	CC-Log	1.00	1.00	1.00	0.95	0.0	0.0	0.00	0.1
	1-Log	0.81	0.73	0.73	0.73	0.21	0.32	0.32	0.32
	*n*-Log	0.90	1.00	0.93	1.00	0.20	0.0	0.12	0.0
NPV	CC-Log	0.92	0.94	0.95	0.95	0.03	0.03	0.03	0.03
	1-Log	0.95	0.96	0.95	0.93	0.01	0.02	0.05	0.06
	*n*-Log	0.97	0.92	0.96	0.96	0.02	0.03	0.02	0.03
AUC	CC-Log	0.96	0.97	0.97	0.95	0.01	0.01	0.01	0.06
	1-Log	0.88	0.84	0.84	0.83	0.09	0.15	0.15	0.13
	*n*-Log	0.93	0.96	0.94	0.98	0.09	0.01	0.05	0.01

#### 7.2.1 Significance results

**Fig 34:**
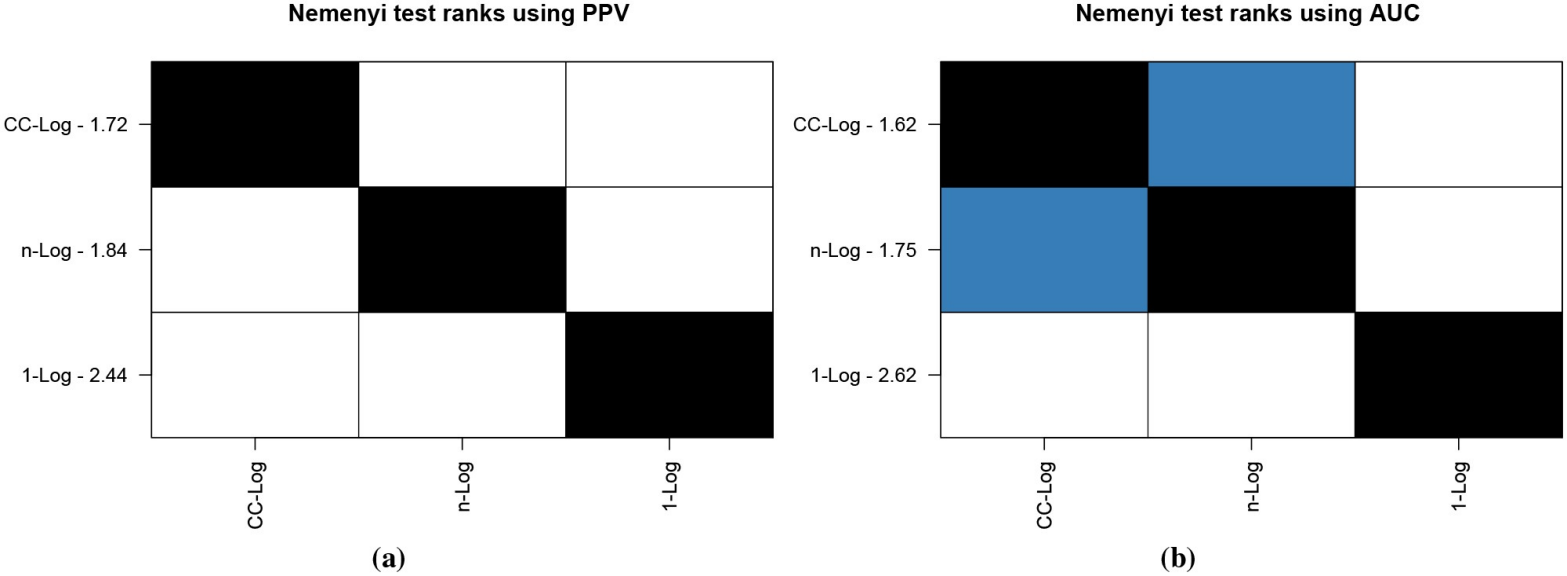
Nemenyi plots for fibre optic cable data using PPV and AUC results.

Friedman tests on AUC, PPV and NPV results gave *p*-values of 0.0048, 0.0045 and 0.7583 respectively. This shows that while AUC and PPV results differ significantly across classifiers, NPV results are similar. As such, we conduct Nemenyi tests on AUC and PPV values. Fig 34 shows Nemenyi test ranks for PPV and AUC, with lower ranks denoting better performance.

From Fig 34a we see that for PPV CC-Log outperforms *n*-Log and *n*-Log outperforms 1-Log with a 95% level of confidence. For AUC results, both CC-Log and *n*-Log significantly outperform 1-Log. Even though CC-Log outperforms *n*-Log, the two classifiers are not significantly different from each other as depicted by the blue squares in Fig 34b.

As NPV results across the classifiers are not significantly different, we turn our attention to PPV and AUC results in Table 4. We see that CC-Log performs better at earlier event ages compared to *n*-Log. Noting that the only difference between *n*-Log and CC-Log is the connections between the independent classifiers, this demonstrates the importance of the connections for early event classification. That is, the knowledge of “older” events aids classification of “younger” events.

### 7.3 Nitrogen dioxide monitoring

We consider monthly NO_2_ data from March to June for a 10 year period from 2010 to 2019. These are three dimensional datasets with two spatial and one time dimension. First we extract events using CPDBEE for each year separately. Then we perform 10-fold cross validation by training CC-Log, 1-Log and *n*-Log classifiers on event data of 9 years and testing it on the remaining year’s data.

**Fig 35:**
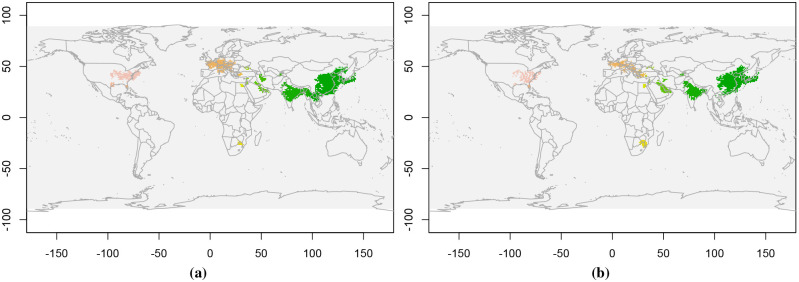
Events extracted from NO_2_ data from March to June 2018. Fig 35a and 35b show two dimensional cross sections of the three dimensional NO_2_ clusters in March and June 2018. Colours do not reflect NO_2_ levels. Each event is depicted by a single colour.

The extracted events are three dimensional clusters of high NO_2_ levels spanning space and time. Events are extracted from a data stream of 4 × 180 × 360 array, where each 180 × 360 matrix corresponds to the NO_2_ levels of a given month. We use CPDBEE parameters *α* = 0.97, *ϵ* = 2 and minPts = 20. Fig 35 shows two dimensional cross sections of the three dimensional NO_2_ clusters in March and June 2018.

For each 3-dimensional event we compute features 1–10 and 14 from the list of features in Section 6.1. These features are chosen for ease of computation. NO_2_ clusters which have grown in average intensity during this time period are assigned the class label 1 and others 0. As some NO_2_ clusters only start in April we designate the value in April as the starting value for class label computation. Thus, the task is to detect if NO_2_ clusters grow in intensity as soon as possible.

Table 5 gives the 10-fold cross validation test results on NO_2_ clusters. We see that CC-Log achieves better accuracy results compared to *n*-Log and 1-Log, with the only exception that 1-Log achieves slightly better results at *t*_4_.

**Table 5:** 10-Fold cross validation accuracy comparison on NO_2_ clusters.

Accuracy Measure	Classifier	Mean				Standard deviation			
		*t*_1_	*t*_2_	*t*_3_	*t*_4_	*t*_1_	*t*_2_	*t*_3_	*t*_4_
Classification Accuracy	CC-Log	0.84	0.81	0.90	0.88	0.08	0.12	0.07	0.07
	1-Log	0.80	0.80	0.88	0.89	0.09	0.11	0.07	0.07
	*n*-Log	0.80	0.80	0.87	0.83	0.09	0.12	0.06	0.11

#### 7.3.1 Significance results

Similar to the previous applications, we perform a Friedman test on the classification accuracy results. The Friedman test gave a *p*-value of 0.01752, showing that there is a significant difference between the classifiers.

**Fig 36:**
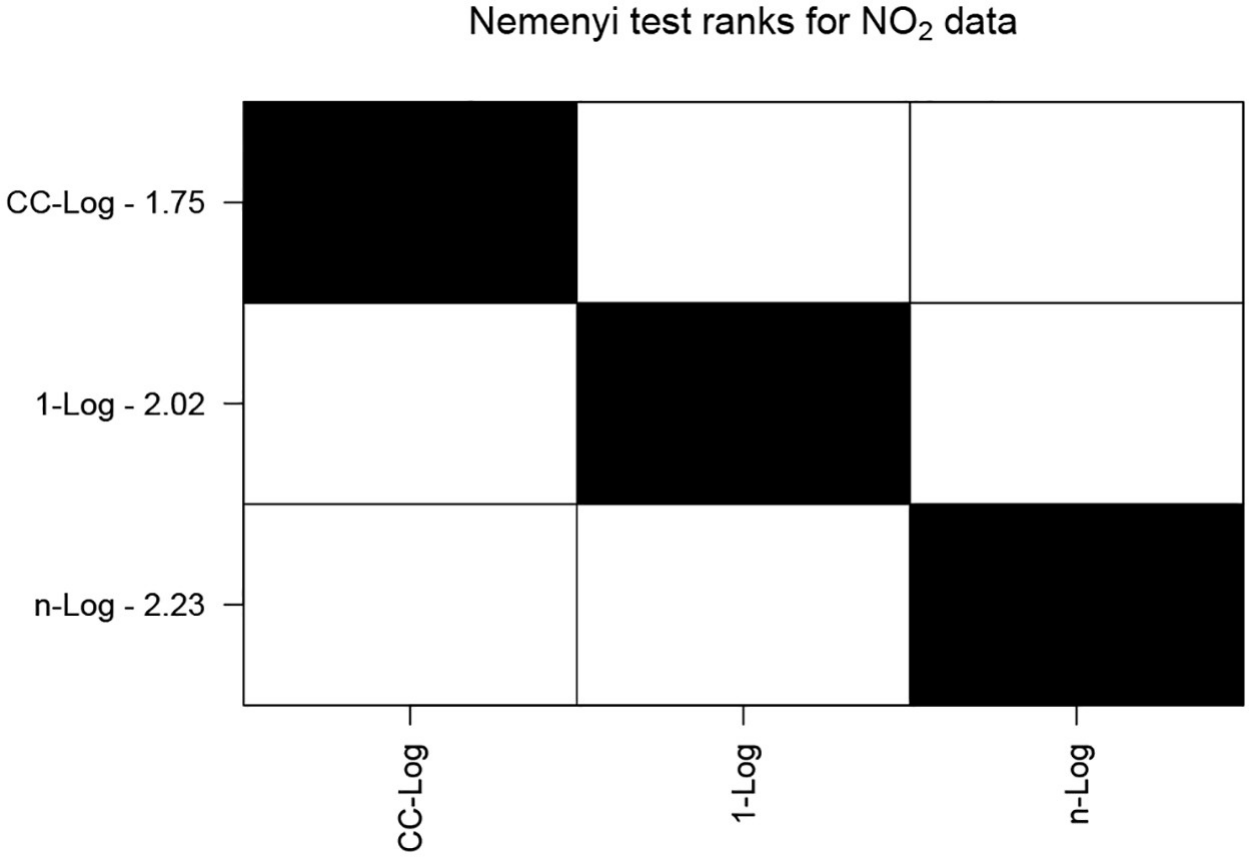
Nemenyi plot for NO_2_ data using classification accuracy results.

Fig 36 shows the resulting Nemenyi plot, which shows that CC-Log outperforms 1-Log and *n*-Log with a 5% level of significance. The success of CC-Log demonstrates the benefit of linking classifiers that are trained on similar but slightly different data, on early event classification.

## 8 Conclusions

This paper has proposed a framework for event extraction and early event classification in contiguous spatio-temporal data streams. We proposed an event detection and extraction algorithm as well as an early event classification algorithm. We tested our event detection and classification framework using 3 applications, one synthetic and two real.

The event extraction algorithm CPDBEE uses change point detection and clustering techniques to detect and extract events. We compared CPDBEE with Kuldorff’s Scan Statistic and achieved better results for all three applications in a much shorter time period.

The early event classification algorithm comprises of a set of base classifiers connected using an *L*_2_ penalty term, inducing a certain level of smoothness in event age. We compared the connected classifier CC-Log, with two configurations of unlinked classifiers 1-Log and *n*-Log and achieved better results for all three applications. Furthermore, for all three applications CC-Log achieved better results for early event ages. As the only difference between *n*-Log and CC-Log was the connections between the base classifiers, this reveals that classification of early events benefits from knowledge of more mature events.

Future directions for this research include extending CPDBEE for non-contiguous spatio-temporal data as well as extending CC to use other base classifiers such as decision trees. Furthermore, currently CC is a single class classifier. In addition, CC does not have the functionality for selecting the best set of features. Extending CC to handle multi-classes and adding feature selection functionality are also plans for the future.

## Supporting information

S1 File [35, 50–59].
(R) Click here for additional data file.

S2 File (R)

S1 Data (R)

S2 Data (R)

S3 Data (R)

S4 Data (R)

S1 Appendix (PDF)
