# Supplementary material for: Early classification of spatio-temporal events using partial information
Source: PLoS One. 2020 Aug 5;15(8):e0236331. doi: 10.1371/journal.pone.0236331 (PMC7406362; doi:10.1371/journal.pone.0236331)
Supplement: S1 File — [35, 50–59]. (R) [file pone.0236331.s001.pdf]

R-package eventstream: This package contains CPDBEE algorithm for event extraction, the connected classifier CC-Log for early event classification, functions for synthetic data generation, the fibre-optic data stream and NO_2 data. It is available from GitHub at `https://github.com/sevvandi/eventstream`.

Scripts: The file `Supp_Mat_CPDBEE` contains the code used in Section 4. There are three files containing the R code used in Section 7. The files `Supp_Mat_1.R`, `Supp_Mat_2.R` and `Supp_Mat_3.R` contain the code applicable for synthetic data, fibre optic data and NO_2 data respectively.

Other R-packages: We have used the following R-packages either in this paper or within the package *eventstream*: *changepoint* [35], *abind* [50], *AtmRay* [51], *pROC* [52], *ggplot2* [53], *raster* [54], *maps* [55], *tensorA* [56], *glmnet* [57], *dbscan* [58] and *MASS* [59].
