# Supplementary figures and images for: Early classification of spatio-temporal events using partial information
Source: PLoS One. 2020 Aug 5;15(8):e0236331. doi: 10.1371/journal.pone.0236331 (PMC7406362; doi:10.1371/journal.pone.0236331)

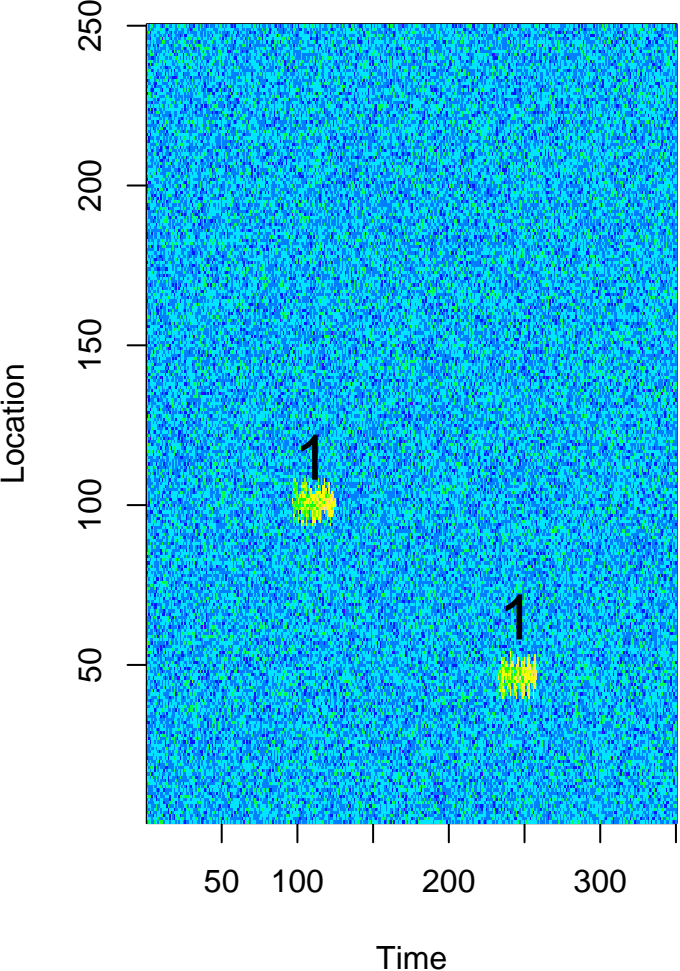

Supplement: S2 File — (R) [file pone.0236331.s002.zip › Graphics/2_A_Blobs_labels.pdf]

Location

250

200

150

100

50

50 100

200

300

Time

1

2

3

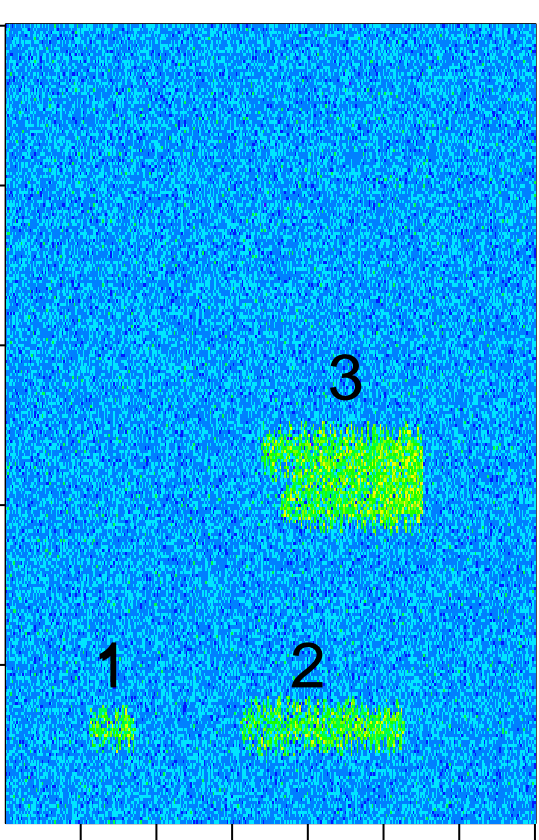

Supplement: S2 File — (R) [file pone.0236331.s002.zip › Graphics/3_B_Blobs_labels.pdf]

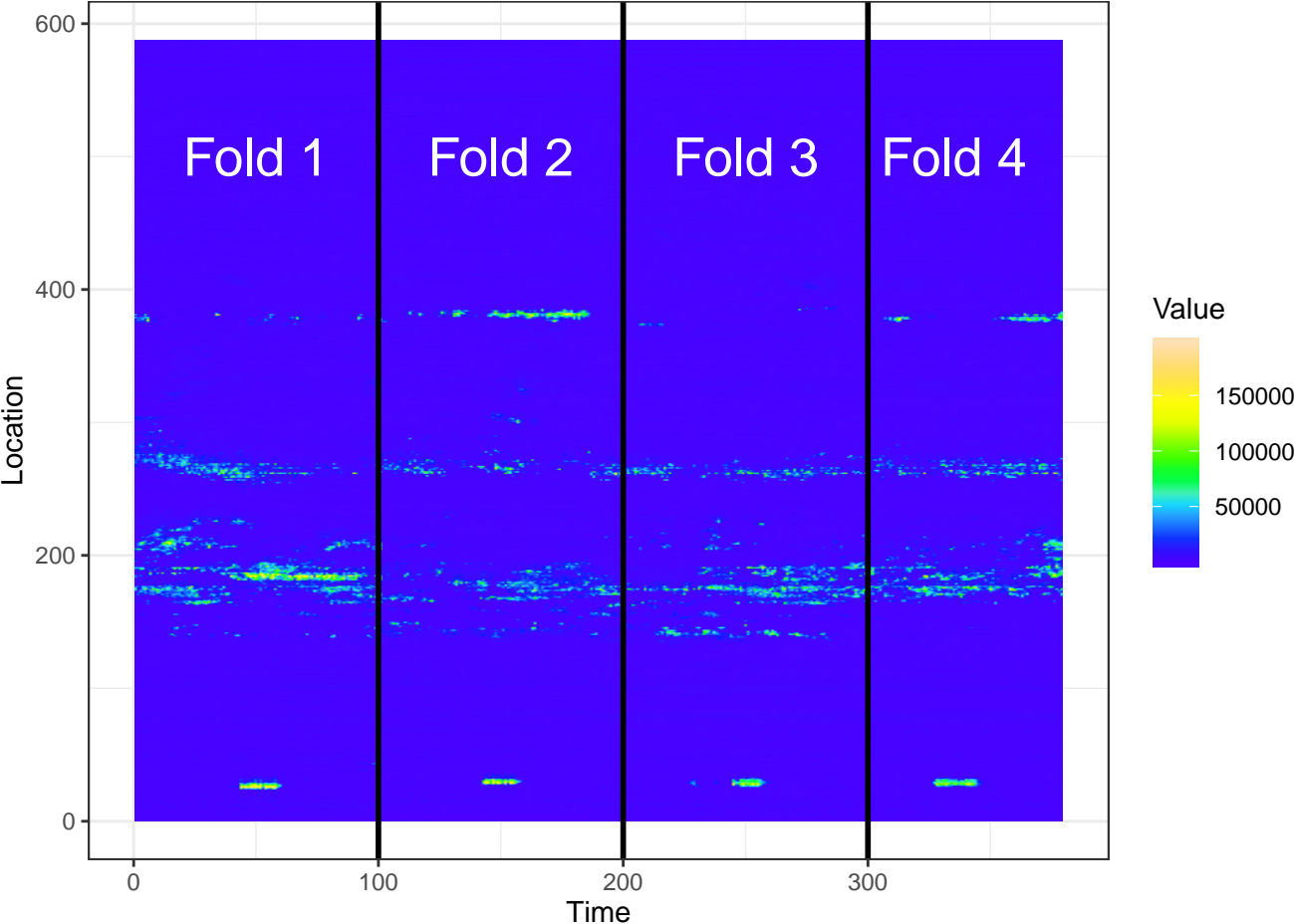

Supplement: S2 File — (R) [file pone.0236331.s002.zip › Graphics/4Fold_CV.pdf]

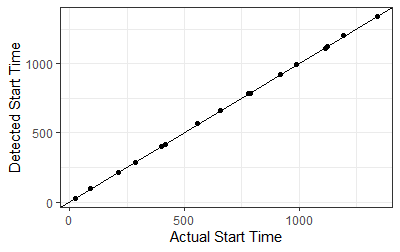

Supplement: S2 File — (R) [file pone.0236331.s002.zip › Graphics/Actual_vs_Detected.png]

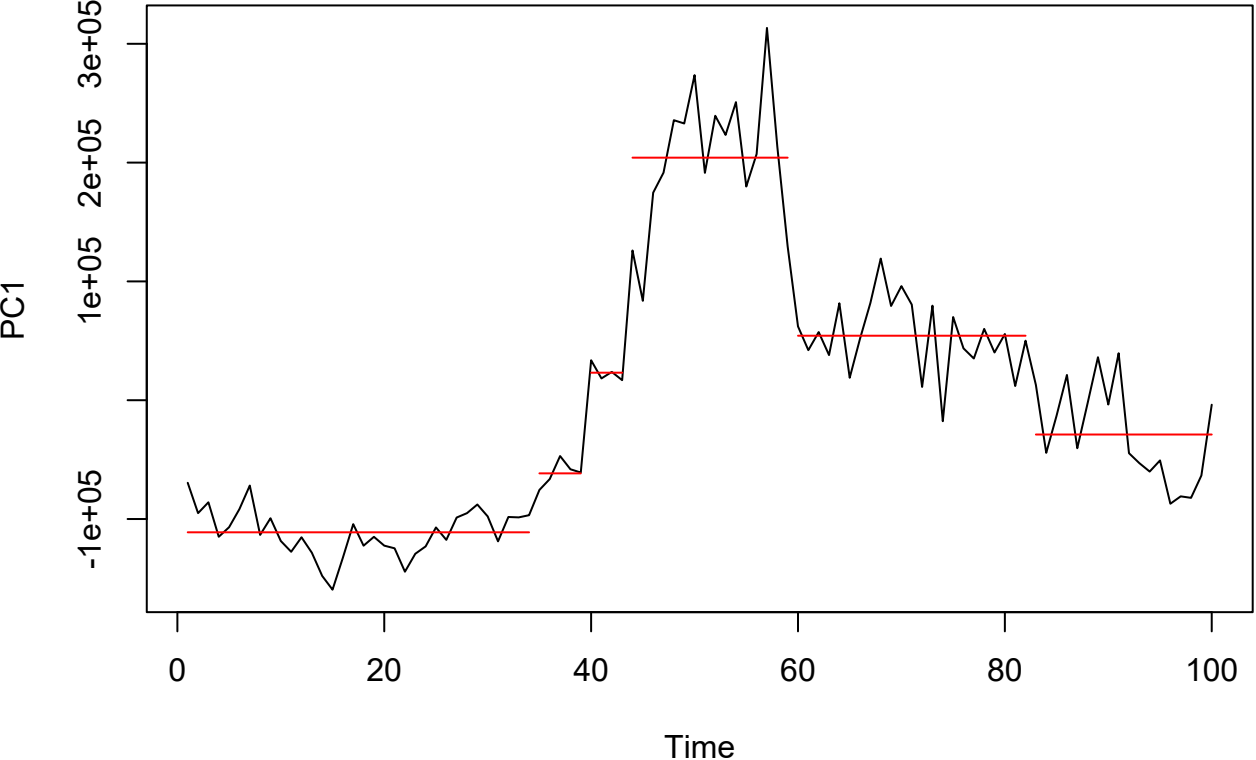

Supplement: S2 File — (R) [file pone.0236331.s002.zip › Graphics/changepoint1.pdf]

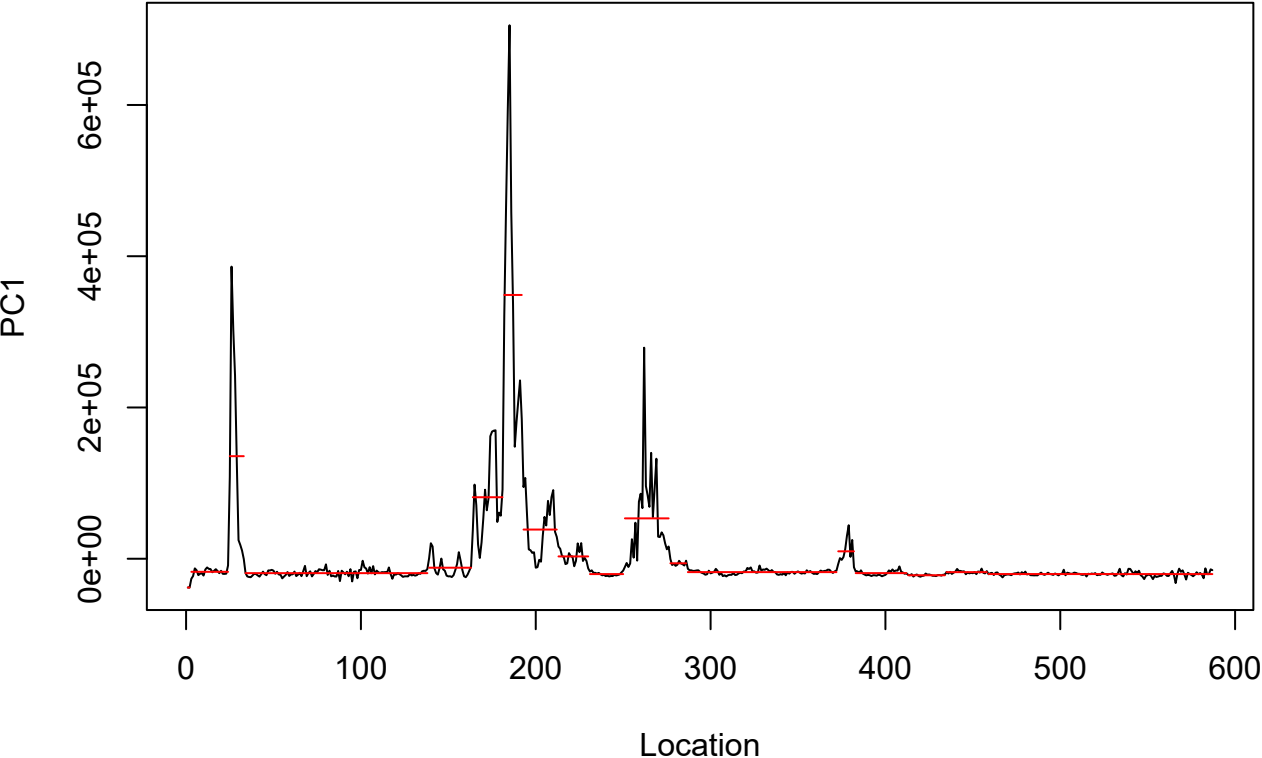

Supplement: S2 File — (R) [file pone.0236331.s002.zip › Graphics/changepoint2.pdf]

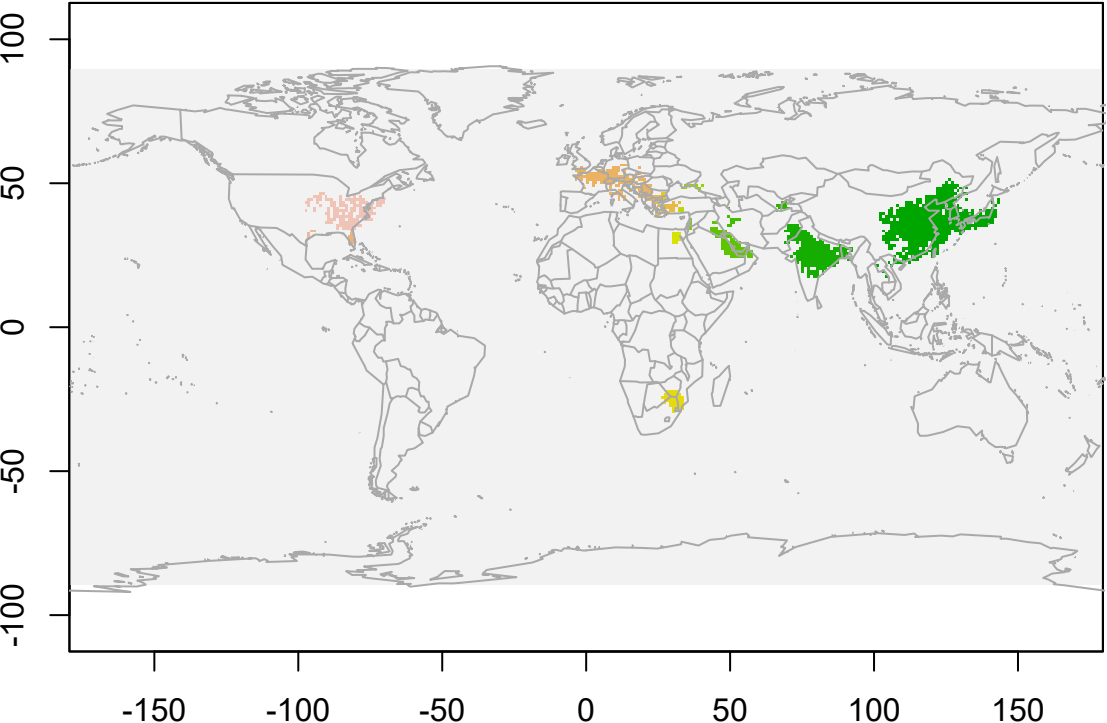

Supplement: S2 File — (R) [file pone.0236331.s002.zip › Graphics/Clusters_NO2_June_2018_With_Bndry.pdf]

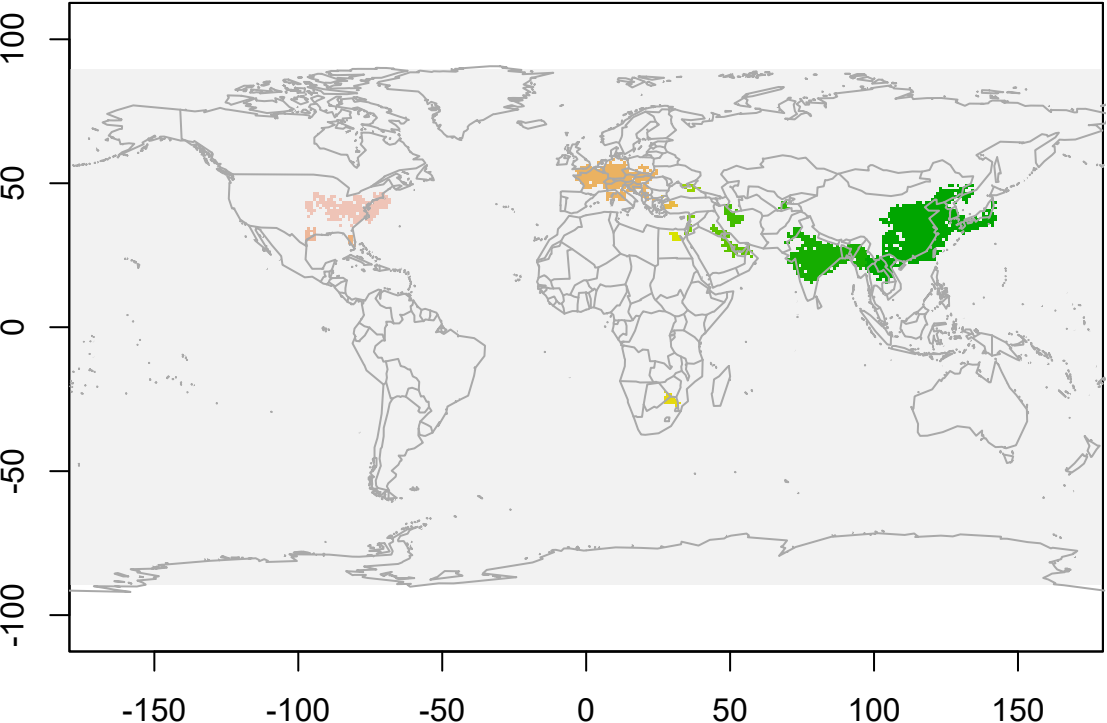

Supplement: S2 File — (R) [file pone.0236331.s002.zip › Graphics/Clusters_NO2_March_2018_With_Bndry.pdf]

**Original**

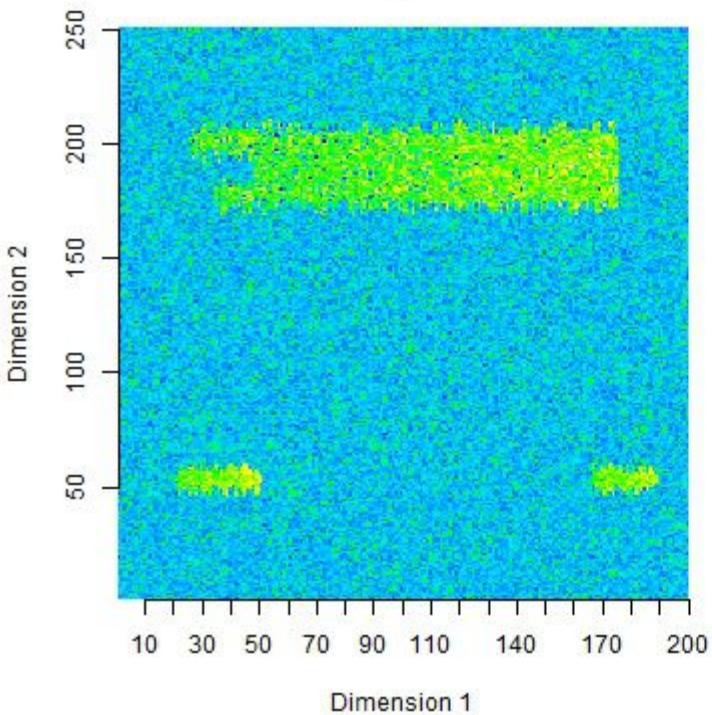

**Events**

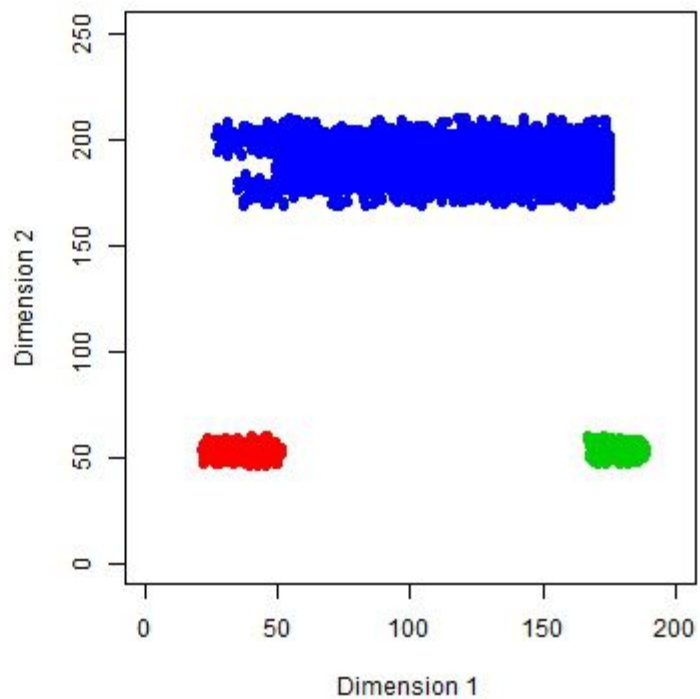

Supplement: S2 File — (R) [file pone.0236331.s002.zip › Graphics/Events_100040.pdf]

**Original**

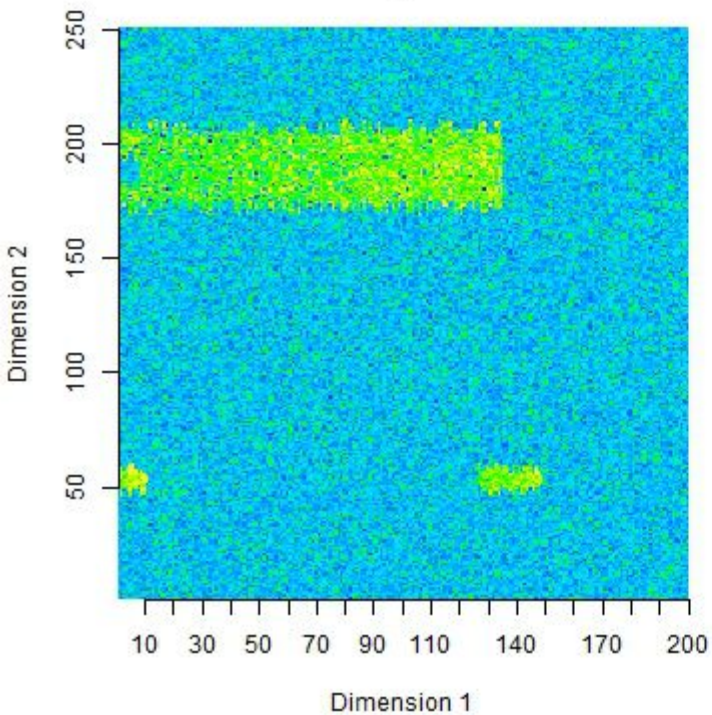

**Events**

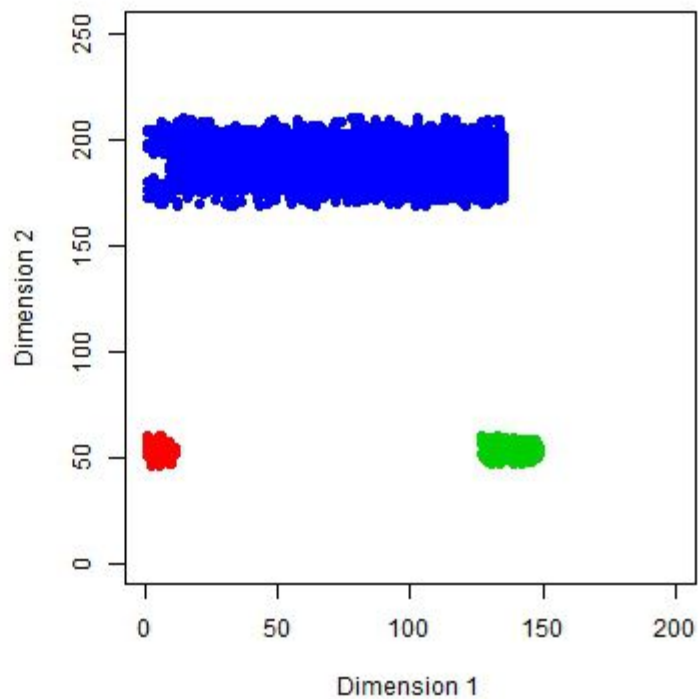

Supplement: S2 File — (R) [file pone.0236331.s002.zip › Graphics/Events_100044.pdf]

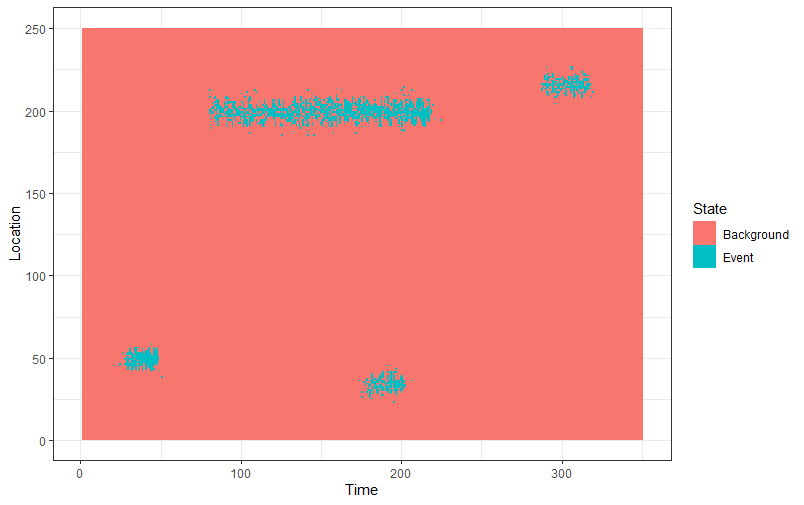

Supplement: S2 File — (R) [file pone.0236331.s002.zip › Graphics/Events_Detected_Synth.png]

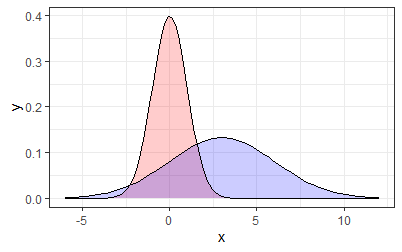

Supplement: S2 File — (R) [file pone.0236331.s002.zip › Graphics/event_background_1.png]

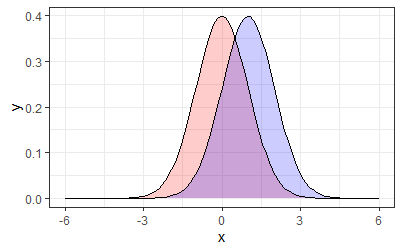

Supplement: S2 File — (R) [file pone.0236331.s002.zip › Graphics/event_background_2.png]

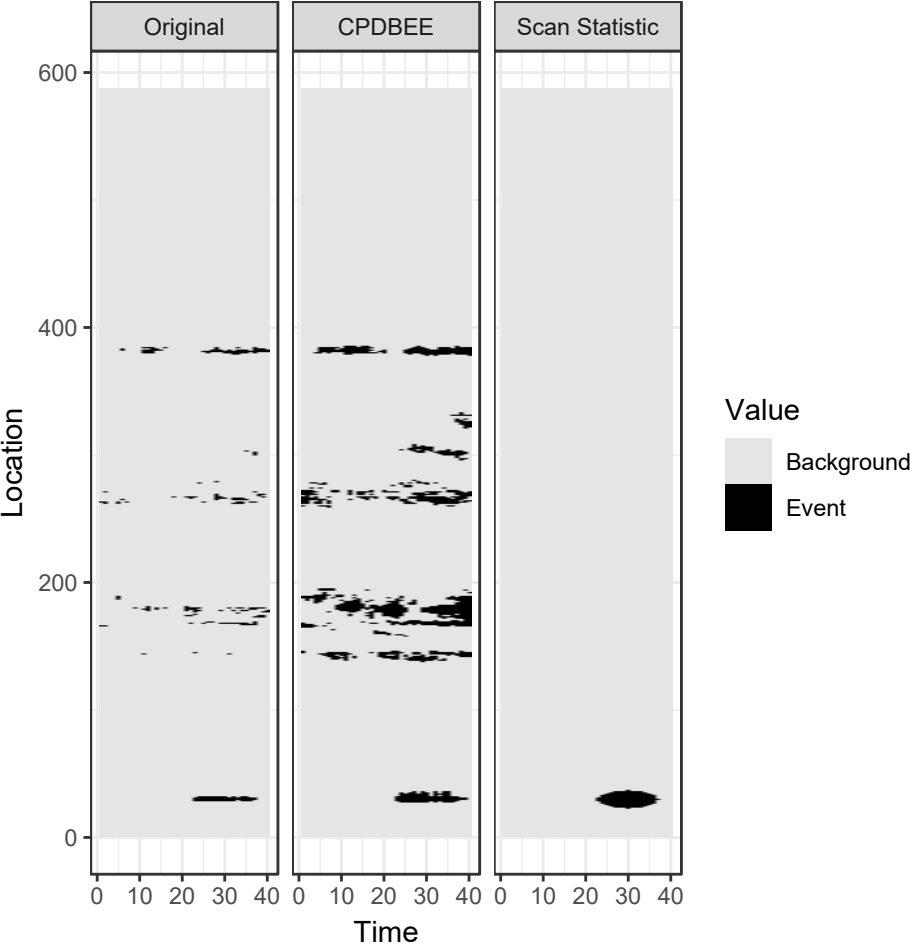

Supplement: S2 File — (R) [file pone.0236331.s002.zip › Graphics/Event_Comparison_121_160.pdf]

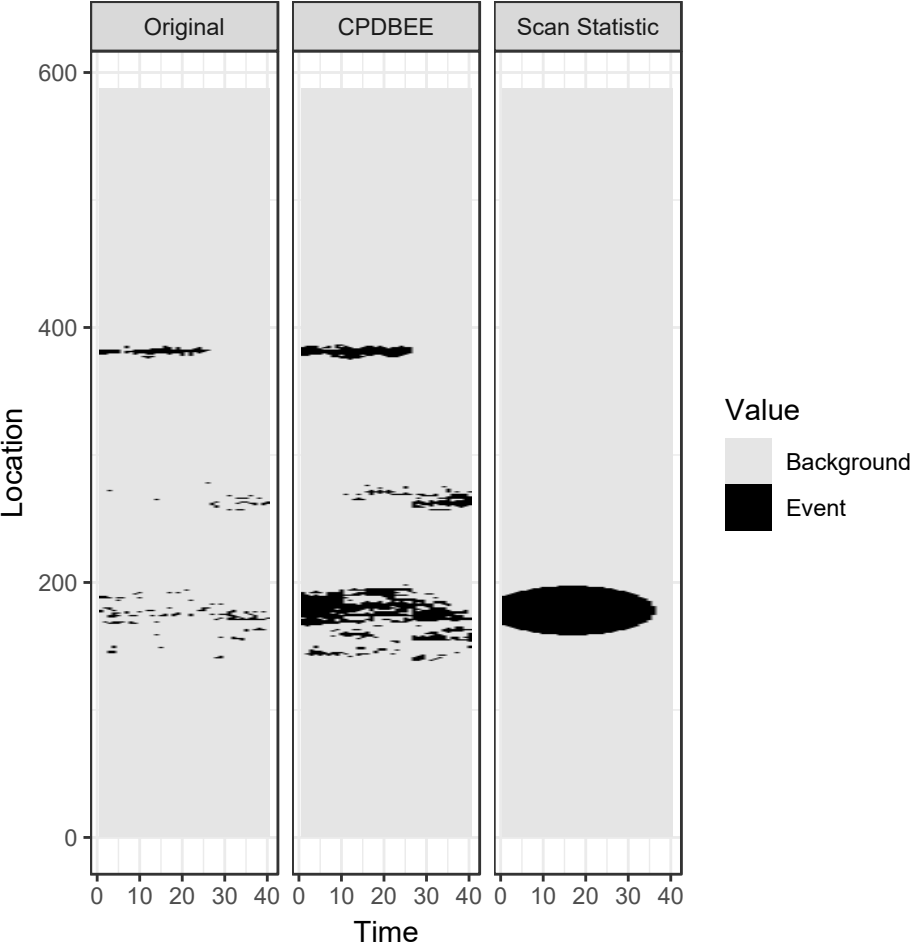

Supplement: S2 File — (R) [file pone.0236331.s002.zip › Graphics/Event_Comparison_161_200.pdf]

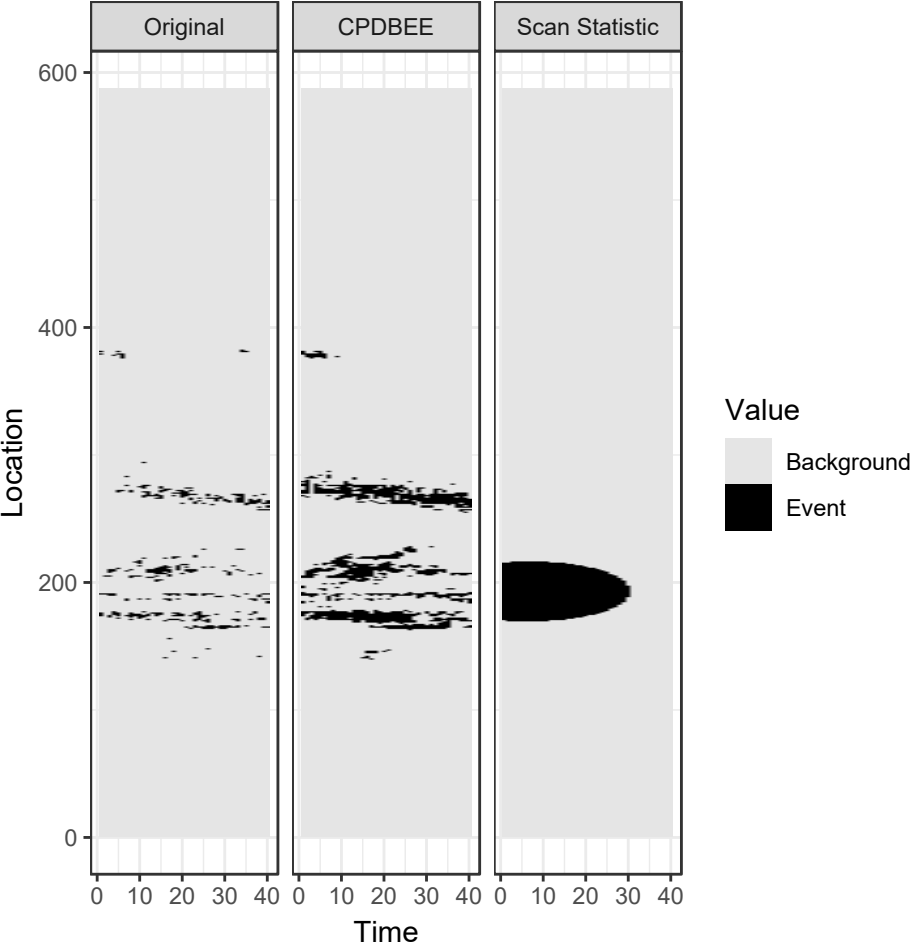

Supplement: S2 File — (R) [file pone.0236331.s002.zip › Graphics/Event_Comparison_1_40.pdf]

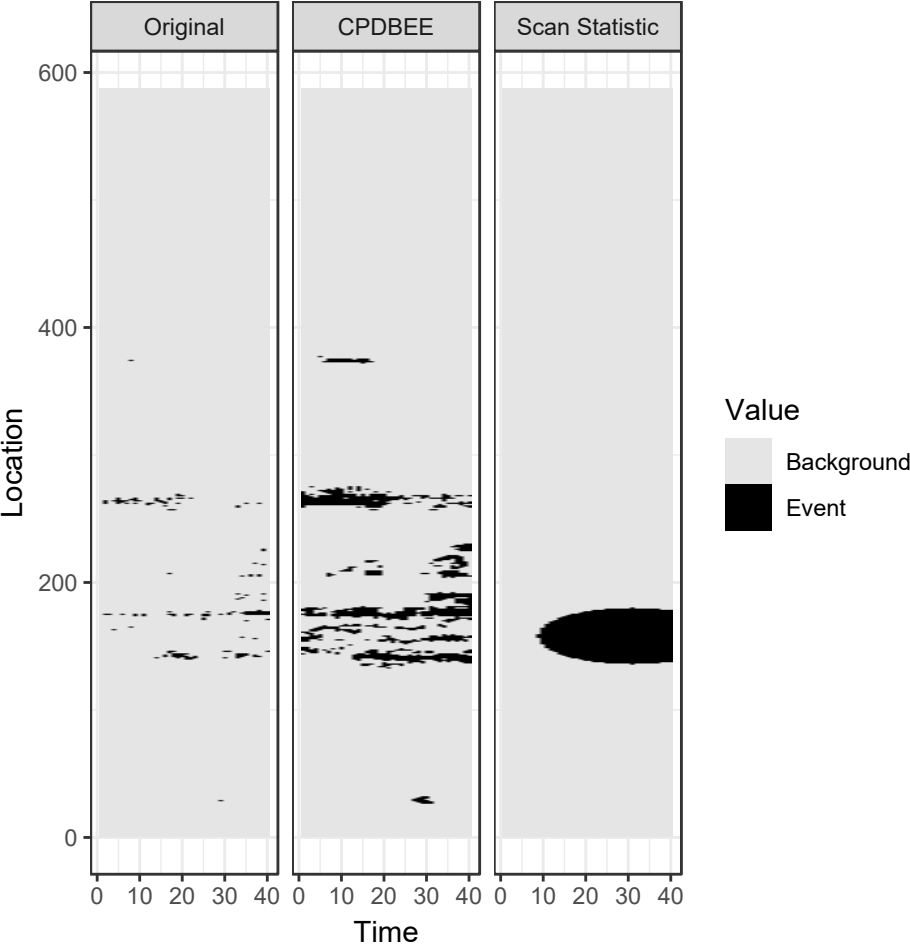

Supplement: S2 File — (R) [file pone.0236331.s002.zip › Graphics/Event_Comparison_201_240.pdf]

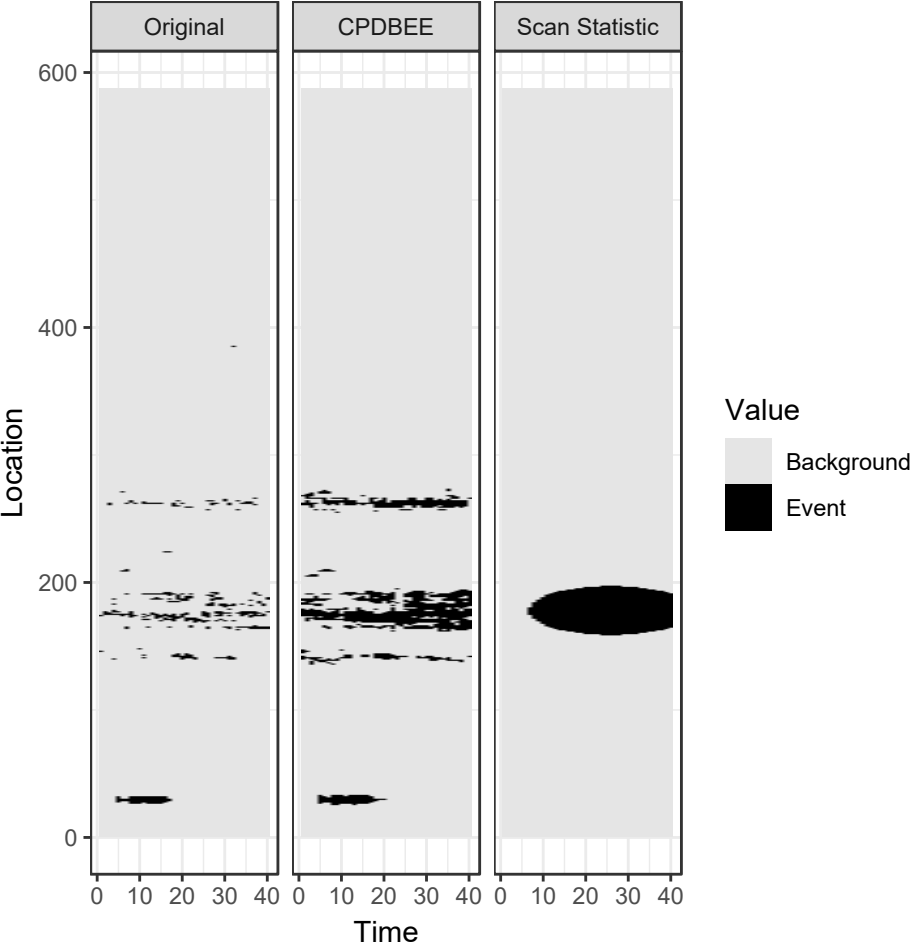

Supplement: S2 File — (R) [file pone.0236331.s002.zip › Graphics/Event_Comparison_241_280.pdf]

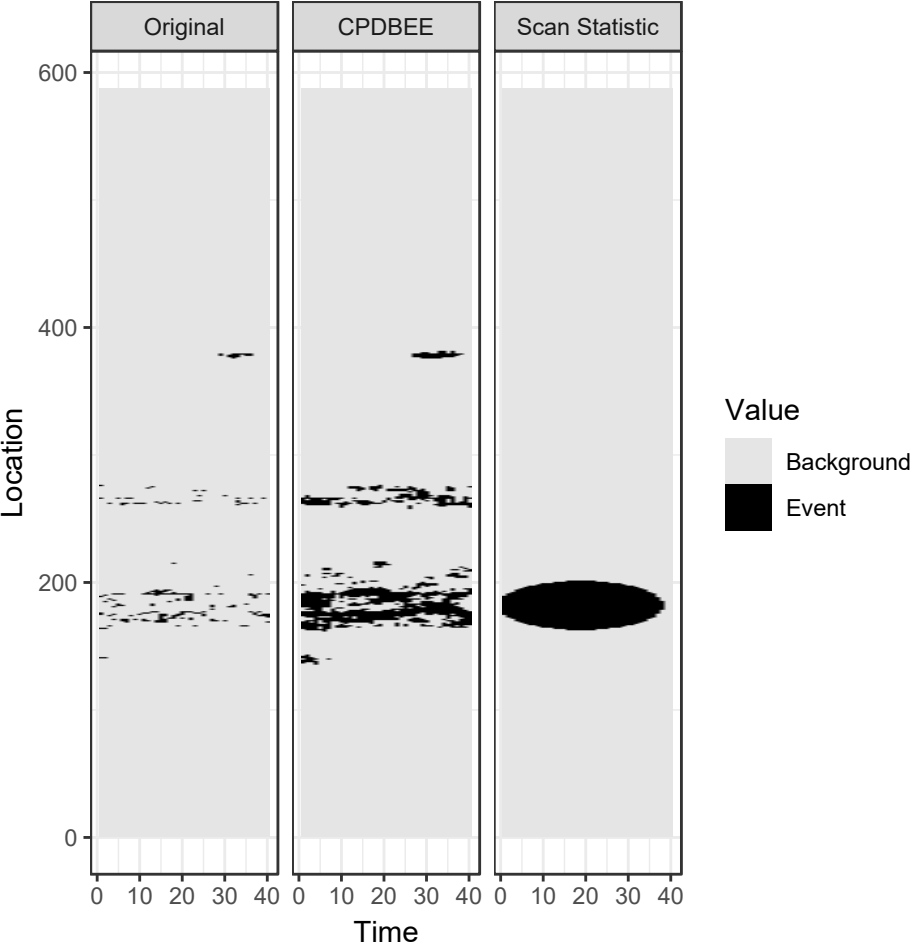

Supplement: S2 File — (R) [file pone.0236331.s002.zip › Graphics/Event_Comparison_281_320.pdf]

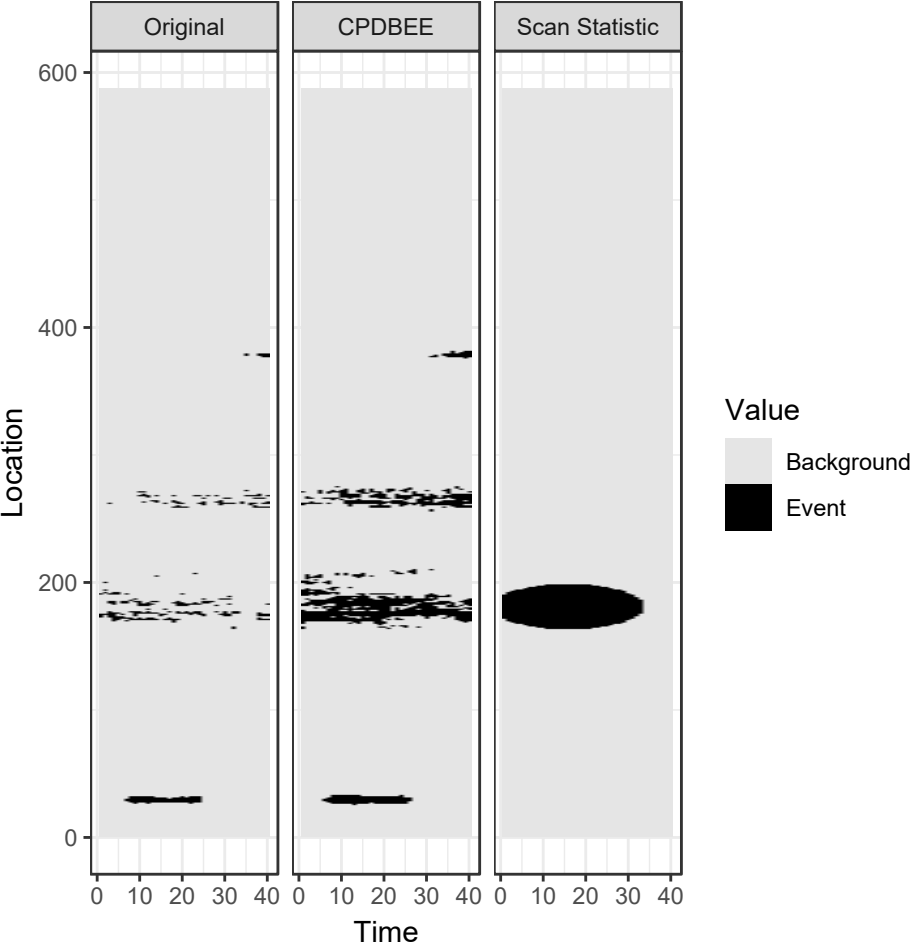

Supplement: S2 File — (R) [file pone.0236331.s002.zip › Graphics/Event_Comparison_321_360.pdf]

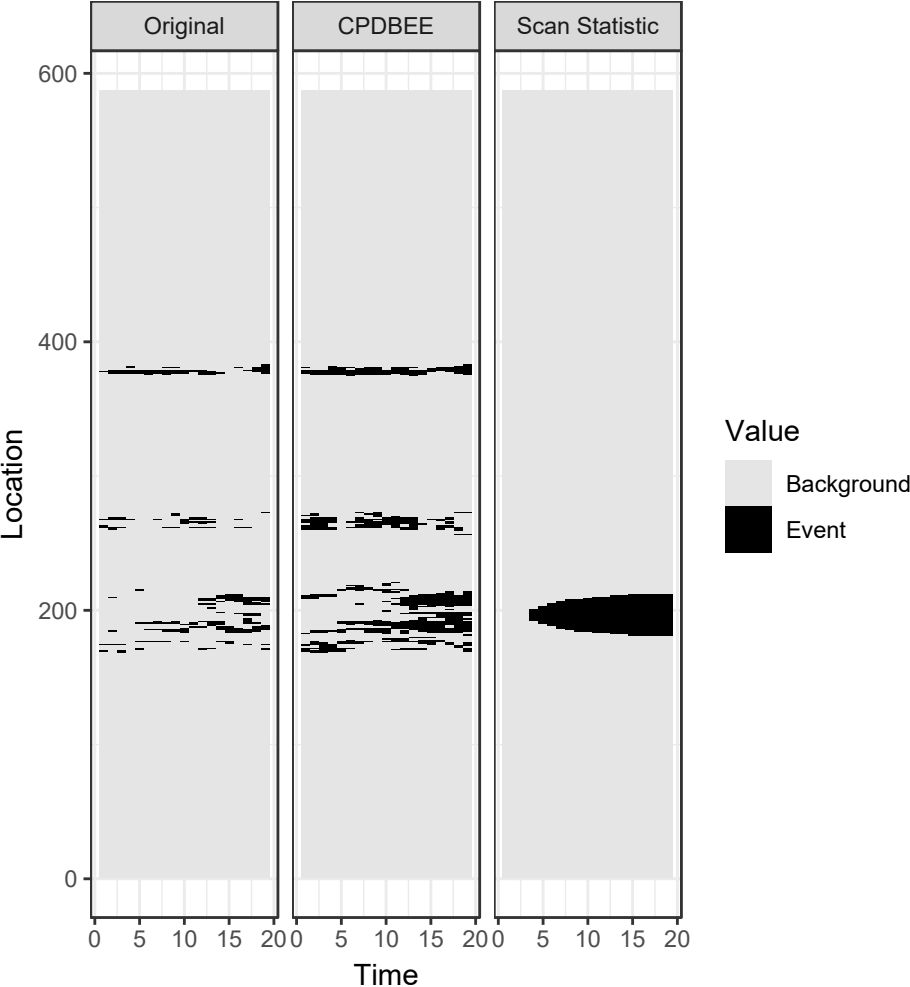

Supplement: S2 File — (R) [file pone.0236331.s002.zip › Graphics/Event_Comparison_361_379.pdf]

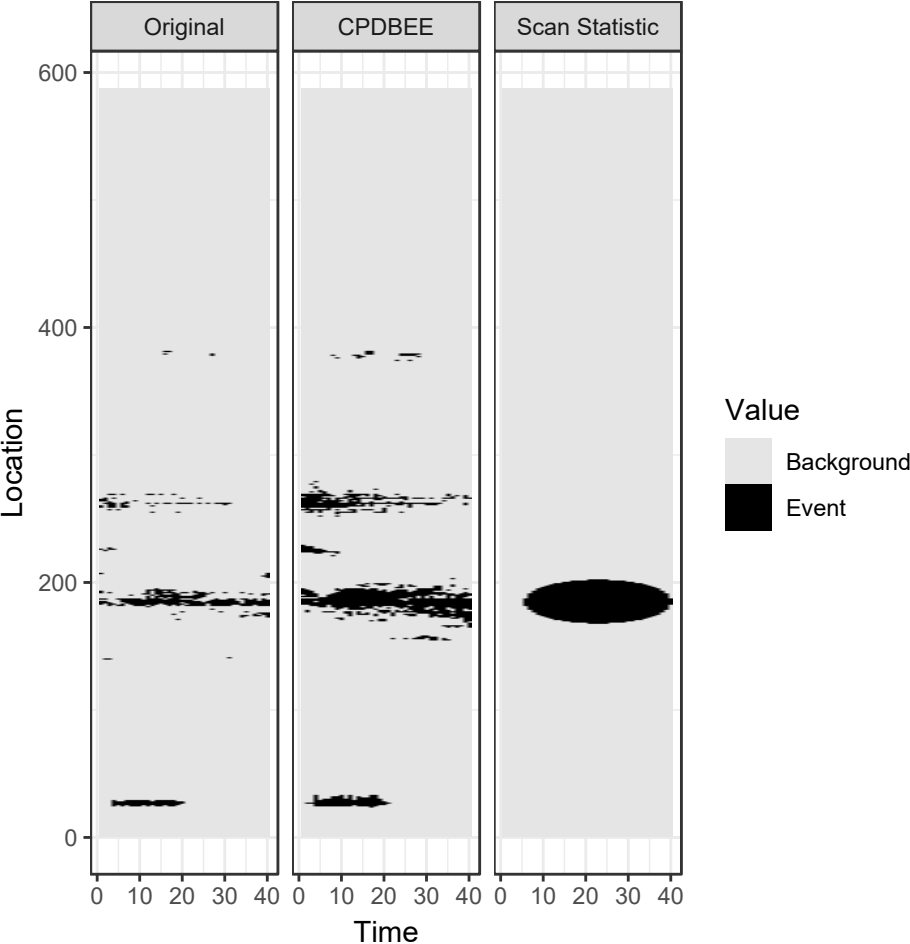

Supplement: S2 File — (R) [file pone.0236331.s002.zip › Graphics/Event_Comparison_41_80.pdf]

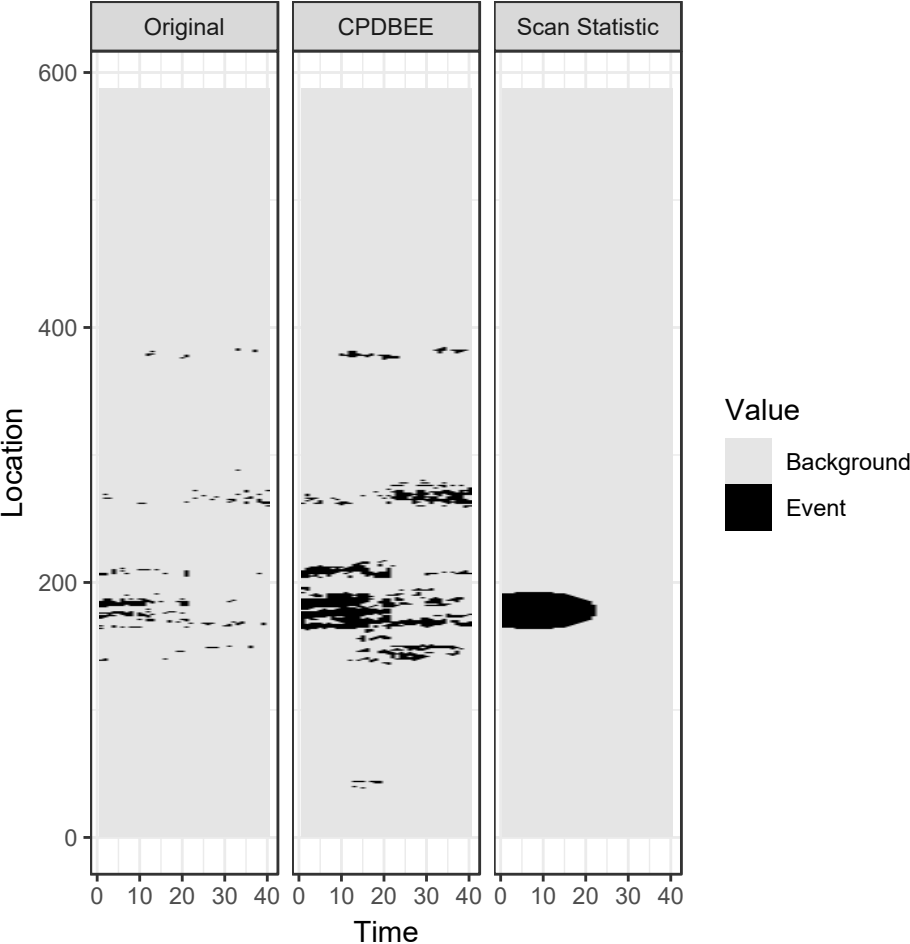

Supplement: S2 File — (R) [file pone.0236331.s002.zip › Graphics/Event_Comparison_81_120.pdf]

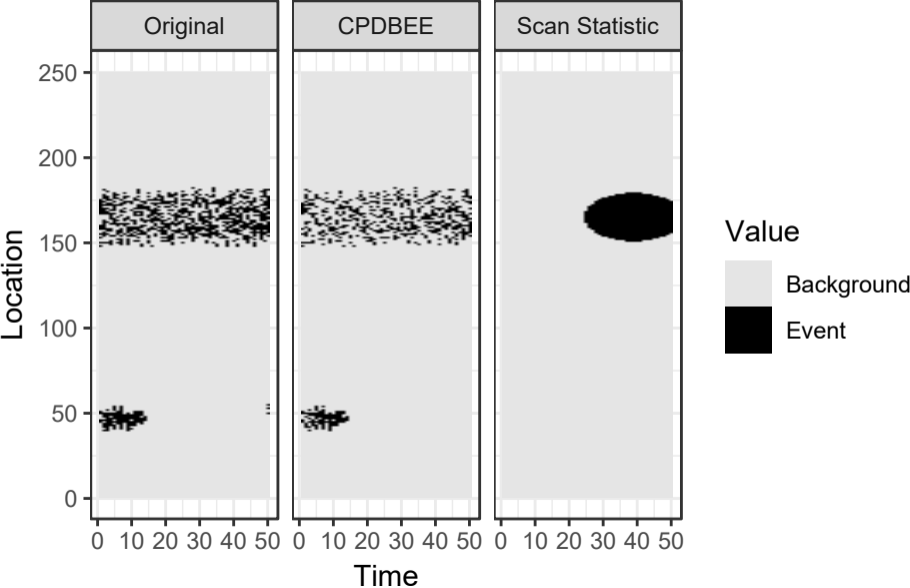

Supplement: S2 File — (R) [file pone.0236331.s002.zip › Graphics/Event_comparison_Synthetic_101_150.pdf]

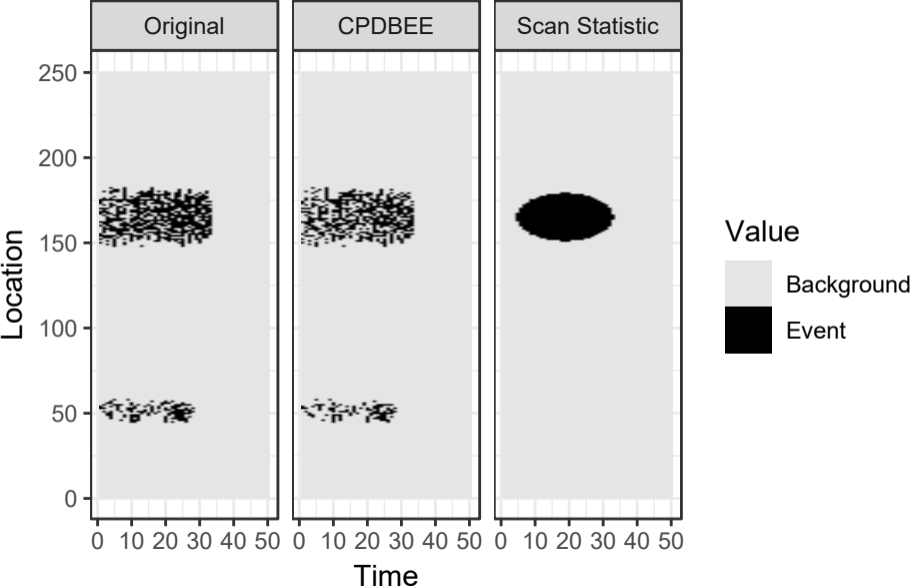

Supplement: S2 File — (R) [file pone.0236331.s002.zip › Graphics/Event_comparison_Synthetic_151_200.pdf]

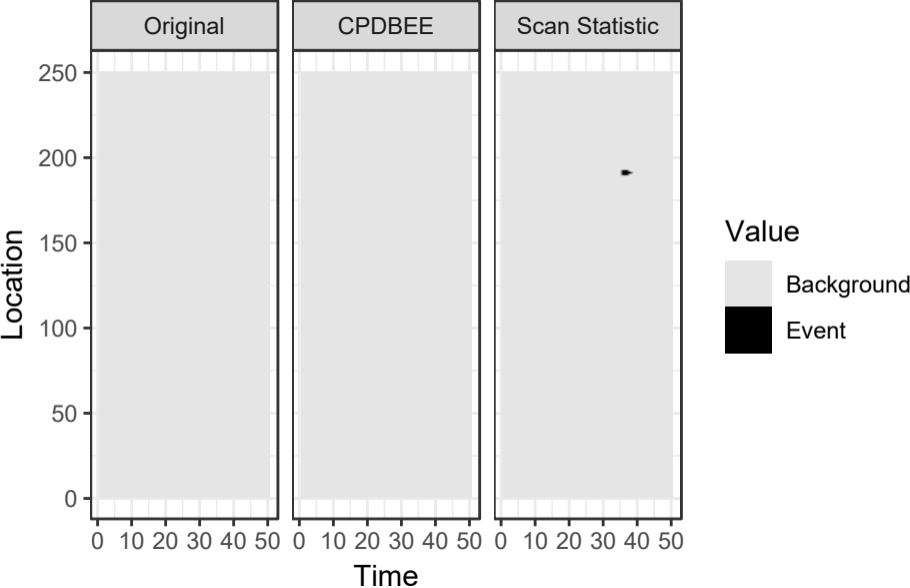

Supplement: S2 File — (R) [file pone.0236331.s002.zip › Graphics/Event_comparison_Synthetic_1_50.pdf]

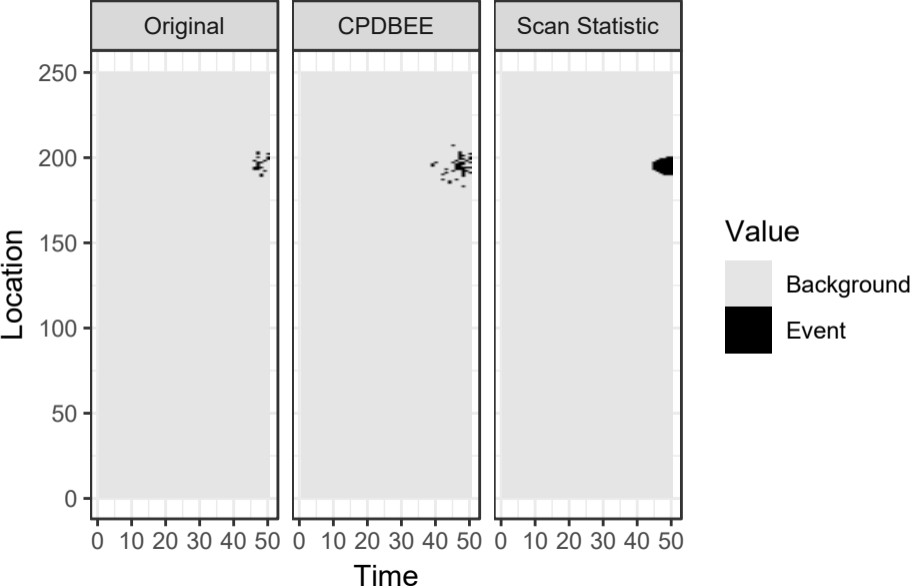

Supplement: S2 File — (R) [file pone.0236331.s002.zip › Graphics/Event_comparison_Synthetic_251_300.pdf]

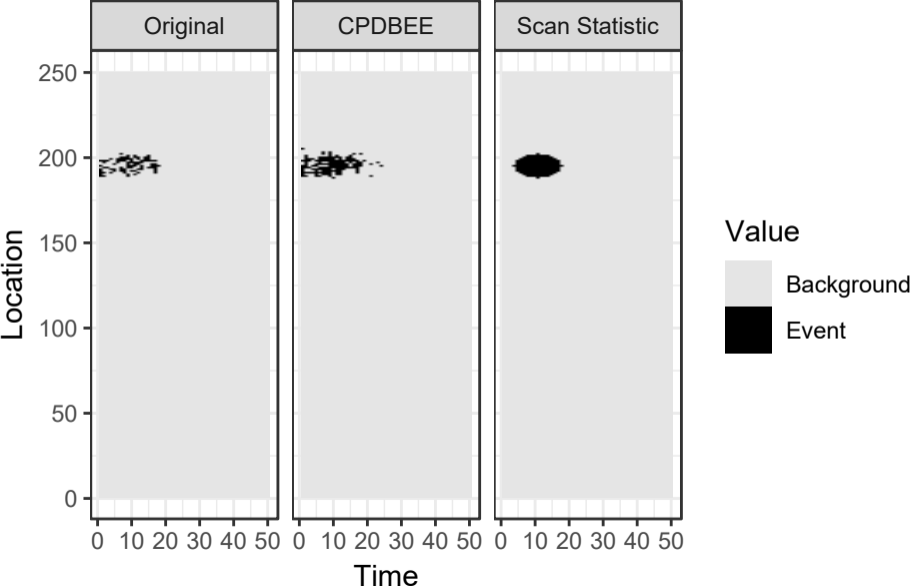

Supplement: S2 File — (R) [file pone.0236331.s002.zip › Graphics/Event_comparison_Synthetic_301_350.pdf]

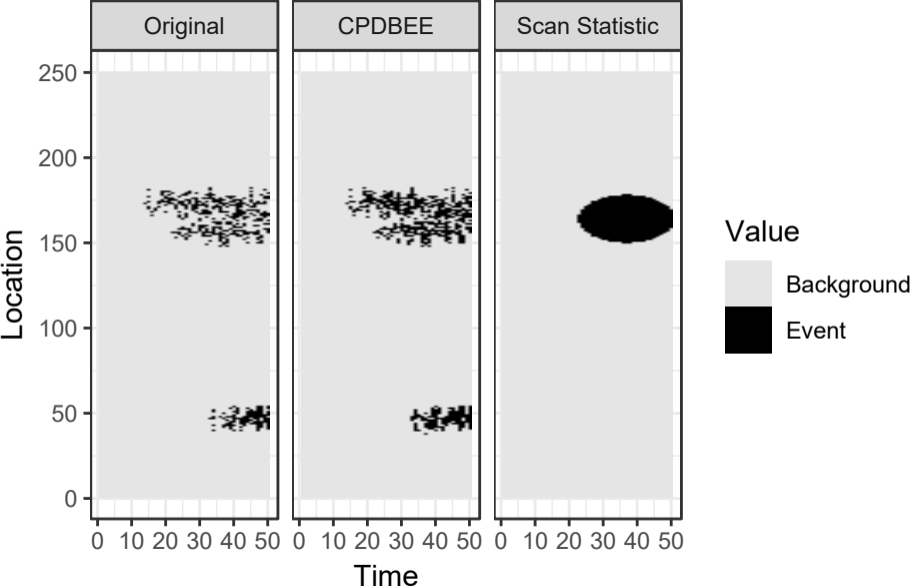

Supplement: S2 File — (R) [file pone.0236331.s002.zip › Graphics/Event_comparison_Synthetic_51_100.pdf]

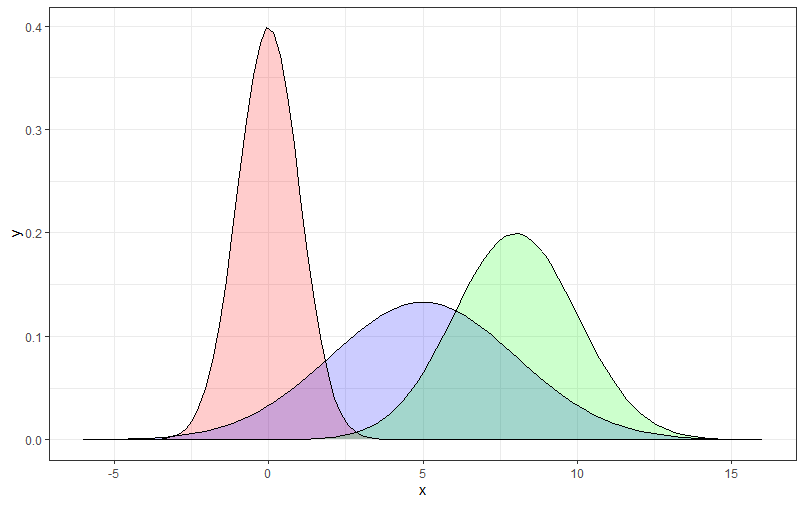

Supplement: S2 File — (R) [file pone.0236331.s002.zip › Graphics/Event_end_distribution.png]

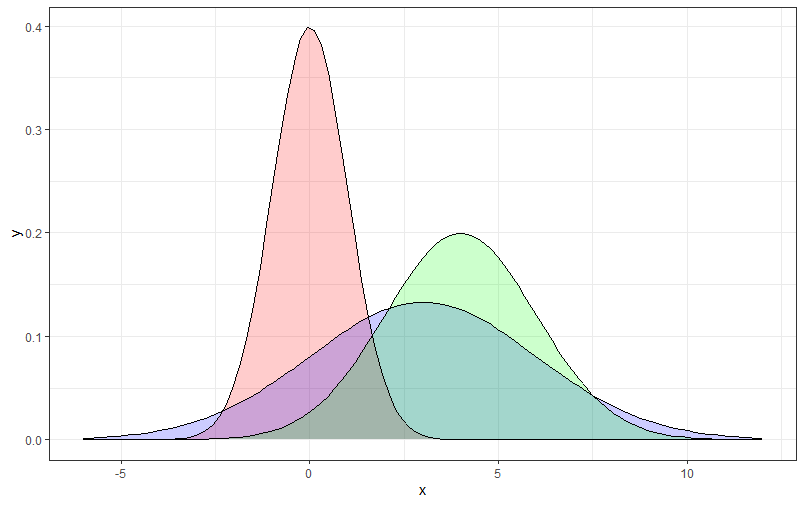

Supplement: S2 File — (R) [file pone.0236331.s002.zip › Graphics/Event_start_distribution.png]

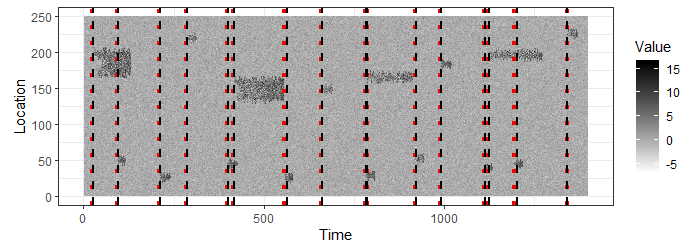

Supplement: S2 File — (R) [file pone.0236331.s002.zip › Graphics/fast_evolving.png]

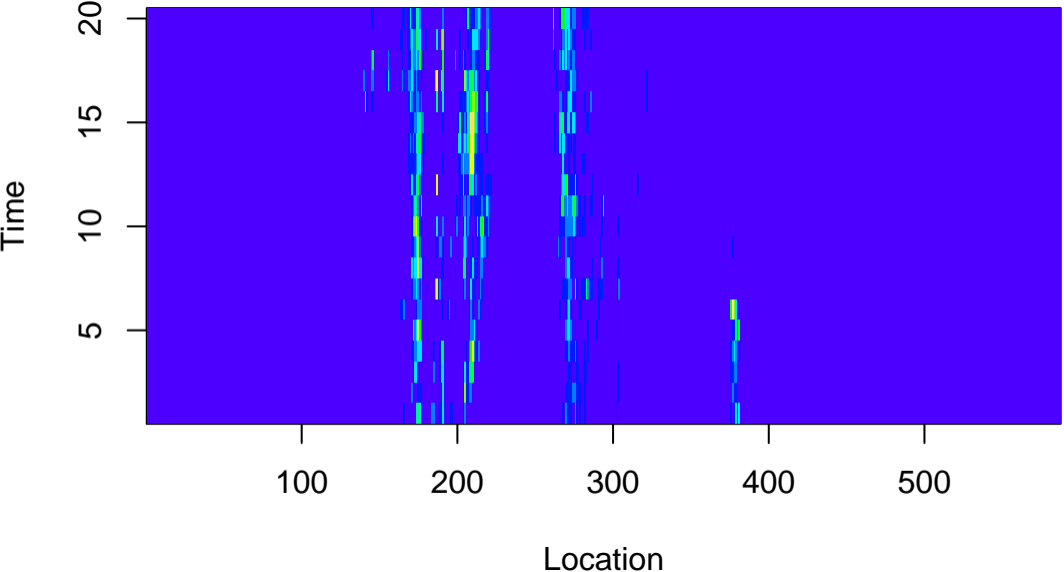

Supplement: S2 File — (R) [file pone.0236331.s002.zip › Graphics/Image_for_Splines.pdf]

# Nemenyi test ranks for NO<sub>2</sub> data

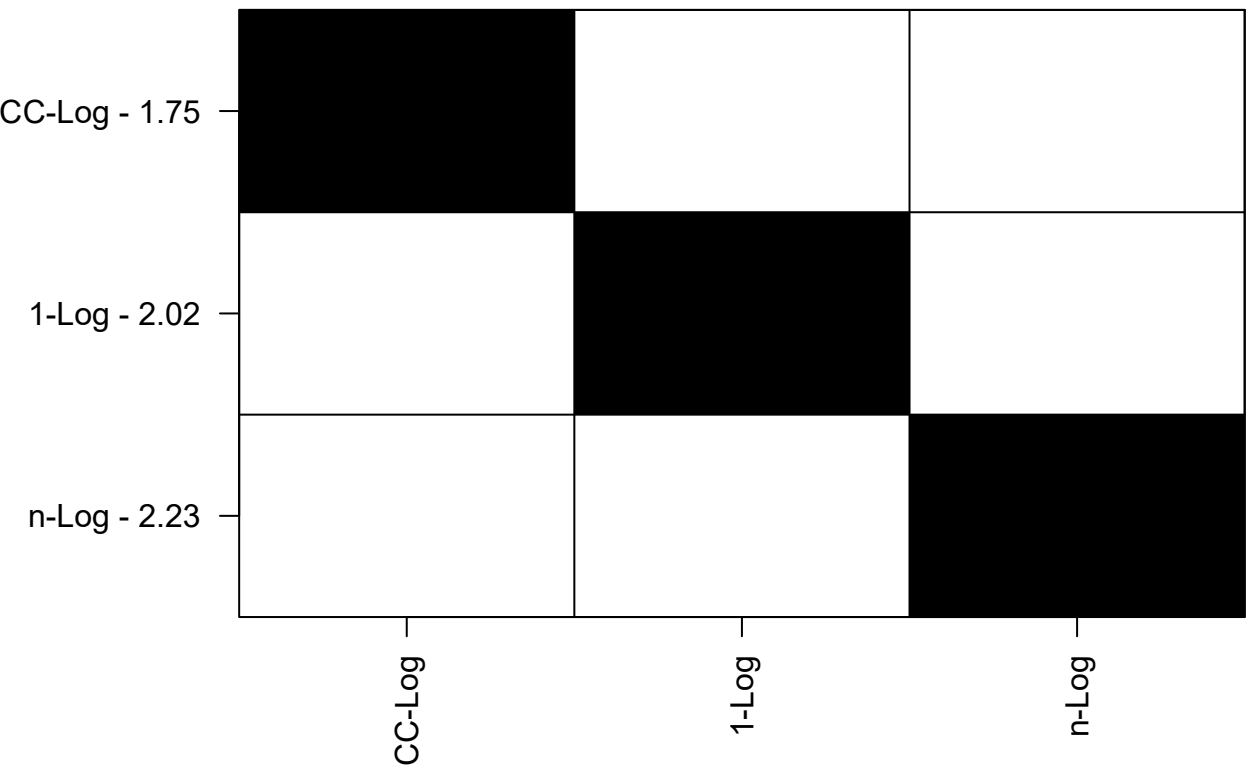

Supplement: S2 File — (R) [file pone.0236331.s002.zip › Graphics/Nemenyi_Accuracy_NO2.pdf]

# Nemenyi test ranks using PPV

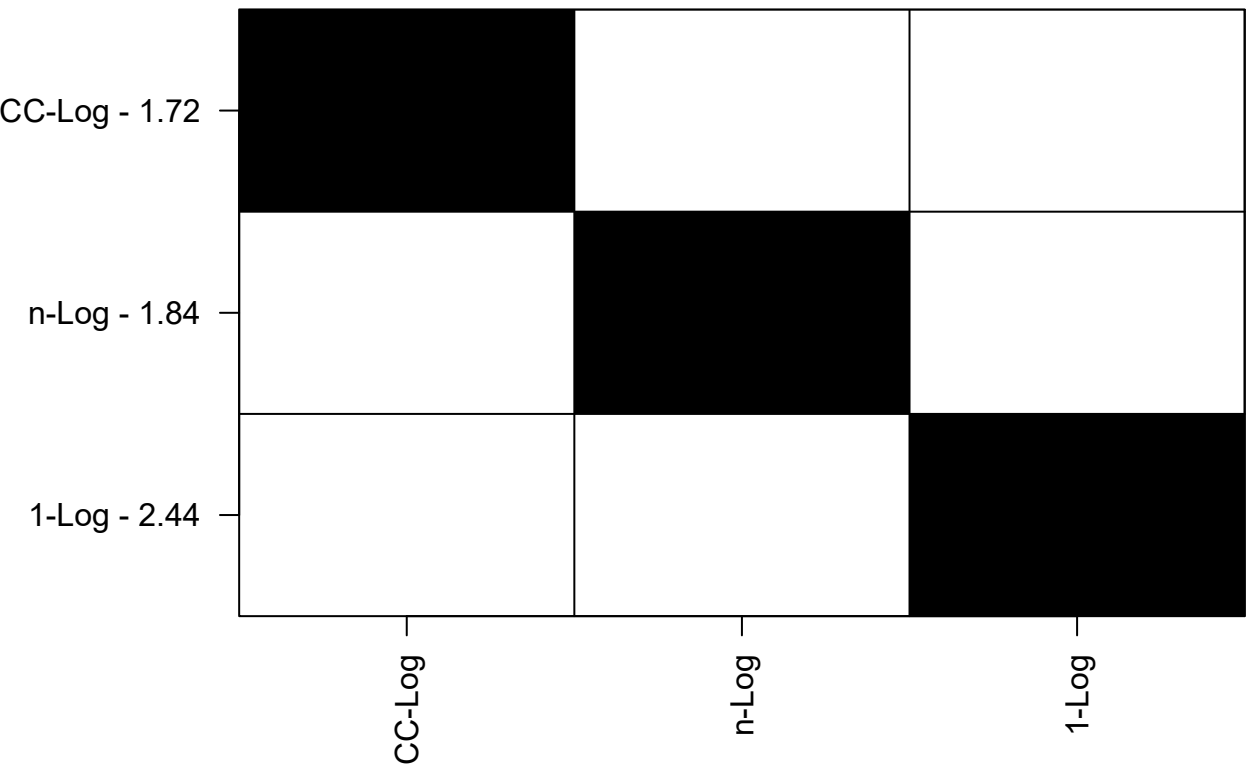

Supplement: S2 File — (R) [file pone.0236331.s002.zip › Graphics/Nemenyi_PPV_Fibre.pdf]

# Nemenyi test ranks using AUC

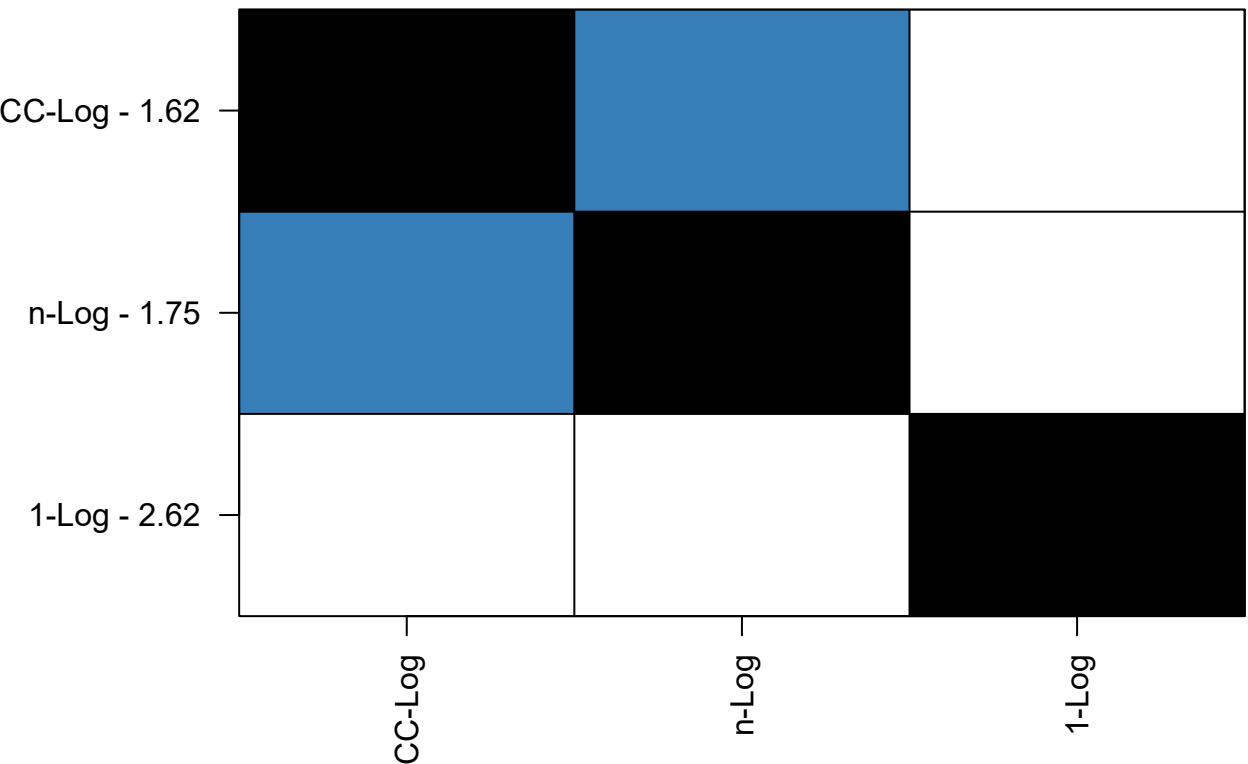

Supplement: S2 File — (R) [file pone.0236331.s002.zip › Graphics/Nemenyi_ROC_Fibre.pdf]

# Nemenyi test ranks for synthetic data

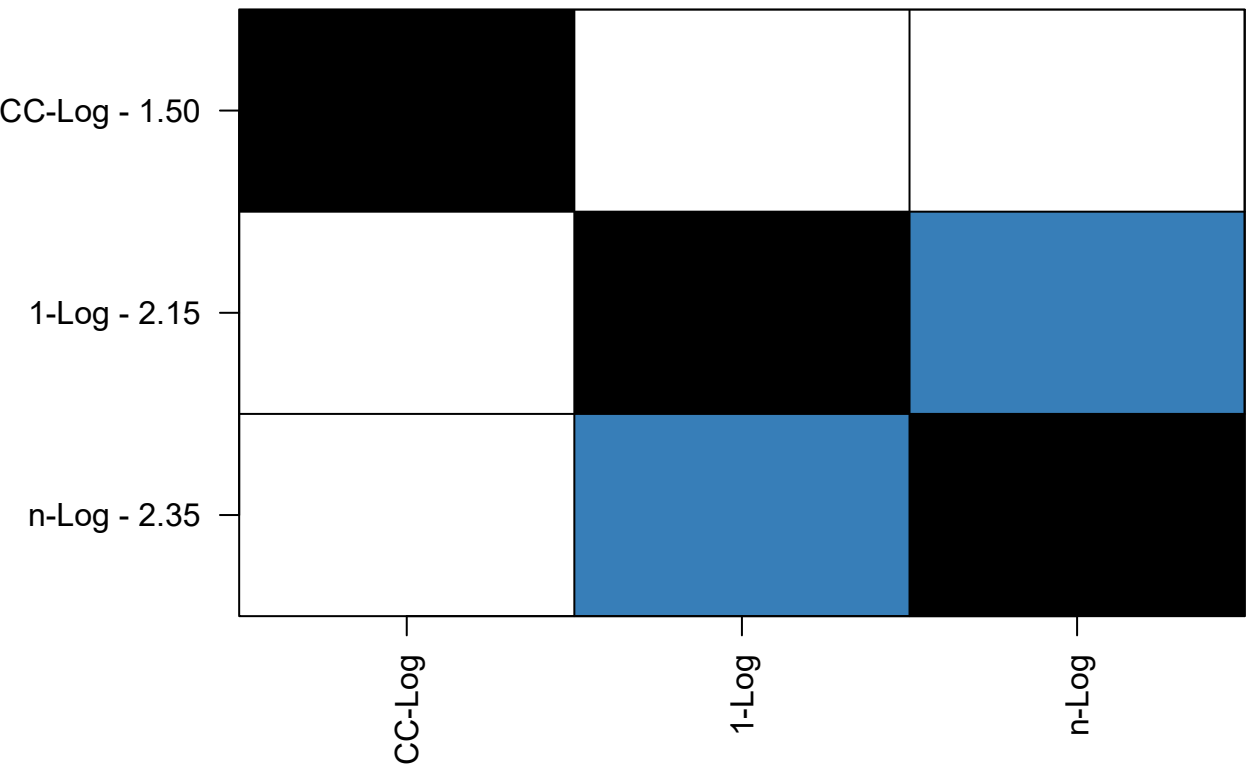

Supplement: S2 File — (R) [file pone.0236331.s002.zip › Graphics/Nemenyi_Synthetic_Accuracy.pdf]

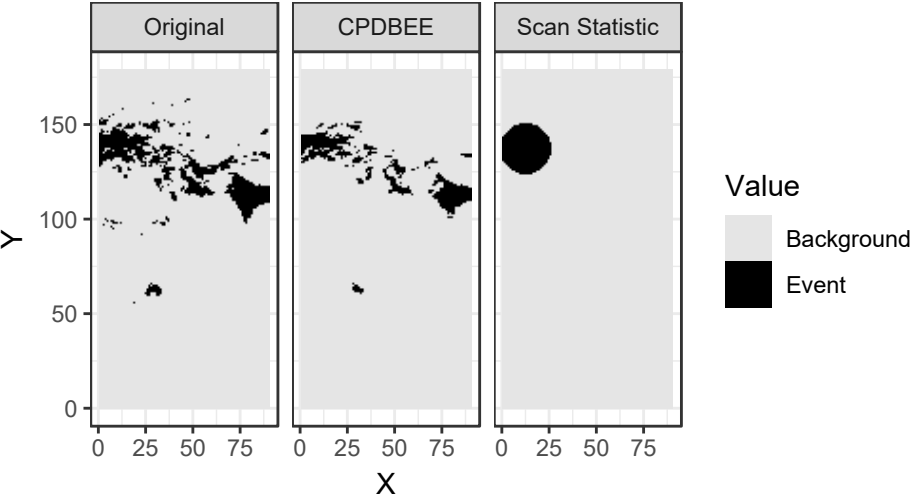

Supplement: S2 File — (R) [file pone.0236331.s002.zip › Graphics/NO2_Event_Comparison_181_270_2.pdf]

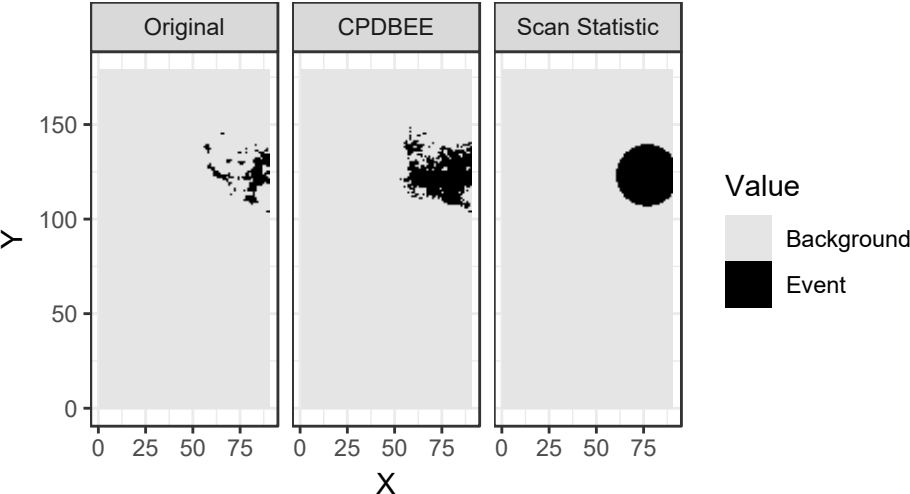

Supplement: S2 File — (R) [file pone.0236331.s002.zip › Graphics/NO2_Event_Comparison_1_90_2.pdf]

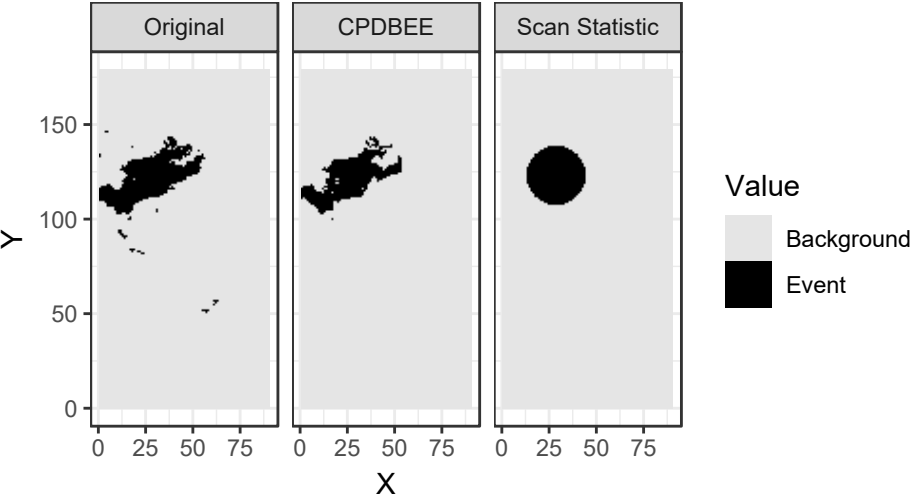

Supplement: S2 File — (R) [file pone.0236331.s002.zip › Graphics/NO2_Event_Comparison_271_360_2.pdf]

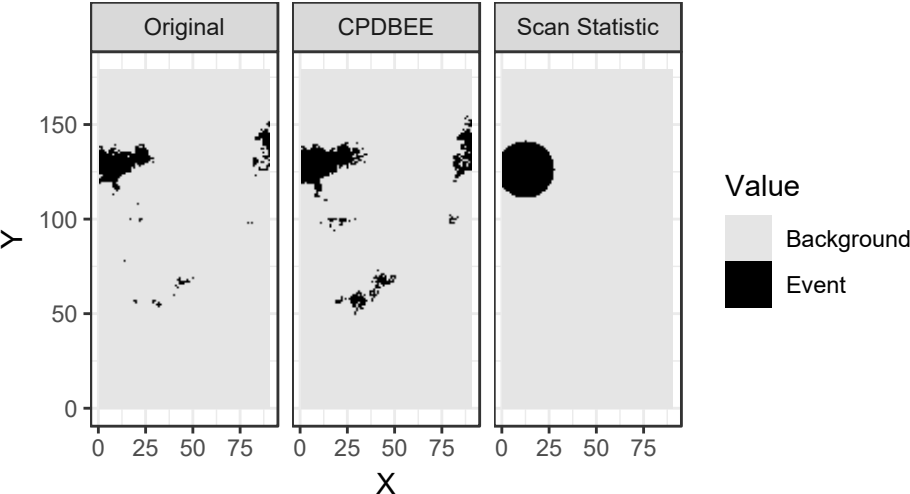

Supplement: S2 File — (R) [file pone.0236331.s002.zip › Graphics/NO2_Event_Comparison_91_180_2.pdf]

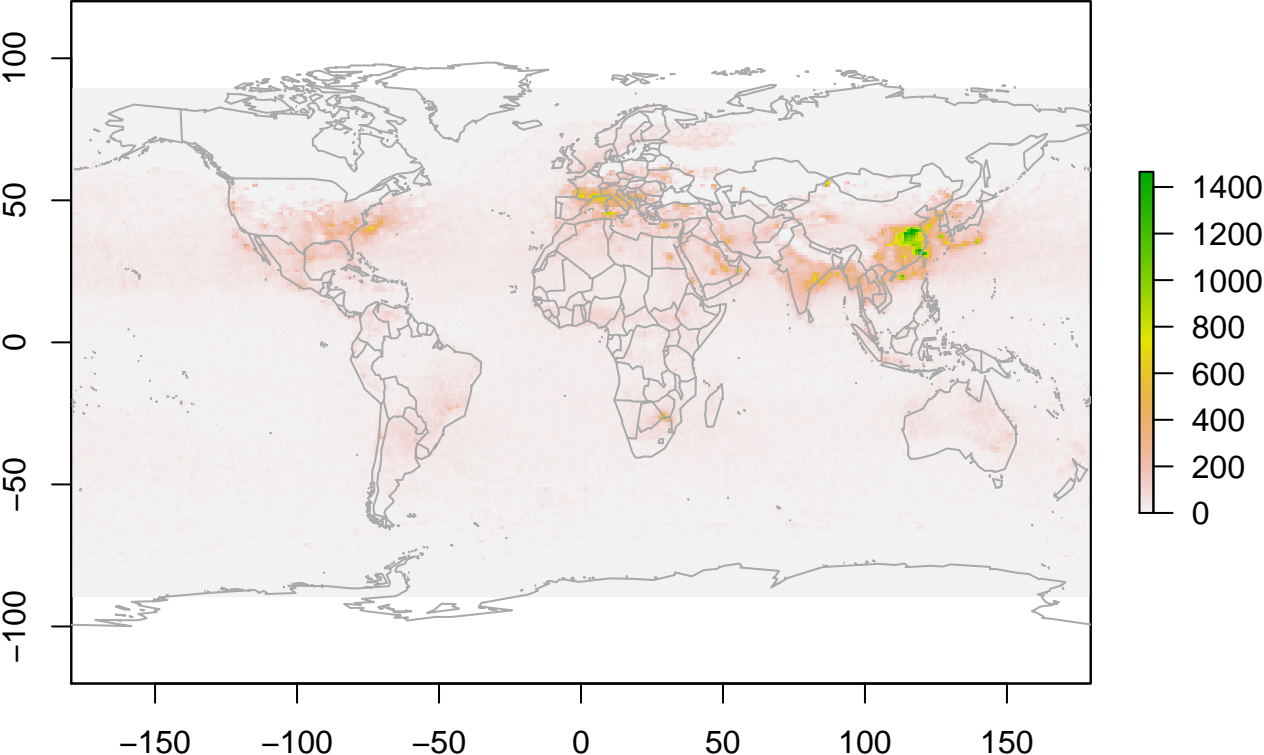

Supplement: S2 File — (R) [file pone.0236331.s002.zip › Graphics/NO2_March_2018_With_Bndry.pdf]

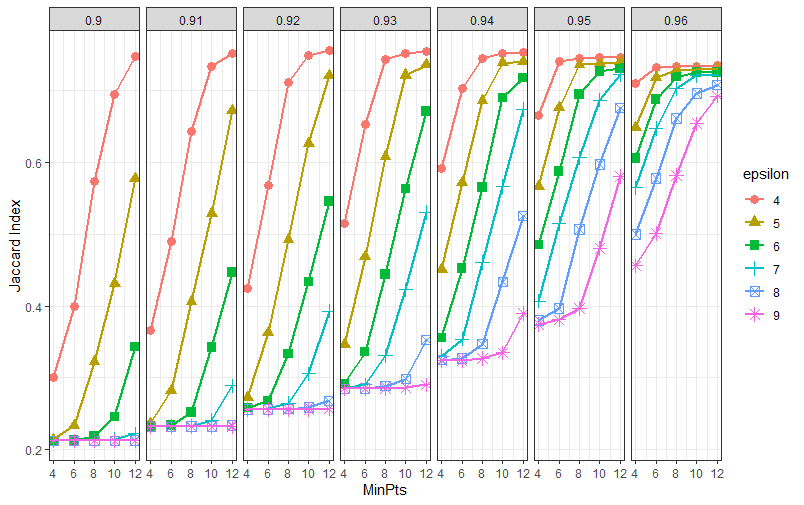

Supplement: S2 File — (R) [file pone.0236331.s002.zip › Graphics/Parameters.png]

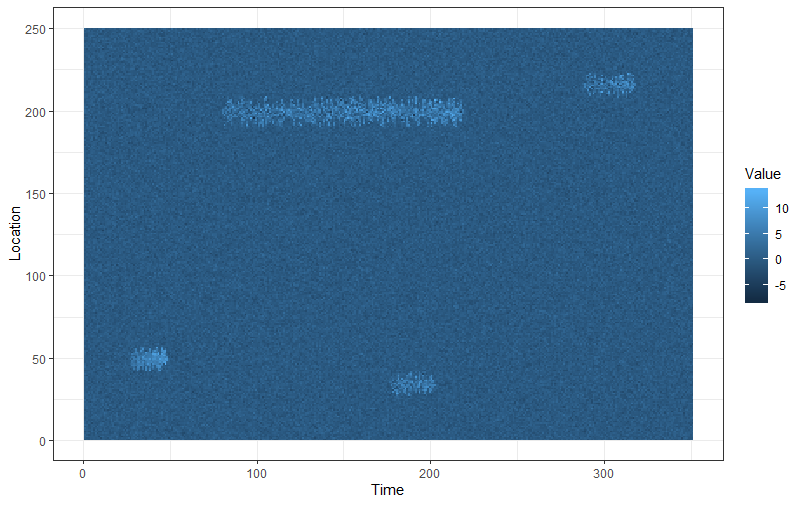

Supplement: S2 File — (R) [file pone.0236331.s002.zip › Graphics/Raw_Synth_1.png]

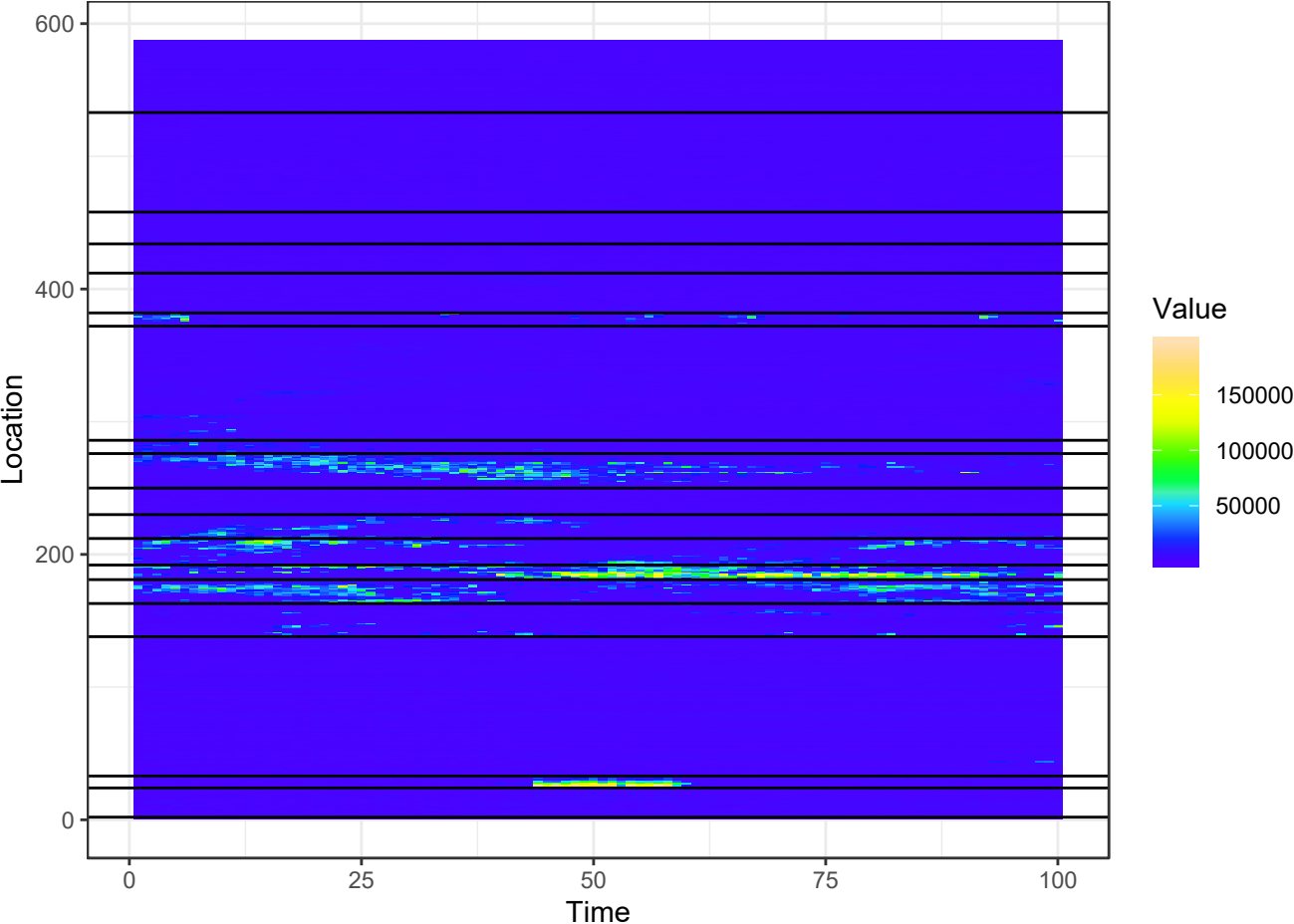

Supplement: S2 File — (R) [file pone.0236331.s002.zip › Graphics/real_data_location_cpts.pdf]

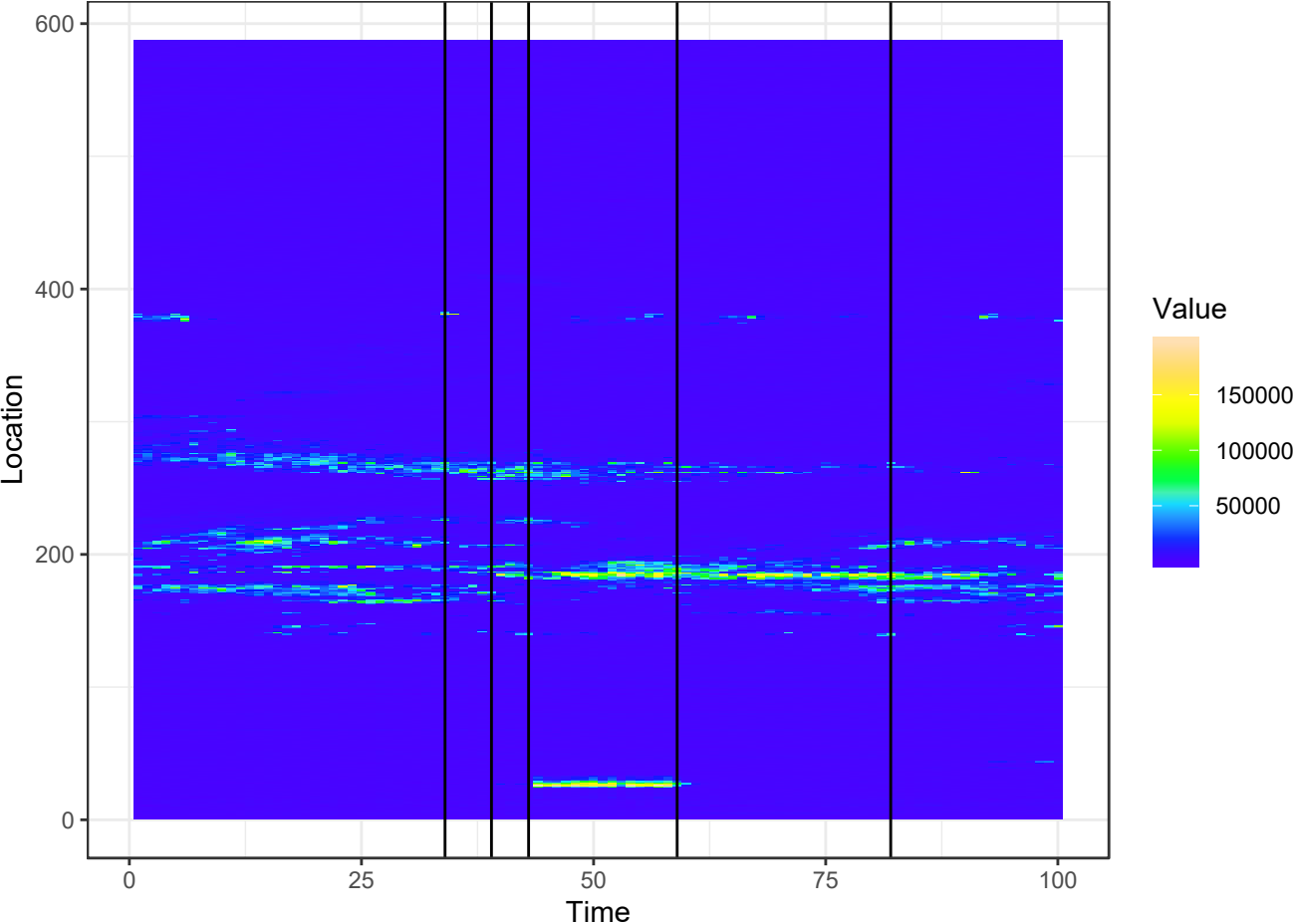

Supplement: S2 File — (R) [file pone.0236331.s002.zip › Graphics/real_data_time_cpts.pdf]

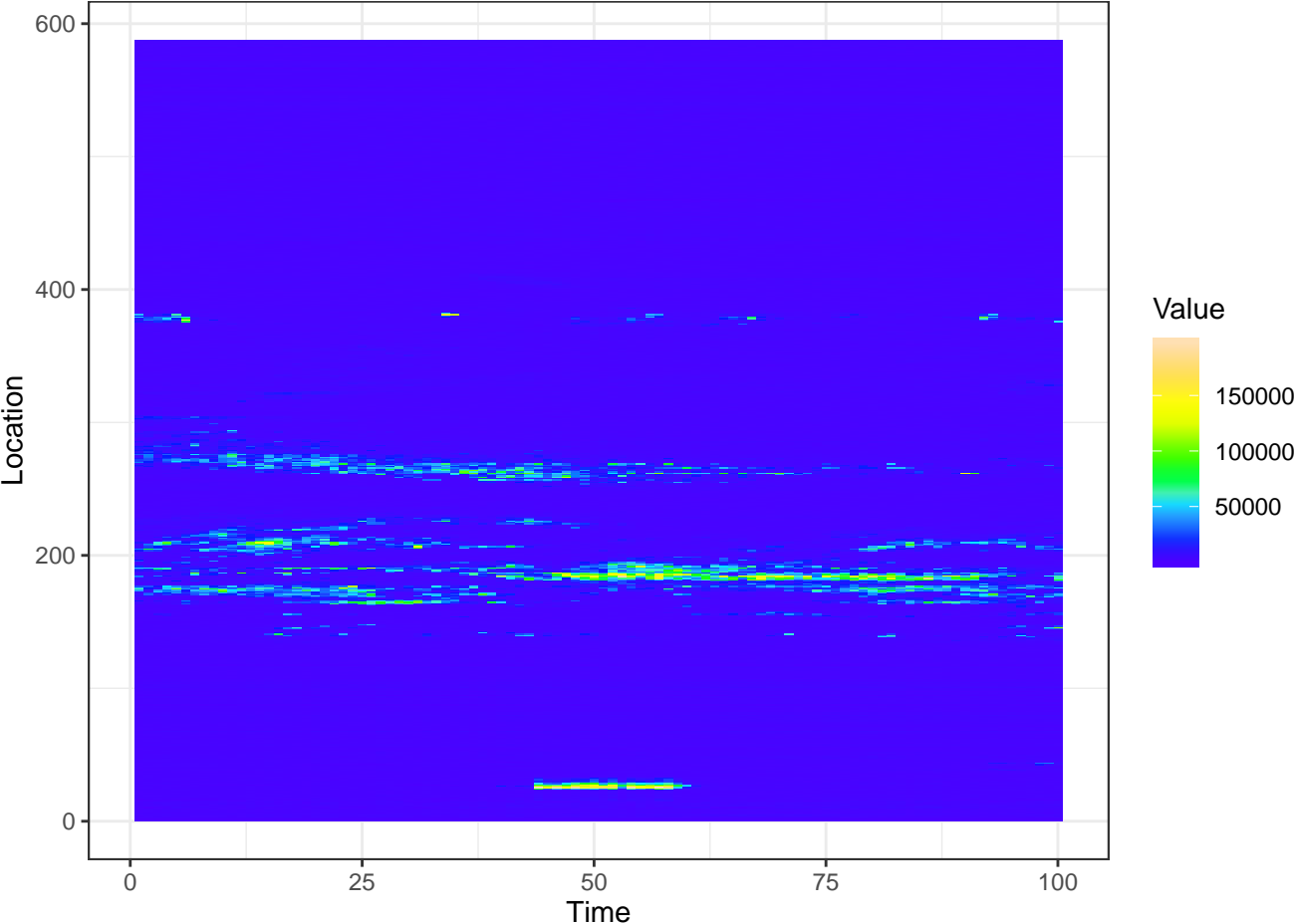

Supplement: S2 File — (R) [file pone.0236331.s002.zip › Graphics/Real_World.pdf]

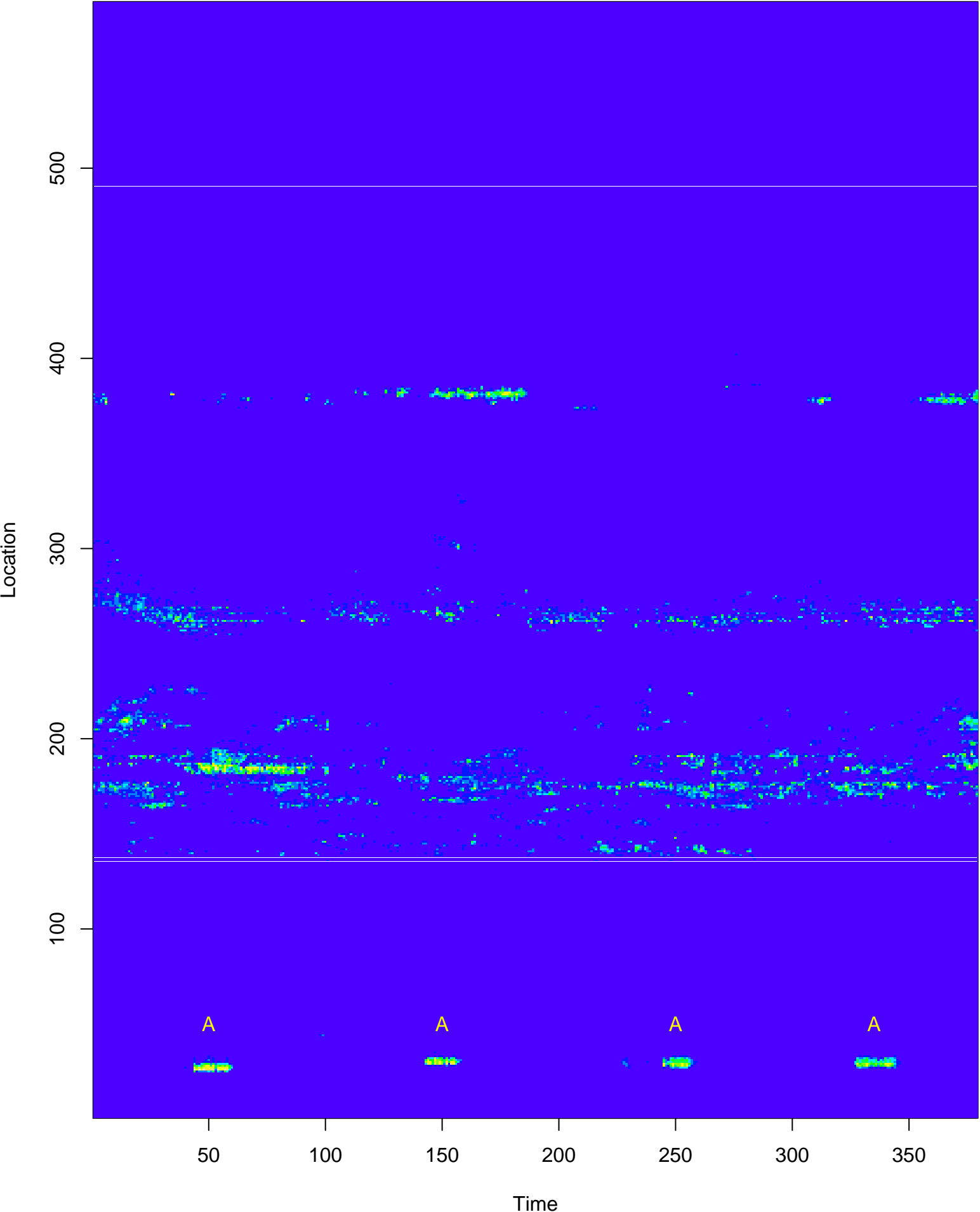

Supplement: S2 File — (R) [file pone.0236331.s002.zip › Graphics/Real_World_stream.pdf]

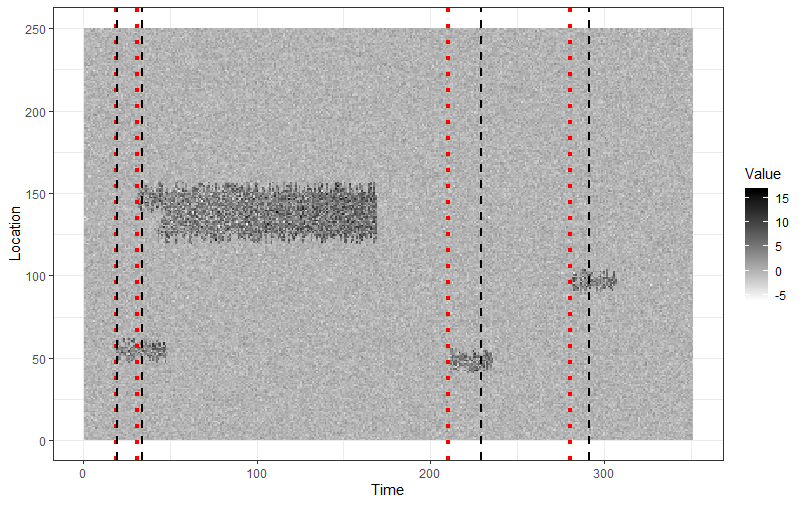

Supplement: S2 File — (R) [file pone.0236331.s002.zip › Graphics/sd_1234_clusters_detected_rolling_100.png]

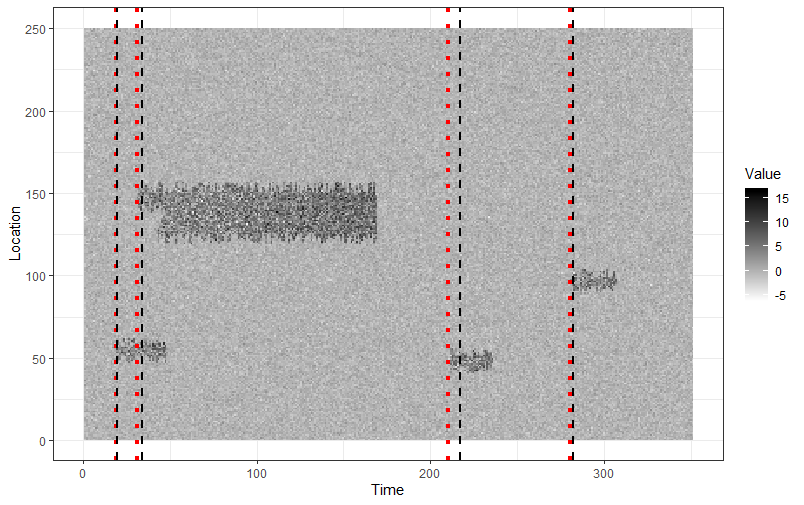

Supplement: S2 File — (R) [file pone.0236331.s002.zip › Graphics/sd_1234_clusters_detected_rolling_50.png]

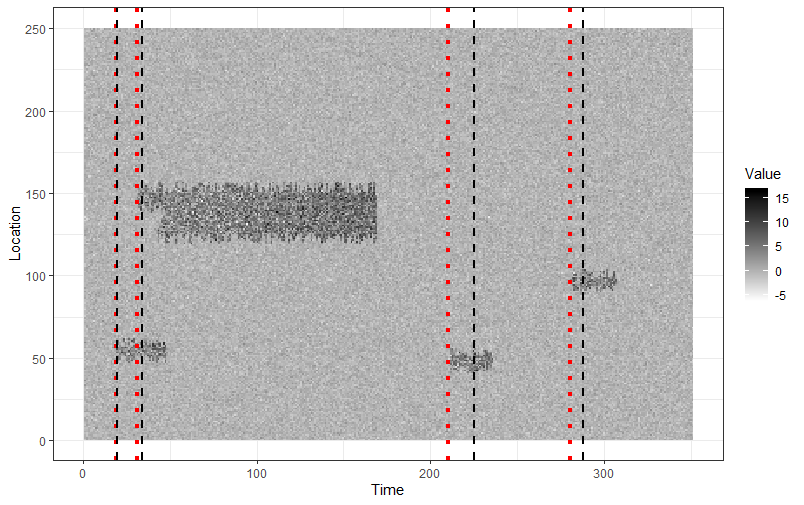

Supplement: S2 File — (R) [file pone.0236331.s002.zip › Graphics/sd_1234_clusters_detected_rolling_75.png]

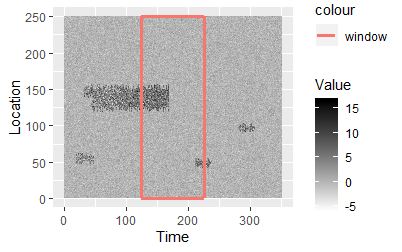

Supplement: S2 File — (R) [file pone.0236331.s002.zip › Graphics/sd_1234_with_window.png]

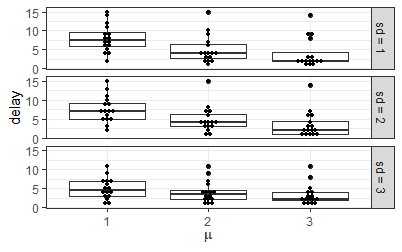

Supplement: S2 File — (R) [file pone.0236331.s002.zip › Graphics/sensitivity_event_detection.png]

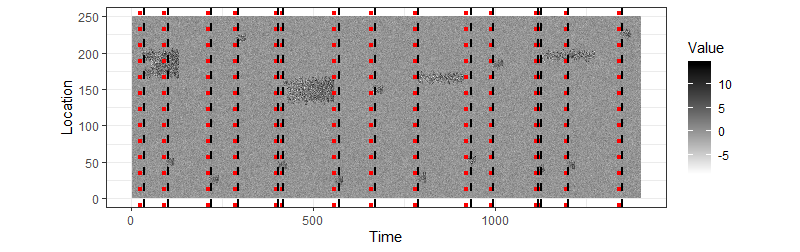

Supplement: S2 File — (R) [file pone.0236331.s002.zip › Graphics/sensitivity_mu_11_sd_11.png]

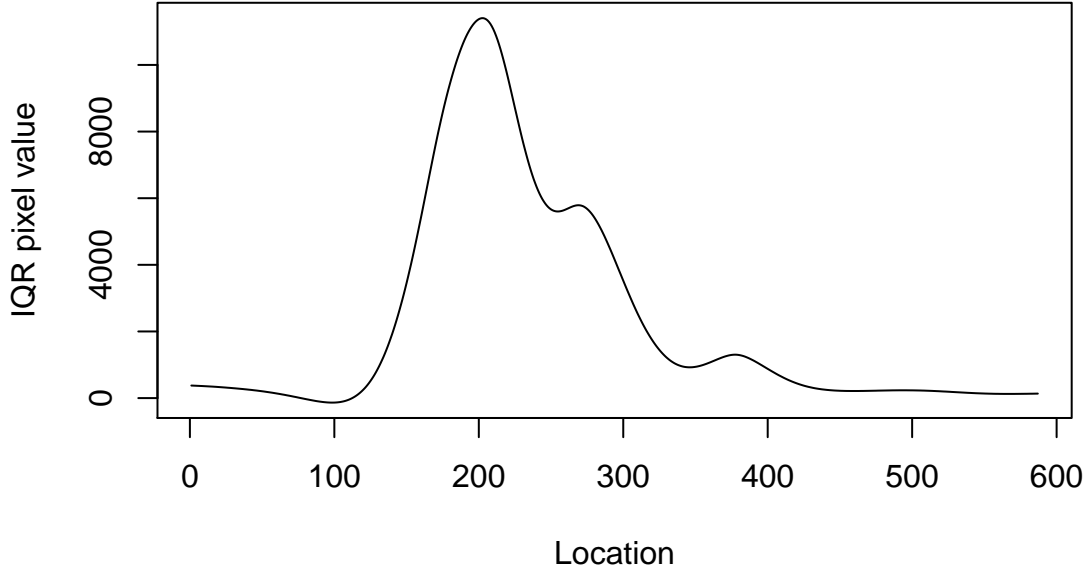

Supplement: S2 File — (R) [file pone.0236331.s002.zip › Graphics/Spline_IQR.pdf]

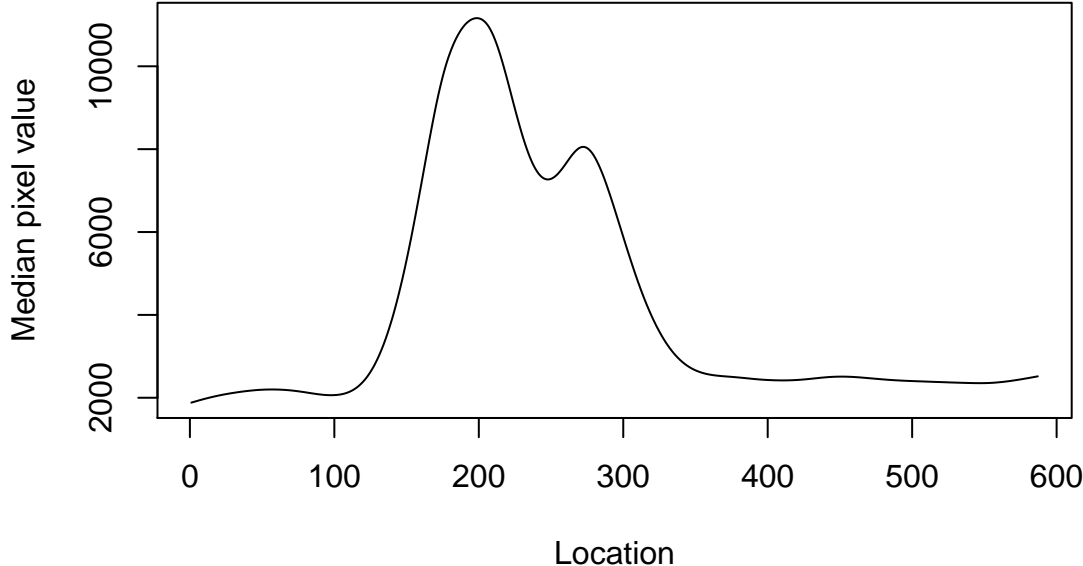

Supplement: S2 File — (R) [file pone.0236331.s002.zip › Graphics/Spline_Median.pdf]

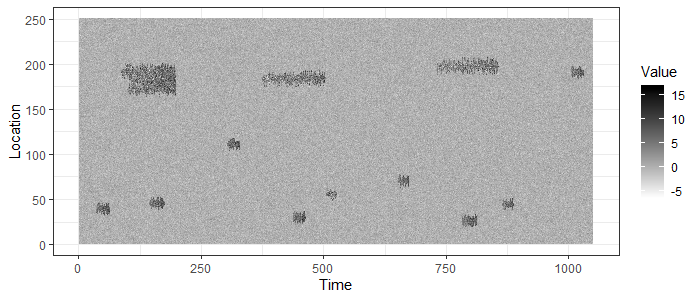

Supplement: S2 File — (R) [file pone.0236331.s002.zip › Graphics/synthetic_data_for_tradeoff.png]

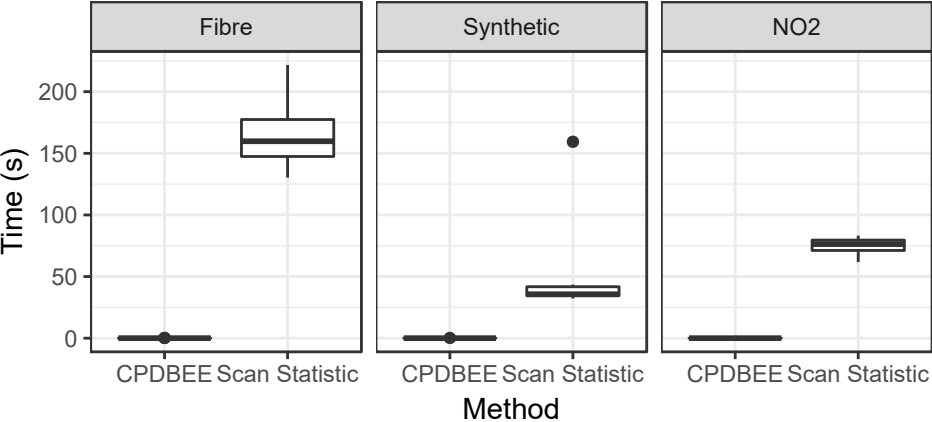

Supplement: S2 File — (R) [file pone.0236331.s002.zip › Graphics/Time_Comparison.pdf]

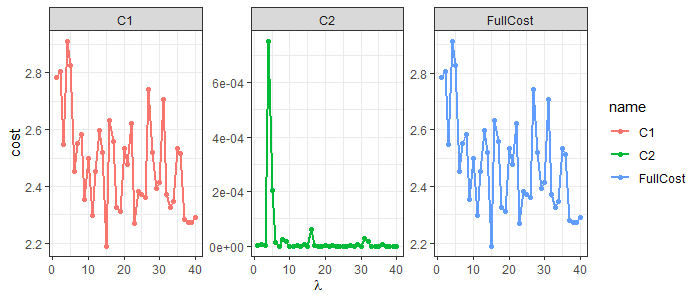

Supplement: S2 File — (R) [file pone.0236331.s002.zip › Graphics/tradeoff_lambda.png]

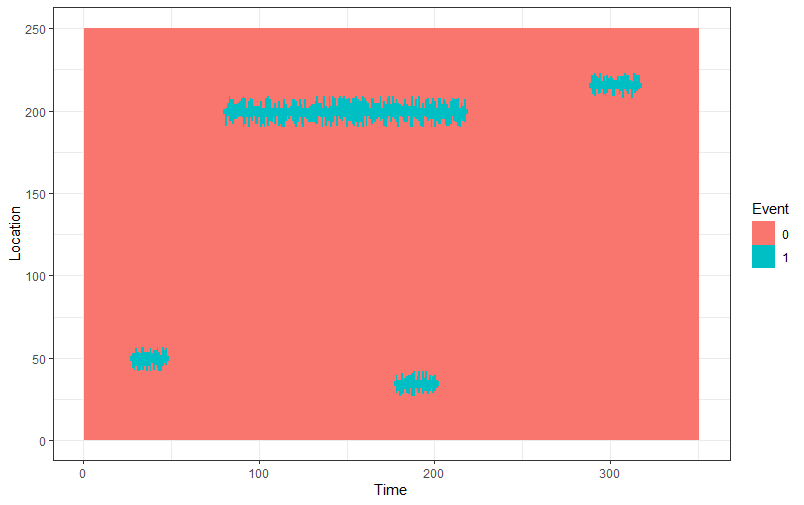

Supplement: S2 File — (R) [file pone.0236331.s002.zip › Graphics/True_Events_Synth.png]

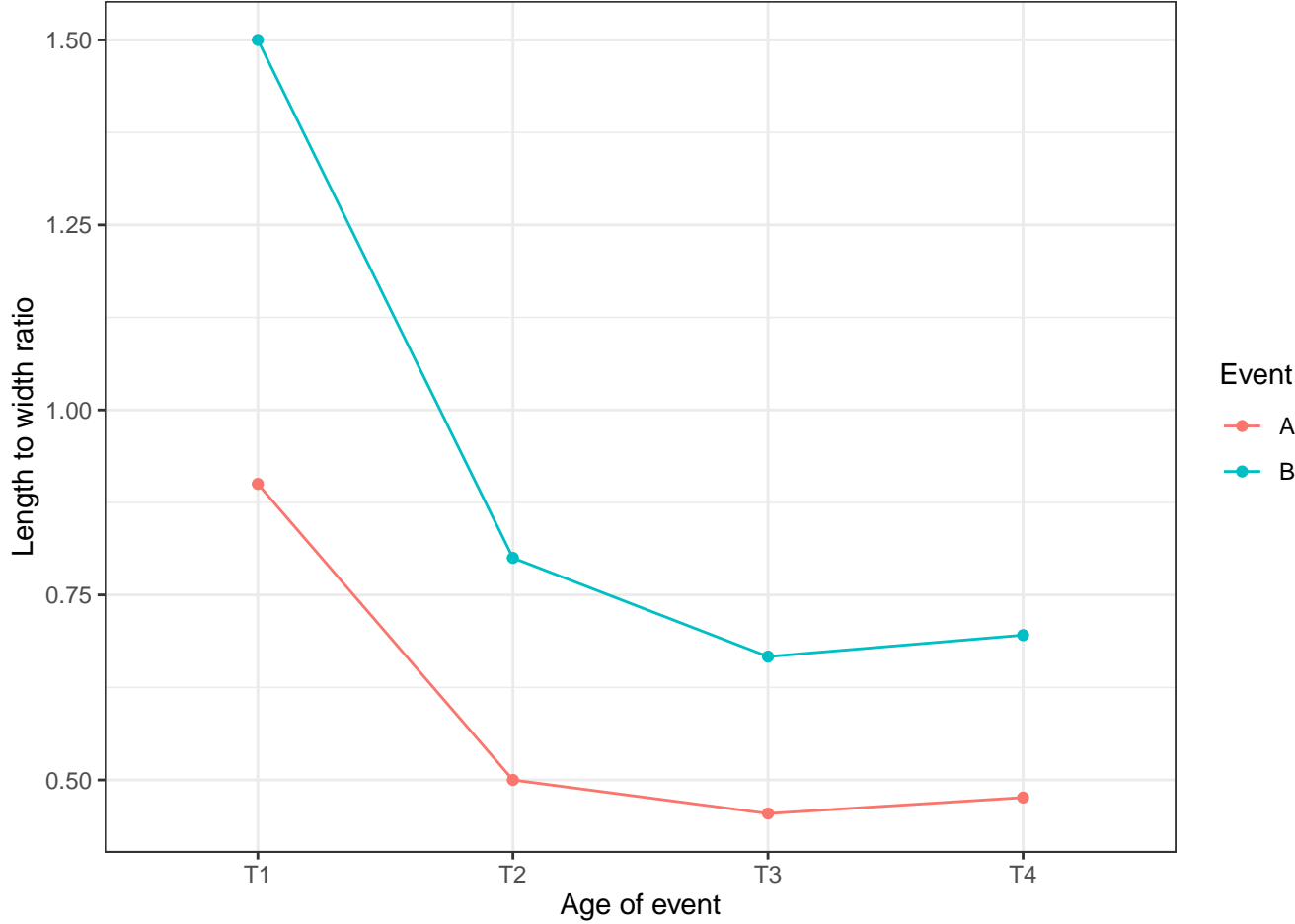

Supplement: S2 File — (R) [file pone.0236331.s002.zip › Graphics/Two_Event_Features.pdf]
