# Supplementary material for: Early classification of spatio-temporal events using partial information
Source: PLoS One. 2020 Aug 5;15(8):e0236331. doi: 10.1371/journal.pone.0236331 (PMC7406362; doi:10.1371/journal.pone.0236331)
Supplement: S1 Appendix — (PDF) [file pone.0236331.s007.pdf]

A Appendix

A.1 Event extraction results for fibre optic cable data

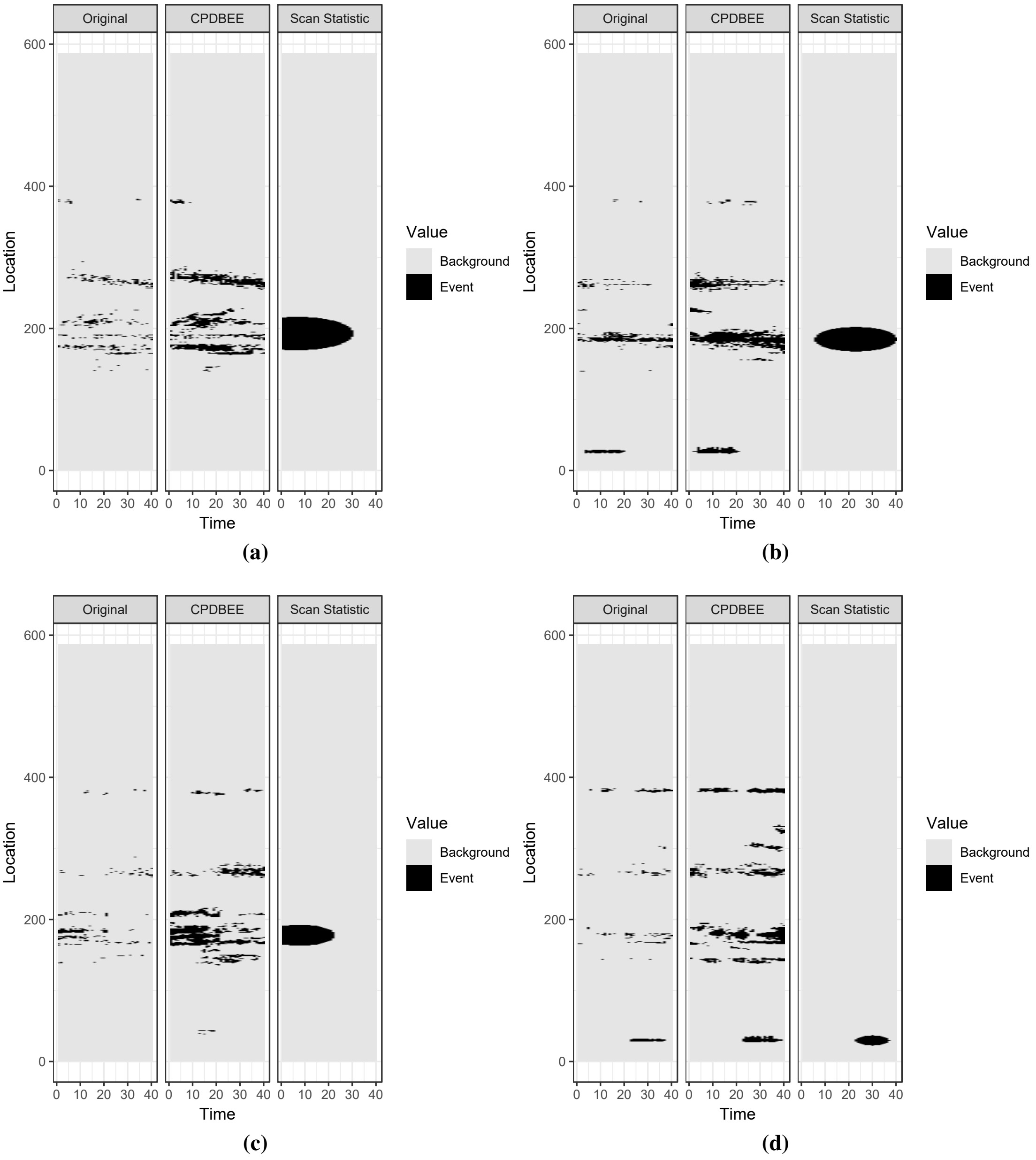

Figure 37: *Event extraction comparison for fibre optic data windows 1–4.*

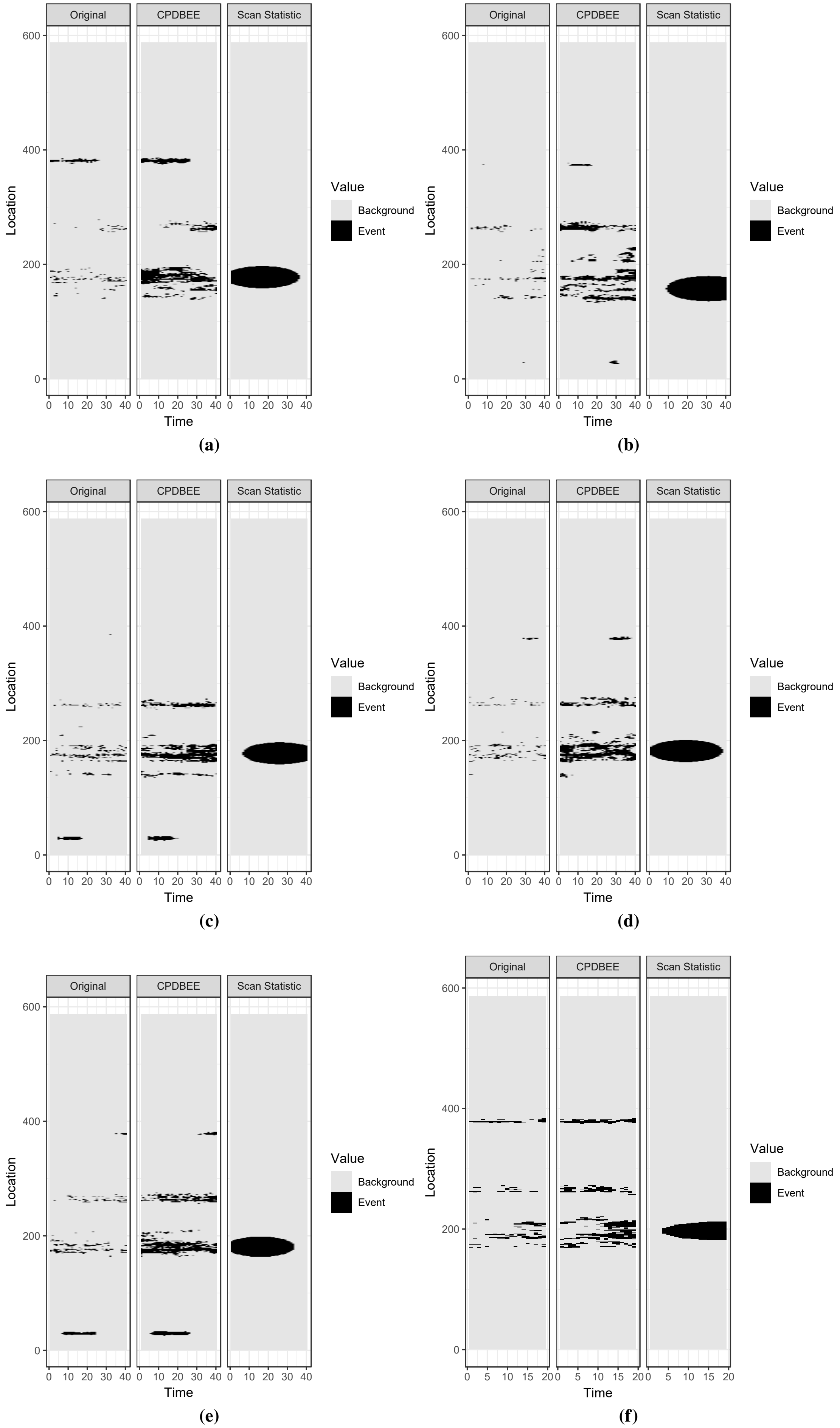

Figure 38: *Event extraction comparison for fibre optic data windows 5–10.*

A.2 Event extraction results for synthetic data

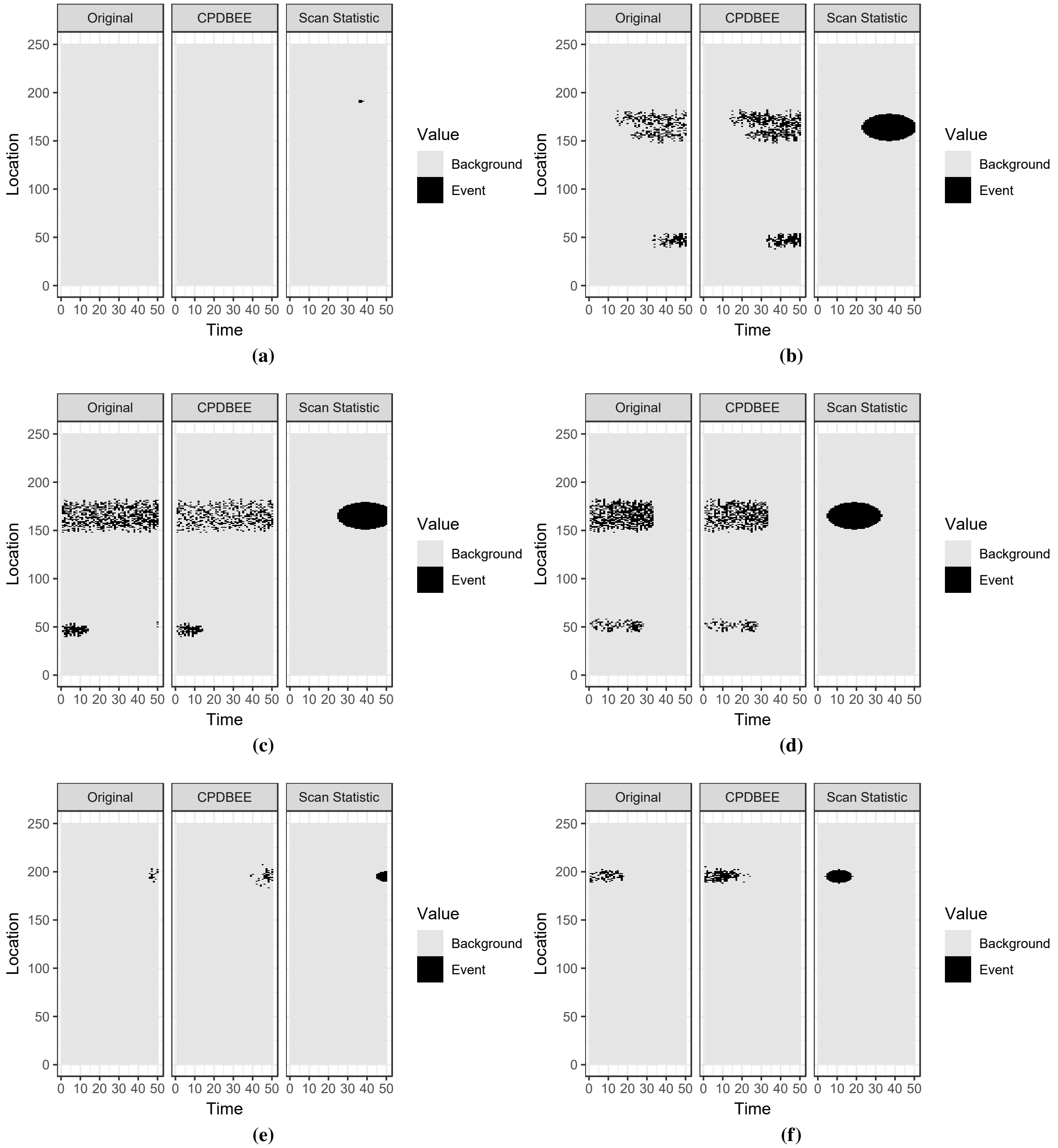

Figure 39: *Event extraction comparison for synthetic data.*

A.3 Event extraction results for NO_2 data

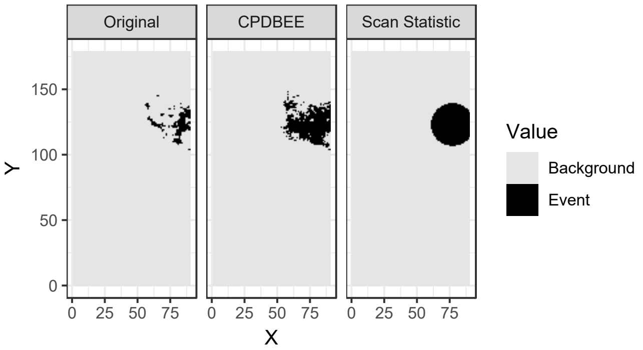

(a)

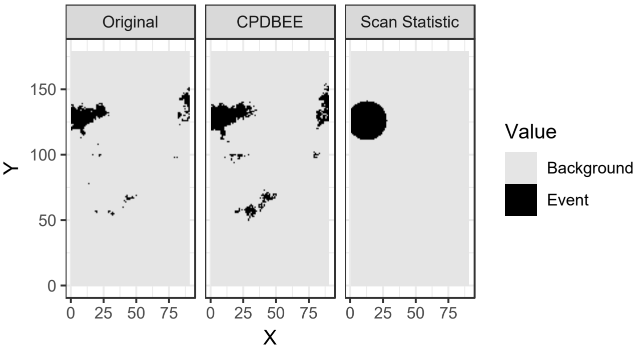

(b)

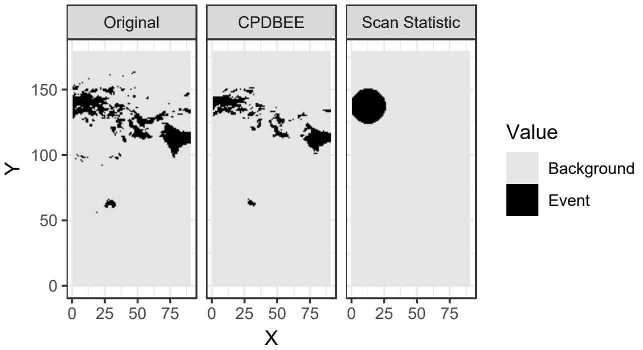

(c)

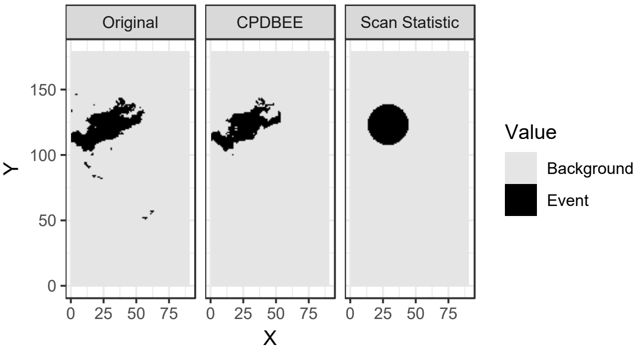

(d)

Figure 40: *Event extraction comparison for NO_2 data.*
